# Evolution of Electrogenic Ammonium Transporters (AMTs)

**DOI:** 10.3389/fpls.2016.00352

**Published:** 2016-03-31

**Authors:** Tami R. McDonald, John M. Ward

**Affiliations:** ^1^Biology Department, St. Catherine UniversitySt. Paul, MN, USA; ^2^Department of Plant Biology, University of MinnesotaSt. Paul, MN, USA

**Keywords:** ammonium transporters, electrophysiology, *Marchantia polymorpha*, phylogeny, electrogenicity

## Abstract

The ammonium transporter gene family consists of three main clades, AMT, MEP, and Rh. The evolutionary history of the AMT/MEP/Rh gene family is characterized by multiple horizontal gene transfer events, gene family expansion and contraction, and gene loss; thus the gene tree for this family of transporters is unlike the organismal tree. The genomes of angiosperms contain genes for both electrogenic and electroneutral ammonium transporters, but it is not clear how far back in the land plant lineage electrogenic ammonium transporters occur. Here, we place *Marchantia polymorpha* ammonium transporters in the AMT/MEP/Rh phylogeny and we show that AMTs from the liverwort *M. polymorpha* are electrogenic. This information suggests that electrogenic ammonium transport evolved at least as early as the divergence of bryophytes in the land plant lineage.

## Introduction

Genes in the ammonium transporter family are found in almost all prokaryotic and eukaryotic lineages. The gene family consists of three major clades, the AMT, MEP, and Rh and we refer to the family as the AMT/MEP/Rh family. Whereas the function of AMT/MEP/Rh proteins in microorganisms and plants is to uptake ammonium/ammonia, which for these organisms is an important nutrient used in the synthesis of N-containing metabolites such as amino acids, the function of AMT/MEP/Rh proteins in animals is to excrete ammonium/ammonia, a by-product which is toxic to animal cells. Despite their divergent physiological functions, the AMT/MEP/Rh proteins are structurally similar. Crystal structures of ammonium transporters from *Escherichia coli* and *Archaeoglobus fulgidus* indicate that these proteins have 11 transmembrane domains that fold into a pore (Khademi et al., 2004; Zheng et al., 2004; Andrade et al., 2005). The proteins trimerize in the membrane, forming a triple pore through which the substrate moves. Crystal structures from Rh proteins have demonstrated that these proteins have 12 transmembrane domains and likewise trimerize in the membrane to form a triple pore (Gruswitz et al., 2010).

Although the structures of AMT/MEP/Rh proteins have been elucidated, the transport mechanism and the substrate of these proteins remain hotly debated topics. At least three distinct transport mechanisms have been reported for proteins in the AMT/MEP/RH family. AmtB from *E. coli* and Amt1 from the archaean *A. fulgidus* are both found in the MEP clade. Crystal structures indicate that these proteins carry ammonia through the pore. An ammonium ion docks in an extracellular vestibule, the proton is stripped, the ammonia molecule transverses the pore, and the molecule is reprotonated on the intracellular side. As no charge is transported across the membrane, this transport is electroneutral. This interpretation has been corroborated with studies using labeled methylammonium (CH_3_NH${}_{3}^{+}$) (Soupene et al., 1998; Javelle et al., 2005, 2007).

By contrast, Rh proteins are generally understood to be passive channels transporting either CO_2_ or NH_3_, or perhaps both (Kustu and Inwood, 2006). From the crystal structure of the Rh from *Nitrosomonas europaea* the authors concluded that this protein most likely transports either NH_3_ or CO_2_ (Li et al., 2007; Lupo et al., 2007). A crystal structure of the human rhesus C glycoprotein RhCG is available (Gruswitz et al., 2010) and the lack of the canonical CO_2_ binding site was noted. The function of the human RhBG has been studied by radioactive methylammonium uptake and electrophysiology and determined to be electroneutral (Ludewig, 2004). Finally, some proteins encoded by the AMT/MEP/Rh family have been demonstrated to facilitate the movement of NH${}_{4}^{+}$ across the membrane, either as NH${}_{4}^{+}$ uniporters (Ludewig et al., 2002; Scherzer et al., 2013; Yang et al., 2015) or as NH_3_/H^+^ co-transporters (Neuhauser et al., 2014; Neuhauser and Ludewig, 2014). Because a net charge crosses the membrane, this type of transport is electrogenic. It is unclear whether all members of the large AMT/MEP/Rh family translocate NH_3_ or whether some translocate NH${}_{4}^{+}$.

**Figure 1:**
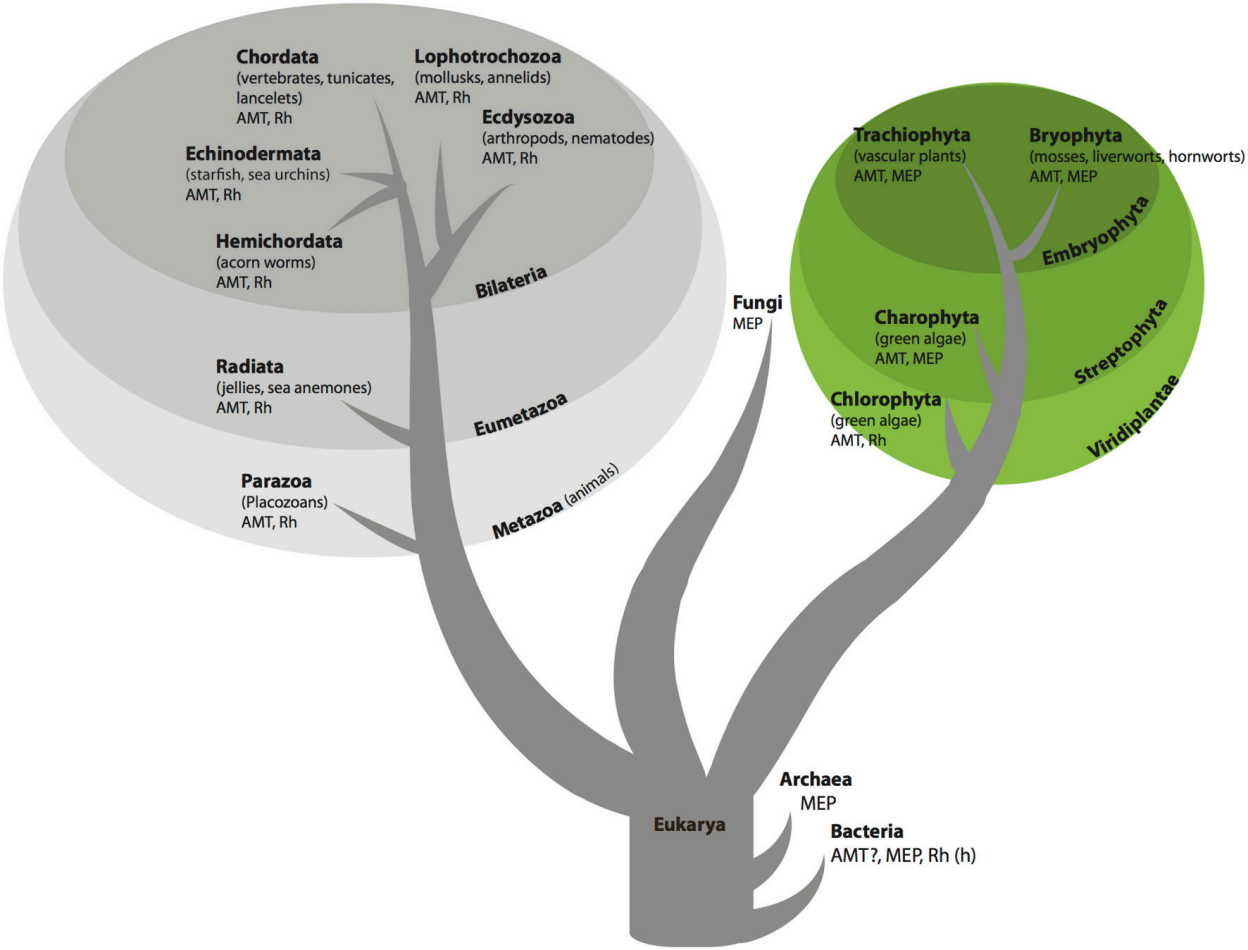
**Organismal tree of life showing presence of AMT/MEP/Rh family transporters**. In bacteria Rh is found only in a few species and probably was acquired by horizontal gene transfer as indicated by (h) and AMT sequences in bacteria have low support as indicated by the question mark (see text for details).

**Figure 2:**
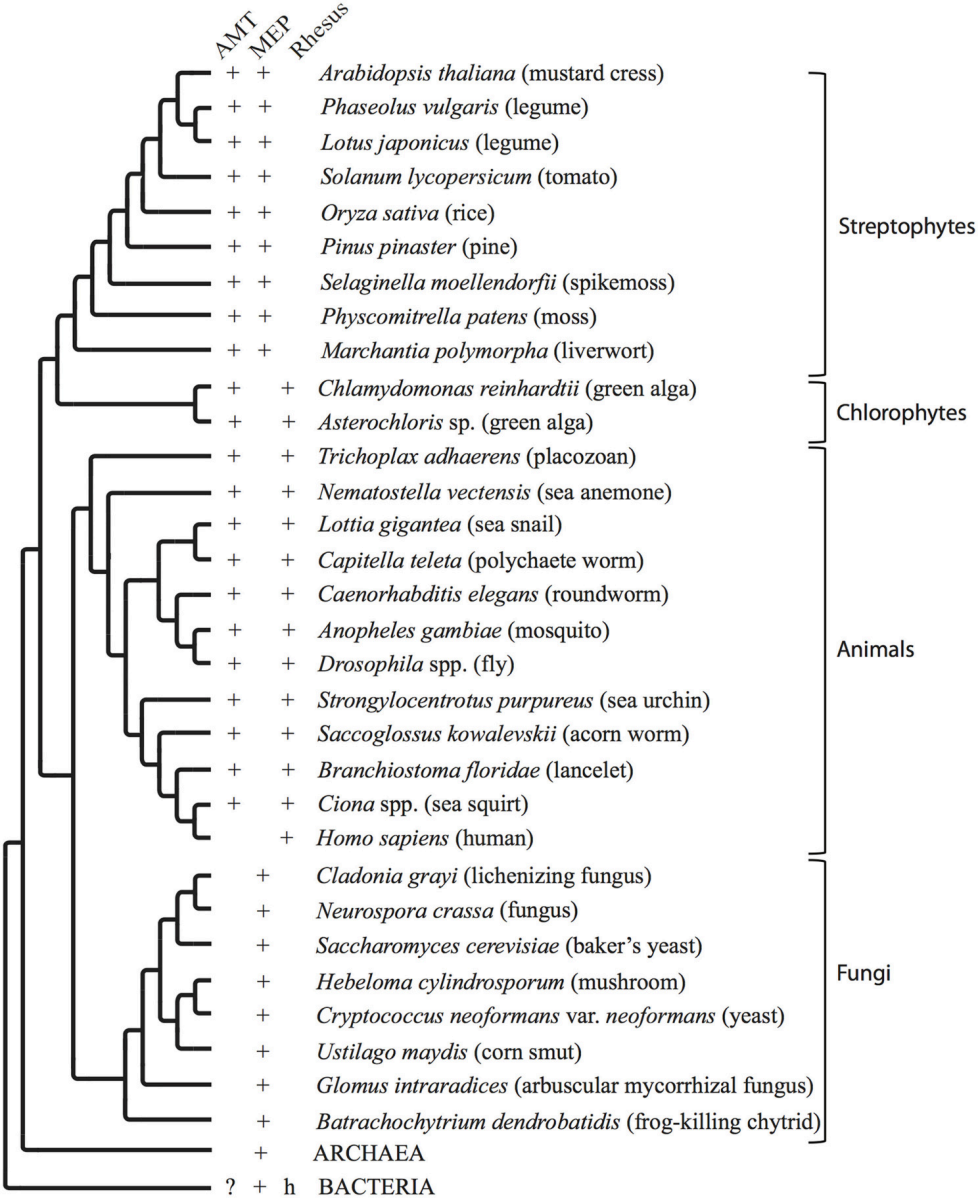
**Organismal phylogenetic tree**. Conceptual phylogenetic tree summarizing the relationships between organisms addressed in this paper and depicting the presence or absence in the genome of each type of ammonium transporter. The archaeal and bacterial branches are collapsed for clarity. AMT = ammonium transporter genes in the AMT clade. MEP = ammonium transporter genes in the MEP (methylammonium permease) clade. Rhesus = Rhesus factor proteins. + indicates one or multiple copies of a transporter of this type is/are present in the genome. ? indicates that genes from a limited number of species, such as *Nostoc* spp., recovered without support in this group of organisms. h indicates likely horizontal gene transfer into a limited number of species.

The AMT/MEP/Rh family has an evolutionary history riddled with expansion, contraction, gene loss and horizontal gene transfer (McDonald et al., 2012). Consequently the evolutionary history of the gene family does not mirror the evolutionary history of organisms. Individual genomes may contain more than one ammonium transporter gene, and these genes may have vastly different evolutionary histories and encode proteins with distinct functions. The AMT/MEP/Rh family has an ancient origin in prokaryotes (Figures 1, 2). Bacteria contain primarily MEP genes. Rh is found in only a few species of bacteria (Huang and Peng, 2005) and therefore was probably obtained through horizontal gene transfer. AMT presence in bacteria has little phylogenetic support. Fungi are unique among the eukaryotes in that their genomes only contain ammonium transporter genes from the MEP clade. Animal genomes, in general, encode ammonium transporters from both the AMT and Rh clades. Notable exceptions are vertebrates, including humans, whose genomes encode only ammonium transporters from the Rh clade (Figure 2). Among plants, the genomes of chlorophyte green algae contain AMT and often Rh genes, whereas the genomes of Charophyte green algae contain AMT and often MEP genes. Land plant genomes all contain both AMT (AMT1 family transporters) and MEP transporter genes (AMT2 family transporters; Figures 1, 2).

In plants, electrogenic ammonium transport has been demonstrated only in angiosperms. Among dicots, two-electrode voltage clamping has been used to demonstrate electrogenic transport for LeAMT1;1 and LeAMT1;2 from tomato (*Solanum lycopersicum*, formerly *Lycopersicon esculentum*) (Mayer et al., 2006a), for DmAMT1;1 from Venus flytrap (*Dionaea muscipula*) (Scherzer et al., 2013), for PvAMT1;1 from bean (*Phasoleus vulgaris*) which is both electrogenic and pH dependent (Ortiz-Ramirez et al., 2011) and AtAMT1;1 and AtAMT1;2 from mustard cress (*Arabidopsis thaliana*) (Mayer et al., 2006b). From monocots, electrogenic transport has been demonstrated in rice (*Oryza sativa* AMT1;1) (Yang et al., 2015). All of these transporters belong to the plant AMT family. The best characterized example of a plant MEP, *A. thaliana* AMT2 (AtAMT2), allows ammonium uptake when expressed in yeast (Sohlenkamp et al., 2000) but is electroneutral (Neuhäuser et al., 2009).

It is not known whether other embryophyte ammonium transporters are electroneutral or electrogenic. We hypothesize that all AMT proteins from land plants (AMT1 family proteins) are electrogenic. To test this hypothesis, we isolated ammonium transporter cDNAs from a basal land plant, the liverwort *Marchantia polymorpha* and tested their transport activity by expression in *Xenopus* oocytes and electrophysiology.

## Methods

### Identification of ammonium transporter genes

Putative *M. polymorpha* ammonium transporter genes and transcripts were bioinformatically identified by Dr. Ryuishi Nishihama using sequences from *Selaginella moellendorfii* as queries. Sequences were assembled in the program Sequencher, version 5.4.1 (Gene Codes Corporation, Ann Arbor, MI USA).

### Phylogenetic analysis

Manual alignments of nucleotide data were performed using Mesquite 3.02 (Maddison and Maddison, 2015). Ambiguously aligned regions and introns were delimited manually and excluded from phylogenetic analyses. Models of molecular evolution were selected using the Akaike Information Criterion (AIC) implemented in ProtTest (Abascal et al., 2005; Posada, 2008). Protein sequences and accession numbers are provided in Supplemental File 1. Phylogenetic relationships and confidence values were inferred using a maximum likelihood approach at both the nucleotide and protein levels, and the results from the protein analysis are reported here. Maximum likelihood analyses were performed using the program RAxML-HPC2 version 8.1.24 (Stamatakis, 2014) implemented through the CIPRES Science Gateway (Miller et al., 2010). For the tree topology search, 100 distinct randomized MP trees were generated from the original alignment and 100 inferences were made on these trees under the gamma model of rate heterogeneity with a ML estimate of the alpha-parameter, using the LG substitution matrix (Le and Gascuel, 2008) and empirical frequencies, followed by model optimizations of the resulting ML trees. A final GAMMA-based thorough optimization of the best-scoring ML tree was performed. Bootstrapping was performed in the same program using 1000 replicates. Trees were visualized using FigTree version 1.3.1 (Rambaut, 2009) and Dendroscope version 3.3.2 (Huson and Scornavacca, 2012).

### Cloning and cRNA synthesis

*M. polymorpha* male accession Takaragaike-1 (Tak-1) (Okada et al., 2000) was obtained from Drs. Ryuichi Nishihama and Takayuki Kohchi (Kyoto University). RNA was extracted from *Marchantia* thalli using the RNeasy kit (Qiagen). cDNA was synthesized using the Omniscript Reverse Transcription kit (Qiagen). Primers were designed to amplify the coding regions of five MpAMTs. PCR products were cloned into pCR8/GW/TOPO (Invitrogen) and sequences were confirmed. These clones were recombined with the oocyte expression vector pOO2/GW (Sun et al., 2010).

Constructs in pOO2/GW were linearized using MluI, and cRNA was synthesized using the SP6 mMessage mMachine kit (Ambion). *Xenopus laevis* oocytes were injected with 50 ng (50 nl) of cRNA per oocyte except for MpAMT1;4 which was injected at a rate of 1.6 ng per oocyte. Oocytes were incubated in Barths solution [88 mM NaCl, 1 mM KCl, 0.33 mM Ca(NO_3_)_2_, 0.41 mM CaCl_2_, 0.82 mM MgSO_4_, 2.4 mM NaHCO_3_, 10 mM HEPES, pH 7.6, 100 μg ml^−1^ penicillin, and 100 μg ml^−1^ streptomycin] containing 10 μg ml^−1^ gentamycin for 3–4 d at 15°C.

### Electrophysiology

Electrophysiology measurements were made using a Dagan TEVC 200 amplifier (Dagan Corp.). Currents were recorded using Clampex (Axon Instruments Inc.). Thin wall 1.5 mm borosilicate glass pipettes (Warner Instruments Corp.) filled with 1 M KCl were used for recordings. Oocytes were perfused with K-free Ringer solution (115 mM NaCl, 2 mM CaCl_2_, 2 mM MgCl_2_, 5 mM MES, pH 5.6 with NaOH) and clamped at a holding potential of −40 mV. Substrates were applied as chloride salts in the same K-free Ringer solution.

## Results

### Phylogenetic analysis of *Marchantia polymorpha* AMTs

**Figure 3:**
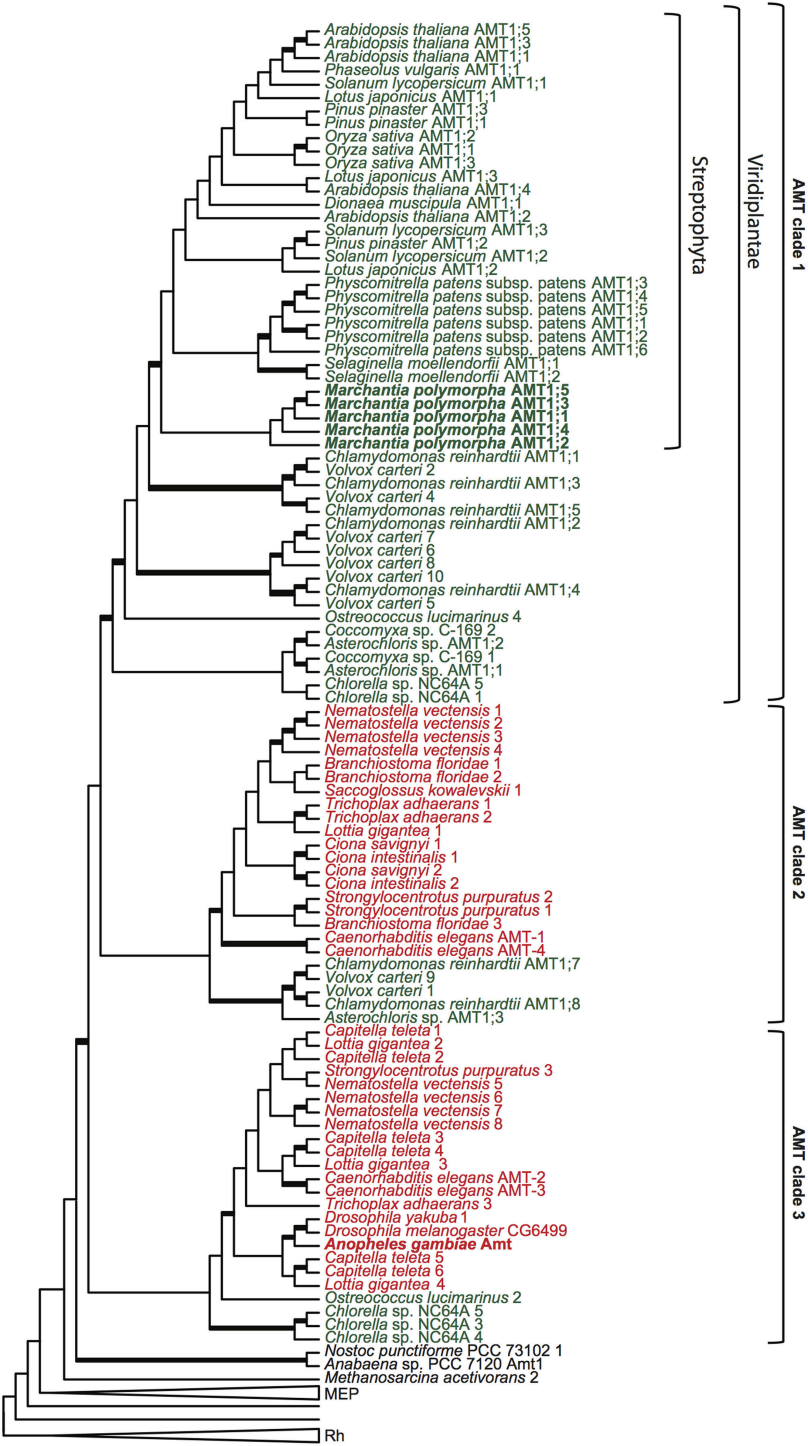
**Phylogenetic analysis of ammonium transporter proteins**. Maximum likelihood tree of AMT/MEP/Rh proteins. Thickened branches represent greater that 70% bootstrap support. The genome of each organism represented was searched for AMT/MEP/Rh genes, all full-length genes were aligned and then translated into amino acid sequences. Where possible, published protein names are used, otherwise each protein from a given organism is numbered according to its the placement on the tree. The MEP and the Rh clades have been collapsed. The AMT transporters cluster into three clades, denoted Clade 1, Clade 2, and Clade 3. The placements of transporters from the liverwort, *Marchantia polymorpha*, and mosquito, *Anopheles gambiae*, are highlighted in bold. Transporters from plants and algae are in green type, and transporters from animals are in red type.

From the transcript library, several partial and six full-length ammonium transporter genes were identified. Primers were designed to amplify the coding region of all full-length putative ammonium transporter genes. Full-length cDNA sequences were cloned, and three clones of each full-length cDNA were sequenced for confirmation. Phylogenetic analyses of ammonium transporters including the six full-length *M. polymorpha* sequences were performed. A maximum likelihood tree of protein sequences is shown in Figure 3 and Supplemental Figure 1. In this tree, three main clades are depicted. The term AMT refers to the clade containing ammonium transporter AtAMT1;1 from *A. thaliana* (Ninnemann et al., 1994). We use the term MEP to refer to the clade containing the methylammonium transporter MEP1 from yeast (Marini et al., 1994), which also encompasses the plant AMT2 family of electroneutral transporters. Additional transporters appear in the phylogeny at the base of the AMT/MEP groups, and this set of transporters is referred to here as the MEP grade. The Rh clade contains Rh factor proteins found in animals (Marini et al., 1997), early-diverging eukaryotes, some prokaryotes, and green algae.

Five of the full-length *M. polymorpha* ammonium transporter sequences were found in the AMT clade, while one of the transporters was located in the MEP clade. The phylogenetic position of the transporters in the AMT clade indicates that these could be electrogenic transporters. If so, it would push back the origin of electrogenic transport from the advent of the angiosperms to the emergence of land plants (embryophytes), or about 300 million years earlier.

### Electrogenic ammonium transport by *Marchantia polymorpha* AMTs

**Figure 4:**
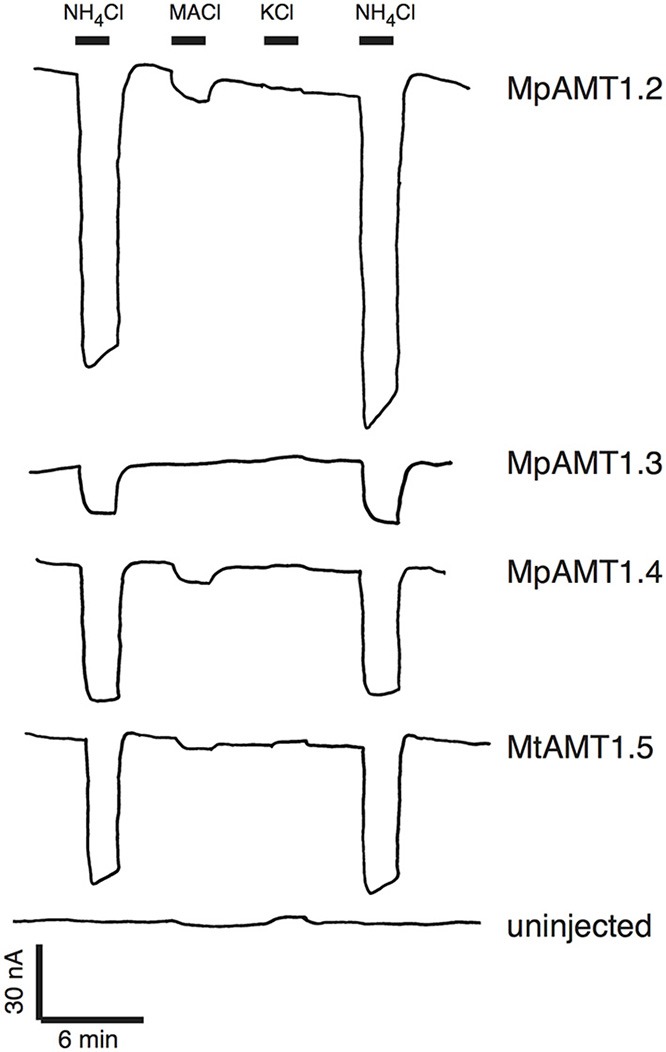
**Electrogenic ammonium transport by AMTs from *Marchantia polymorpha***. MpAMT cDNAs were expressed Xenopus oocytes. The oocytes were bathed in K^+^-free ringer solution (pH 5.6), voltage clamped at −40 mV and substrates were applied where indicated. Concentrations of substrates were 300 μM except that 50 μM NH4Cl was used for MpAMT1.5. MACl is methyl ammonium chloride.

To determine whether *M. polymorpha* AMT transporters were indeed electrogenic, four of the five AMT cDNAs were cloned from the male *Marchantia* accession Tak-1 (Okada et al., 2000) and expressed in *Xenopus* oocytes. Two-electrode voltage clamping was used to measure substrate-induced currents. Perfusion of 300 μM NH_4_Cl over the oocytes expressing *Marchantia* ammonium transporters produced large inward currents consistent with an electrogenic mechanism of ammonium transport (Figure 4). Very large currents were produced in oocytes expressing MpAMT1;4 and MpAMT1;5. Therefore, to reduce the level of transport activity, MpAMT1;4 RNA was injected at a 30-fold lower concentration, and ammonium chloride was applied at a reduced concentration of 50 μM for oocytes expressing MpAMT1;5 (Figure 4). Methylammonium chloride induced small inward currents indicating that MpAMTs may have a higher affinity for ammonium compared to methyl ammonium. Potassium chloride induced small outward currents consistent with the activation of endogenous Na/K-ATPase activity. The results are consistent with a high selectivity of the MpAMTs for ammonium over K^+^. Under our recording conditions, no ammonium- or methylammonium-dependent currents were observed in uninjected oocytes (Figure 4). However, application of 300 μM KCl induced small outward currents also in uninjected oocytes indicating that the outward currents are not associated with expression of the MpAMTs (Figure 4). The results show that AMTs from the basal plant *M. polymorpha* are electrogenic. The results support the hypothesis that all plant ammonium transporters in the AMT clade (AMT1s) are electrogenic.

## Discussion

### Evolution of electrogenic transport

In the current understanding of green plant evolution, liverworts are the earliest diverging group of land plants. Because the AMT transporters in the liverwort *Marchantia* are electrogenic, as are all the AMT transporters from monocots and dicots which have been tested to date using electrophysiology, the evolution of electrogenic transporters must have occurred at least as early as the transition of plants onto land. Thus, it is likely that land plant AMTs are all electrogenic transporters. It remains unclear, however, whether AMT transporters from earlier diverging lineages of Viridiplantae are electrogenic or not, as there is no data from the streptophycean green algae like *Coleochaete* or *Chara*, which are closely related to land plants, or from the chlorophycean green algae such as *Chlamydomonas*. Searches of genome and transcriptome data from various groups of streptophyte green algae and chlorophyte green algae reveal that many although not all streptophyte green algae have both AMT and MEP genes in their genomes. However, MEP genes do not appear to be represented in the genomes and transcriptomes that are currently available from chlorophyte green algae, although AMT genes and often Rh genes are present.

How early did electrogenic AMTs evolve? One possibility is that the electrogenic transporters arose in the plant clade at or around the time of the entrance of MEPs in the Streptophyte lineage. In this scenario, the horizontal transfer of electroneutral MEPs would have allowed the AMTs to neofunctionalize from ancestral electroneutral transporters into electrogenic transporters. The available electrophysiology and crystal structure data for transporters in the MEP clade suggest that land plant MEPs are electroneutral, consistent with this model.

However, it is also possible that AMTs outside of the land plant lineage are electrogenic. Unfortunately, electrophysiology data is largely lacking for other non-plant eukaryotic AMT transporters. One important exception is the AgAmt transporter from the mosquito *Anopheles gambiae*, which has been shown to be electrogenic (Pitts et al., 2014). Because the AgAmt transporter is found in clade 3 of this analysis (Figures 3), it is possible that all AMTs outside of the land plant lineage, up to and including the AMTs in clades 1, 2, or 3 and perhaps also the cyanobacterial transporters which appear without support at the base of the AMT clade, are electrogenic.

It is also possible that electrogenic transport arose independently in the plant and the animal lineage. Looking at the distribution of AMT genes from animal lineages in the AMT phylogeny, two clades of animal AMTs are evident (Figure 3). Most animals have multiple AMT transporter genes and at least one AMT in both animal AMT clades. However, the insect genomes sampled here contain only one ammonium transporter gene, and it is present in clade 3 in this phylogeny. There is not yet electrophysiology data for any of the transporters in AMT clade 2. It is reasonable to invoke parsimony to suggest that electrogenic transport is the ancestral state for the AMT clade at least as far back as the divergence of clade 3, and therefore to infer that the transporters in clade 2 are also electrogenic. However, it is also formally possible that the distribution of electrogenic transporters could be explained by independent origins of electrogenic transport in the streptophyte lineage and in the animal lineage. Neofunctionalization could have been precipitated in the streptophytes by the horizontal gene transfer of MEP transporters, and in the animal lineage by a gene duplication at least as far back as the origin of early-diverging animals such as placozoans. Electrophysiological data from transporters in clade 2 is required to exclude this possibility.

At this juncture, however, it seems most likely that electrogenic transport evolved once early within or at the base of the AMT clade. As AmtB and Amt-1 from the prokaryotes *E. coli* and *A. fulgidus* are thought to be electroneutral and the Rh proteins are mostly electroneutral, electroneutrality may be the ancestral state for AMT/MEP/Rh transporters and electrogenicity may have evolved later.

### Transport mechanism of AMTs and MEPs in plants: Electroneutral vs. electrogenic

Considering that plants obtained MEP genes through horizontal gene transfer and maintained both MEPs and the AMTs that were already present, there appears to be a strong advantage to retaining both types of transporters. One possible reason for the maintenance of two types of ammonium transporters is that they function differently. Whether ammonium transporters are electrogenic or electroneutral greatly affects their physiological function. Electrogenic ammonium transporters in the plasma membrane are more likely to function in net ammonium uptake because cation uptake (either NH${}_{4}^{+}$ or the H^+^ coupling ion) is driven by the negative membrane potential. In contrast, electroneutral ammonium transporters are primarily driven by the transmembrane ammonium concentration gradient.

Extreme care must be taken when using *Xenopus* oocytes and electrophysiology to determine whether ammonium transporters are electrogenic (as discussed in Ludewig, 2004). *Xenopus* oocytes contain NH${}_{4}^{+}$-permeable channels that are activated by millimolar concentrations of ammonium via cytoplasmic alkalinization. The activity of expressed electroneutral NH_3_ transporters causes cytoplasmic alkalinization and therefore activation of the endogenous NH${}_{4}^{+}$-permeable channels at lower external ammonium concentrations. Experimental conditions of total ammonium concentration of less than 1 mM and low pH, which limits the concentration of NH_3_ in solution, can be used to avoid activating endogenous NH${}_{4}^{+}$-permeable channels.

### Mechanisms of electrogenic transport in AMTs

**Figure 5:**
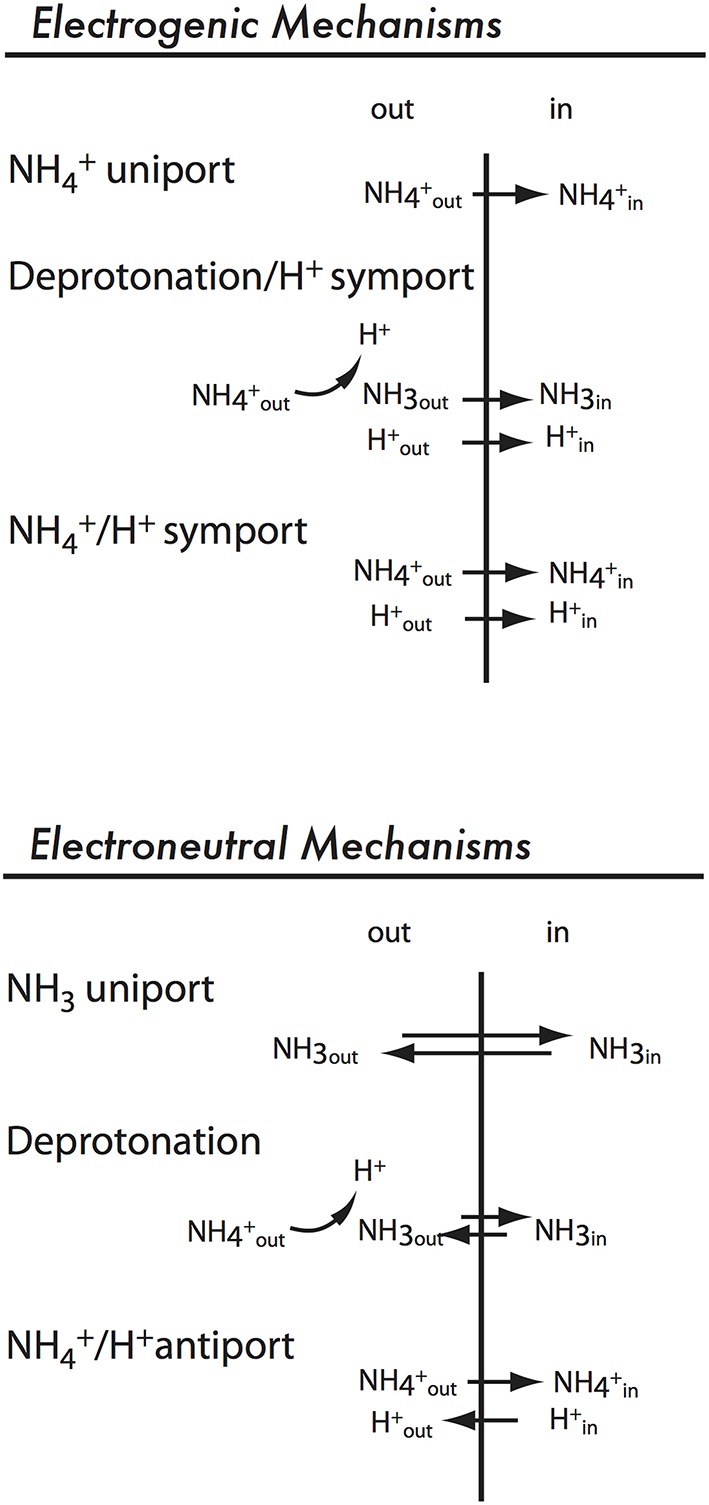
**Possible electroneutral and electrogenic mechanisms for AMT/MEP/Rh proteins**.

Three possible mechanisms for electrogenic ammonium transport are presented in Figure 5. “NH${}_{4}^{+}$ uniport” refers to direct transport of the cation NH${}_{4}^{+}$ and could represent either a channel activity or a transporter that operates with a conformational change for each NH${}_{4}^{+}$ transported. “Deprotonation/H^+^ symport” (Figure 5) refers to a mechanism in which the transporter binds NH${}_{4}^{+}$ and subsequently deprotonates it and translocates NH_3_. The charge is carried by the coupling ion (H^+^). A third possible mechanism is NH${}_{4}^{+}$/H^+^ symport (Figure 5; Ortiz-Ramirez et al., 2011).

Based on the pH-independence of ammonium-induced currents, plant AMTs have been described as uniporters for NH${}_{4}^{+}$ (Ludewig et al., 2002; Scherzer et al., 2013; Yang et al., 2015). Proton-coupled symporters are expected to be stimulated by low pH and show pH optima for activity in the acidic pH range but there are examples of *bona fide* H^+^-coupled symporters that show inhibition by acidic pH. For example, unlike other H^+^-coupled sucrose transporters, AtSUC9 from *Arabidopsis* shows the highest activity at pH 6.3 (Sivitz et al., 2007) rather than at more acidic pH. This was interpreted as negative regulation of activity of AtSUC9 at acidic pH and the same could be true for electrogenic AMTs that show pH-independent activity.

It is likely that plant AMTs are not NH${}_{4}^{+}$ uniporters, as more recent evidence indicates a H^+^-coupled mechanism (Neuhauser et al., 2014; Neuhauser and Ludewig, 2014). Energetically and electrophysiologically, this mechanism is not easily differentiated from the NH${}_{4}^{+}$ uniport model. The deprotonation/H^+^ symport model is supported by the identification of AtAMT1;2 mutants in which ammonium transport was uncoupled from cation uptake (Neuhauser and Ludewig, 2014). Mutations in AtAMT1;2 that resulted in electroneutral ammonium transport did not cause amino acid changes in the pore region but in more peripheral regions involved in subunit contact. This implies that NH_3_ is translocated in the pore and H^+^ translocation occurs via a different pathway affected by the mutations (Neuhauser et al., 2014; Neuhauser and Ludewig, 2014).

A second line of evidence supporting a deprotonation/H^+^ symport model for electrogenic ammonium transporters involves the high selectivity of plant AMTs for ammonium over cations of similar size such K^+^ and Na^+^ (Ludewig et al., 2002). However, the larger molecule CH_3_NH${}_{3}^{+}$ is transported by plant AMTs. Both NH${}_{4}^{+}$ and CH_3_NH${}_{3}^{+}$ can be deprotonated, but not Na^+^ or K^+^, suggesting that deprotonation is part of the transport mechanism. This argument, based on ammonium transporter selectivity, was used to support the deprotonation model for electroneutral transport by AmtB from *E. coli* (Winkler, 2006), but it is equally valid for a discussion of electrogenic AMTs.

However, there is evidence that not all plant AMTs share the same transport mechanism. PvAMT1;1 from bean is electrogenic and pH dependent (Ortiz-Ramirez et al., 2011). A mutation was found that inhibits pH dependence but does not affect electrogenic ammonium transport. Therefore, the mechanism was suggested to be NH${}_{4}^{+}$/H^+^ symport (Figure 5) (Ortiz-Ramirez et al., 2011). However, a mechanism NH_3_/2H^+^ for PvAMT1;1 is also consistent with the reported transport activity.

### Mechanisms of electroneutral transport in MEPs

Plants also encode ammonium transporters in the MEP clade. The best characterized example, *A. thaliana* AMT2 (AtAMT2), allows ammonium uptake when expressed in yeast (Sohlenkamp et al., 2000) but is electroneutral (Neuhäuser et al., 2009). Possible mechanisms for electroneutral ammonium transport are shown in Figure 5. The simplest model, “NH_3_ uniport,” is ruled out due to the low concentration of NH_3_ compared to NH${}_{4}^{+}$ at physiological pH. Prior to the availability of protein structures of *E. coli* AmtB (Khademi et al., 2004; Zheng et al., 2004), opinion was divided whether a “Deprotonation” or “NH${}_{4}^{+}$/H^+^ antiport” mechanism (Figure 5) was used by electroneutral ammonium transporters. We now know that the hydrophobicity of the pore is consistent with translocation of NH_3_ rather than NH${}_{4}^{+}$. Ammonium is bound at a high-affinity NH${}_{4}^{+}$ binding site on the extracellular side of the pore, it is deprotonated, and NH_3_ is translocated in the pore (reviewed by Winkler, 2006). The extracellular NH${}_{4}^{+}$ binding site is conserved in AtAMT2;1 (Neuhäuser et al., 2009), suggesting a similar mechanism for plant electroneutral ammonium transporters.

### Shared transport mechanism of AMTs and MEPs in plants: NH_3_ translocation

Considering the high degree of sequence identity in the AMT/MEP/Rh family, it is likely that they share a common transport mechanism: it is likely that both electrogenic and electroneutral ammonium transporters bind NH${}_{4}^{+}$ and translocate NH_3_. In this model, electrogenic ammonium transporters would transport H^+^ as a coupling ion in addition to NH_3_.

In conclusion, we have shown that four AMT homologs from a basal land plant, *M. polymorpha*, function as electrogenic ammonium transporters when expressed in *Xenopus* oocytes. Therefore, it is likely that all land plant AMTs are electrogenic and that electrogenic transport by AMTs evolved prior to colonization of land by plants.

## Author contributions

TM performed the phylogenetic analyses, JW performed the electrophysiological analyses and both authors wrote the manuscript.

### Conflict of interest statement

The authors declare that the research was conducted in the absence of any commercial or financial relationships that could be construed as a potential conflict of interest.

## Supplementary material

Supplementary Figure 1 **Phylogenetic analysis of ammonium transporter proteins**. Maximum likelihood tree of AMT/MEP/Rh proteins. Thickened branches represent greater that 70% bootstrap support. The genome of each organism represented was searched for AMT/MEP/Rh genes, all full-length genes were aligned and then translated into amino acid sequences. Where possible, published protein names are used, otherwise each protein from a given organism is numbered according to its the placement on the tree. The placements of transporters from the liverwort *Marchantia polymorpha* are highlighted in bold. Transporters from plants and algae are in green type, transporters from fungi are in purple type, and transporters from animals are in red type.

Supplemental File 1 **Accession numbers and protein sequences of AMT/MEP/Rh family members in the phylogenetic analysis**.
