# Supplementary figures and images for: Evolution of Electrogenic Ammonium Transporters (AMTs)
Source: Front Plant Sci. 2016 Mar 31;7:352. doi: 10.3389/fpls.2016.00352 (PMC4814505; doi:10.3389/fpls.2016.00352)

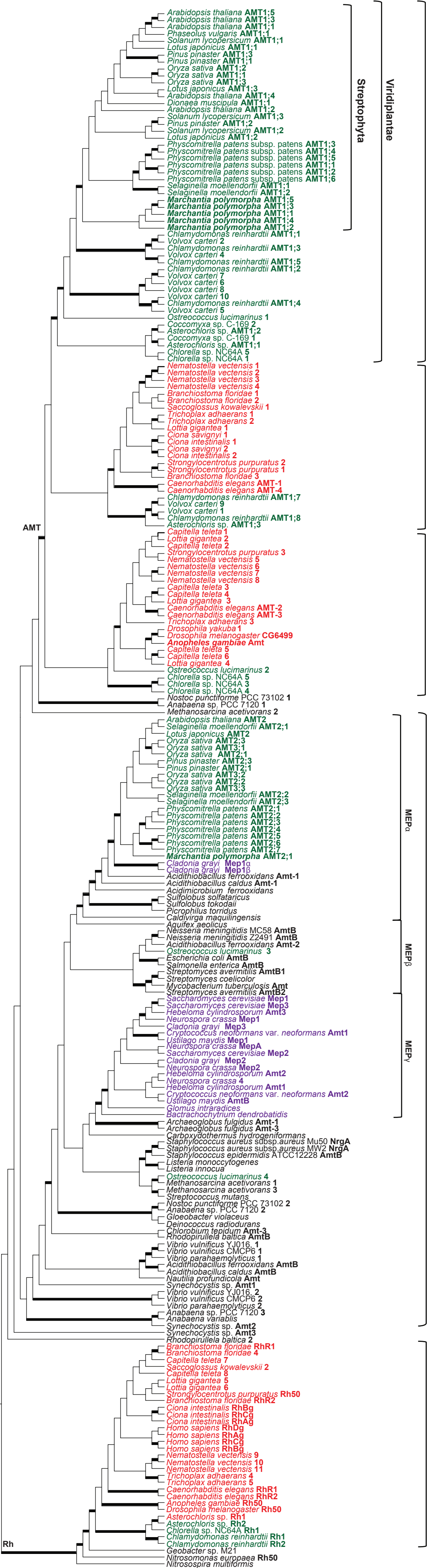

Supplement: Supplementary Figure 1 — Phylogenetic analysis of ammonium transporter proteins. Maximum likelihood tree of AMT/MEP/Rh proteins. Thickened branches represent greater that 70% bootstrap support. The genome of each organism represented was searched for AMT/MEP/Rh genes, all full-length genes were aligned and then translated into amino acid sequences. Where possible, published protein names are used, otherwise each protein from a given organism is numbered according to its the placement on the tree. The placements of transporters from the liverwort Marchantia polymorpha are highlighted in bold. Transporters from plants and algae are in green type, transporters from fungi are in purple type, and transporters from animals are in red type. [file Image1.PDF]
