# Supplementary material for: Evolution of Electrogenic Ammonium Transporters (AMTs)
Source: Front Plant Sci. 2016 Mar 31;7:352. doi: 10.3389/fpls.2016.00352 (PMC4814505; doi:10.3389/fpls.2016.00352)
Supplement: Supplemental File 1 — Accession numbers and protein sequences of AMT/MEP/Rh family members in the phylogenetic analysis. [file Table1.DOCX]

**Supplemental file: Sequences used in alignments. Protein sequences used in the alignment for the phylogenetic tree are shown here. Sequences come from the protein database at NCBI unless otherwise noted as follows: *Solanum lycopersicum* (EMBL); *Asterochloris* sp., *Chlorella* sp., *Volvox carteri* (JGI); Bactrachochytrium (Broad Institute); *Ciona savignyi* (Huang and Peng, 2005).**

1. **Ammonium transporter sequences**

**Viridiplantae (Land plants and green algae)**

*Arabidopsis thaliana* AMT1;1

>gi|15236300|ref|NP_193087.1| ammonium transporter 1;1 [Arabidopsis thaliana]

MSCSATDLAVLLGPNATAAANYICGQLGDVNNKFIDTAFAIDNTYLLFSAYLVFSMQLGFAMLCAGSVRAKNTMNIMLTNVLDAAAGGLFYYLFGYAFAFGSPSNGFIGKHYFGLKDIPTASADYSNFLYQWAFAIAAAGITSGSIAERTQFVAYLIYSSFLTGFVYPVVSHWFWSVDGWASPFRTDGDLLFSTGAIDFAGSGVVHMVGGIAGLWGALIEGPRLGRFDNGGRAIALRGHSASLVVLGTFLLWFGWYGFNPGSFNKILVTYETGTYNGQWSAVGRTAVTTTLAGCTAALTTLFGKRLLSGHWNVTDVCNGLLGGFAAITGGCSVVEPWAAIICGFVAALVLLGCNKLAEKLKYDDPLEAAQLHGGCGAWGLIFTALFAQEKYLNQIYGNKPGRPHGLFMGGGGKLLGAQLIQIIVITGWVSATMGTLFFILKKMKLLRISSEDEMAGMDMTRHGGFAYMYFDDDESHKAIQLRRVEPRSPSPSGANTTPTPV

*Arabidopsis thaliana* AMT1;2

>gi|15217741|ref|NP_176658.1| ammonium transporter 1;2 [Arabidopsis thaliana]

MDTATTTCSAVDLSALLSSSSNSTSSLAAATFLCSQISNISNKLSDTTYAVDNTYLLFSAYLVFAMQLGFAMLCAGSVRAKNTMNIMLTNVLDAAAGAISYYLFGFAFAFGTPSNGFIGRHHSFFALSSYPERPGSDFSFFLYQWAFAIAAAGITSGSIAERTQFVAYLIYSTFLTGFVYPTVSHWFWSSDGWASASRSDNNLLFGSGAIDFAGSGVVHMVGGIAGLCGALVEGPRIGRFDRSGRSVALRGHSASLVVLGTFLLWFGWYGFNPGSFLTILKGYDKSRPYYGQWSAVGRTAVTTTLSGCTAALTTLFSKRLLAGHWNVIDVCNGLLGGFAAITSGCAVVEPWAAIVCGFVASWVLIGFNLLAKKLKYDDPLEAAQLHGGCGAWGLIFTGLFARKEYVNEIYSGDRPYGLFMGGGGKLLAAQIVQIIVIVGWVTVTMGPLFYGLHKMNLLRISAEDEMAGMDMTRHGGFAYAYNDEDDVSTKPWGHFAGRVEPTSRSSTPTPTLTV

*Arabidopsis thaliana* AMT1;3

>gi|15230092|ref|NP_189073.1| ammonium transporter 1;3 [Arabidopsis thaliana]

MSGAITCSAADLATLLGPNATAAADYICGQLGTVNNKFTDAAFAIDNTYLLFSAYLVFAMQLGFAMLCAGSVRAKNTMNIMLTNVLDAAAGGLFYYLFGYAFAFGGSSEGFIGRHNFALRDFPTPTADYSFFLYQWAFAIAAAGITSGSIAERTQFVAYLIYSSFLTGFVYPVVSHWFWSPDGWASPFRSADDRLFSTGAIDFAGSGVVHMVGGIAGLWGALIEGPRRGRFEKGGRAIALRGHSASLVVLGTFLLWFGWYGFNPGSFTKILVPYNSGSNYGQWSGIGRTAVNTTLSGCTAALTTLFGKRLLSGHWNVTDVCNGLLGGFAAITAGCSVVEPWAAIVCGFMASVVLIGCNKLAELVQYDDPLEAAQLHGGCGAWGLIFVGLFAKEKYLNEVYGATPGRPYGLFMGGGGKLLGAQLVQILVIVGWVSATMGTLFFILKRLNLLRISEQHEMQGMDMTRHGGFAYIYHDNDDESHRVDPGSPFPRSATPPRV

*Arabidopsis thaliana* AMT1;4

>gi|15235398|ref|NP_194599.1| ammonium transporter 1;4 [Arabidopsis thaliana]

MASALSCSASDLIPLLSGGANATAAAAAAEYICGRFDTVAGKFTDAAYAIDNTYLLFSAYLVFAMQLGFAMLCAGSVRAKNTMNIMLTNVIDAAAGGLFYYLFGFAFAFGSPSNGFIGKHFFGMYDFPQPTFDYPYFLYQWTFAIAAAGITSGSIAERTQFVAYLIYSSFLTGLVYPIVSHWFWSSDGWASPARSENLLFQSGVIDFAGSGVVHMVGGIAGLWGALIEGPRIGRFGVGGKPVTLRGHSATLVVLGTFLLWFGWYGFNPGSFATIFKAYGETPGSSFYGQWSAVGRTAVTTTLAGCTAALTTLFGKRLIDGYWNVTDVCNGLLGGFAAITSGCSVVEPWAALVCGFVAAWVLMGCNRLAEKLQFDDPLEAAQLHGGCGAWGIIFTGLFAEKRYIAEIFGGDPNRPFGLLMGGGGRLLAAHVVQILVITGWVSVTMGTLFFILHKLKLLRIPAEDEIAGVDPTSHGGLAYMYTEDEIRNGIMVRRVGGDNDPNVGV

*Arabidopsis thaliana* AMT1;5

>gi|15230090|ref|NP_189072.1| ammonium transporter 1;5 [Arabidopsis thaliana]

MSGAITCSAADLSALLGPNATAAADYICGQLGSVNNKFTDAAYAIDNTYLLFSAYLVFAMQLGFAMLCAGSVRAKNTMNIMLTNVLDAAAGGLFYYLFGYAFAFGESSDGFIGRHNFGLQNFPTLTSDYSFFLYQWAFAIAAAGITSGSIAERTKFVAYLIYSSFLTGFVYPVVSHWFWSPDGWASPFRSEDRLFGTGAIDFAGSGVVHMVGGIAGLWGALIEGPRIGRFPDGGHAIALRGHSASLVVLGTFLLWFGWYGFNPGSFTKILIPYNSGSNYGQWSGIGRTAVTTTLSGCTAALTTLFGKRLLSGHWNVTDVCNGLLGGFAAITAGCSVVDPWAAIVCGFVASLVLIGCNKLAELLKYDDPLEAAQLHGGCGAWGLIFVGLFAKEKYINEVYGASPGRHYGLFMGGGGKLLGAQLVQIIVIVGWVSATMGTLFFILKKLNLLRISEQHEMRGMDLAGHGGFAYIYHDNDDDSIGVPGSPVPRAPNPPAV

*Arabidopsis thaliana* AMT2

>gi|15224479|ref|NP_181363.1| ammonium transporter 2 [Arabidopsis thaliana]

MAGAYDPSLPEVPEWLNKGDNAWQLTAATLVGLQSMPGLVILYASIVKKKWAVNSAFMALYAFAAVLLCWVLLCYKMAFGEELLPFWGKGGPAFDQGYLKGQAKIPNSNVAAPYFPMATLVYFQFTFAAITTILVAGSVLGRMNIKAWMAFVPLWLIFSYTVGAYSIWGGGFLYQWGVIDYSGGYVIHLSSGVAGFVAAYWVGPRPKADRERFPPNNVLLMLAGAGLLWMGWSGFNGGAPYAANLTSSIAVLNTNLSAATSLLVWTTLDVIFFGKPSVIGAIQGMVTGLAGVTPGAGLIQTWAAIIIGVVSGTAPWASMMIIHKKSALLQKVDDTLAVFYTHAVAGLLGGIMTGLFAHPDLCVLVLPLPATRGAFYGGNGGKQLLKQLAGAAFIAVWNVVSTTIILLAIRVFIPLRMAEEELGIGDDAAHGEEAYALWGDGEKFDATRHVQQFERDQEAAHPSYVHGARGVTIVL

*Asterochloris* sp. AMT1;1

>gi|338808459|gb|AEJ07738.1| ammonium transporter 1.1 [Asterochloris sp. Armaleo 7/29/2003]

MPPSPAPPPTGPITVAEVVTAFSAASPELPGAFYEANEGFTMISAYLVFFMHCGFAMISIGCVRQKFAKHIAILILLDACASALGFYFFGYAFGFGDRIDANGGYYGNPFVGSQYFALSGLPPTQYYLWFFQWTFAATACTIVSGAIAERTRFEAYACYSFFMSAWVYPIITHSAWSFQGWASMFKTTAHSTNYLFGSGVIDFAGSGAVHMVGGTAALWGAVIVGPRIGRFLSDGTVVQMDGHNSSLFVLGVMILWFGWYGFNPGSQQAIITANGGTWSVASSVANAAVTTTLSPAAAGLSGLLVKAMLLKIETGTLVWDIMTMGNAALAGLVAITSGCSVILPWGGIVVGIVAGSLYVGASKLVVFMKIDDPLDAIAVHLFNGAWGVIAVGFLAGEHQILASYGTNVFTGGNREYGCFLGGSGRLLAAQLVYTCWIFAWVTCMMVPLWLLLKIVGLLRVDPAHETAGLDVSHHGGSAYPGGTDLESSDHKGQQYNTGMNGNGNHHDLTDIRAEIAQLRKQLNAESTPQNPKGTTVVSSGTR

*Asterochloris* sp. AMT1;2

>gi|338808072|gb|AEJ07736.1| ammonium transporter 1.2 [Asterochloris sp. Armaleo 7/29/2003]

MSMVDITSPAFEEAVAEIVEAKTYNWIGDINIEFVLASGYLVFIMQLGFALLTIGAVRAKSAKSVALKNVMDVCAGGIGYYIFGWAFAYGDSERCCPAGNPFIGSQMFALSGMPSTSYSTFYFQFVFAISTATIVSGAVAERVRFMAYGMYALFLTAWVYPVLSHWVWSPTGWASTTRADGPLLFGSGAYDTVGSGAVHMVGGVAGLAGAIVAGPRIGRFTADGKPVDMPGHNTIFYMMGVLLLWLGFYGFNPGTMGQIIATDGTDFAIVVARCAISTTMGACFGGCTALVVSLIYYRIKEGKIIWDLGPCCNGALTGMVVITSGCATYEPWAAAIGGIIGGLVVVPGGWFMLHVCKVDDPVDAFTVHGMGGALGVWWYSLAAKEGFVHELYGATNPDGVTPRHYGWWMGDDATILGANTVWILVIFGWTMGMMLPFFYGLKLCGWLRVSAEEELAGVDVSKHGGHAYPGMEKVYDLEVATNGMGDTLPSHKMRDSDDSNKGAPNDLQLRVRDLEAQLKQVLAHQSNSTEQTPLNTTVV

*Asterochloris* sp. AMT1;3

>gi|338808074|gb|AEJ07737.1| ammonium transporter 1.3 [Asterochloris sp. Armaleo 7/29/2003]

MQAVTNLSDGDITAVLLCSFIVFFQNIGFAALENGNVRAKNARAILLKNCIDKLVGGLCYWAVGFAFAYGSSSNGFIGTSHFFLSQLNGPVAALWVLSATFLMTATTIVSGCLAERAQFEAYLAYTPLMAVLVYPLVVHWVTQGWLTTFATHCTYLDFAGGSMVHLVGGVAGLVAAWQVGPRLGRFDGAIVKPIPGHNVESTVLGTLMLWFGWYGFNIGELLHFIVSVQAGDERGTVAARAAVNTTICGCAGALTALLLAWLRTRTYEVRTCCNGVLGGMVAITALAPFVDPWAAFVLGCIAGGVYIGCSRALLCLRIDDPLDASAVHAGCSLVGLLGVGVFGEPTYIAQNHEGYDRVCGGFFFASPARRGGEQLGVQLLGIITISAFTAACSLALFGTLRYFNKLRVDTATEIAGLDFVDHGGSAYPDFLLRVEAQD

*Chlamydomonas reinhardtii* AMT1;1

>gi|159478831|ref|XP_001697504.1| ammonium transporter [Chlamydomonas reinhardtii]

MSGDFGSEPLGSCSVETVTALLGYGLEQDSITALCQPEGGAGCTSTDNCMFQYLMGATADAQSTASDVGVGLDVSFLLFSGYLVFVMQLGFAVLCAGSIRSKNCMNILLKNMLDACVGAIGFYVFGYAFAYGRKYGQNSNGFIGNWNFALSYTTQTSMSGTEFTTFGWHQFFFQWSFCAATTTIVSGAVAERCTFMAYMIYAFFLSSFVYPVIVHWVWDGQGWLSAFNTFQDGYALILKTGAIDFAGSGVVHMTGGIAALMGAWIMGPRVGRFANDGTVNEMRGHSSTLVVMGTFLLWFGWFGFNPGSNLVVASQAAATVVSRVAVTTALAGGAGGISMLFYKFLTVKAWDVVATCNGILAGLVAVTASCSVIEPWAAIITGAIGAIIFSIADYVTLYKLKVDDPVSAFALHGAVGAWGVLFPGFLAAPHYVVEVYGAYGFGMDAREGKRFGLFYGGHGQVLLVQLIEVLAIFGWTGFMMGSFFFILNKAGLLRVPLQEEMAGLDAANYSKSVGSKDPSVHCTVGVDKLEGGALGEGKA

*Chlamydomonas reinhardtii* AMT1;2

>gi|159469031|ref|XP_001692671.1| ammonium transporter [Chlamydomonas reinhardtii]

MSEACLLAAKSALVGVANGDSLASAICAAMEAAAPVARHLQETTVDLTTLQETVDTIGLNLQGTMDGLNTLLLLYGGALVFLMHGGFAMLEAGAIRSKNAMNILLQTVLDASCSALMWYFVGFAFAFGIGDKPNKFIGNAMFALVDIDTHNTGSGTGKWIDWFFQWAFAATAVTIPAGAVAERFNFNAYLGYSMFIGGWVYPIIAHWVWCIEGWLGYGVIKPFINAGMIDFAGSGVIHMTGGLAGLIGAIMVGPRLGRFDADGKPVDMPGHSAILVVLGTVLLWFGWYGFNPASVLLINSSTYAIVCGRAAVCTTLAGAAGGVSCLIFGFARHRGWDLVGLCNGILCGFVAVTACPHVIYPWAAIICGLVAGLWFEFLCWLLLKLKIDDPLSAGPMHFGCGMWGVFFTGLFARQQYIQEYYVHGPSAAGELSKPYPWGAFYKGYNADGELVMSDGKLLASQIVGILVIIGWVIGMMVPFFGIFKFFGALRIPPEEEEMGLDRSKHGGSAYNGTGANTLGGLSPGNDVMRNNSPLGKVLPVTA

*Chlamydomonas reinhardtii* AMT1;3

>gi|159487104|ref|XP_001701575.1| ammonium transporter [Chlamydomonas reinhardtii]

MASPAPSGSPPLVIYSCTDEQMLEVTELVGDSATAAIVCSQYDCSFTAVSCVLKYLAAQQADLSEQMDGTAKAEVALSLDVAFLLFSAYLVFGPMQLGFALLCAGAIRSKNSMNVLMKNILDACTGAIGFYLFGYAFAFGHHANQTSNAFIGDHNFALSYTTQVSSLDSNVSYDGFATQGWHVFFFQWSFCAVGGAGGASAIVVSRVAVTTTLSAGAAGLTGLFWRYMRTSTWDTVLSRDGYALLLQTGAIDFAGGGVVHLTGGMAALMGAWIIGPRIGRFDASGKVNEMKGHSATLVVMGTFLLWLGFYGFNPGSNLTIATTGCATAAWPAWWA

*Chlamydomonas reinhardtii* AMT1;4

>gi|159470639|ref|XP_001693464.1| ammonium transporter [Chlamydomonas reinhardtii]

MADEMDPMTACITALTDAAMTTAQATALCGQFAFAADNSDLSDRLDQTNQGLNTLYLVSCGALVFVMHAGFAMLCAGAIRSKNTMNILLQTVMDAAVSAVAFYILGYGFAYGIGNNPNGFIGDSLFGLSRWVSHSSSSDAGANWQAWFFQWAFAATATTIPAGAVAERLNFNAYLIYSFFISAFVYPVVVHWVWAAEGWLGYARFGGYSHLFRSGMIDFAGSGVVHMTGGLAGLAGCIMVGPRMGRFDSNGQPVEMPGHSATLVVLGTVLLWFGWYGFNPGSQLIINYTASAAVVGRAAVTTTLSGAAGCLSCLLTAFLRHKAWDLVSGCNGALVGFVSITASANVVEPWAALIAGLVGGWVFDAVCLLFLKLRIDDPLSAAPMHAFCGAWGVFFAGLLAKKEYICESYGRDCEGYVADGLFYVGDGRLLASQVIGIISIFAWVFGLMLLLFGGLKAVGLLRISAEEEQAGLDVSKHGGSAYNYDHGLGKPEKAQALGL

*Chlamydomonas reinhardtii* AMT1;5

>gi|159474182|ref|XP_001695208.1| ammonium transporter [Chlamydomonas reinhardtii]

MGCNPLQLAQVTALLGGDTATAATICAGTDVAQLGPNGAVTKWTVAELRGAQNALDQVEYGLNVSFVLASAYQVFVMQLGFALFAAGVVRPKNTVSIFIKNFFDTCIAGIAFYLVGYAFAFGAKDGYTNGFIGNWDFALSQTDGSPGSEPPNPWHLFIWNWSFCSASTTILSGSIAERATFASYTIYAAFMPAWVYPVVAHWLWAPDGWLSARNANDRILGIGAIDYAGSGVVHLVGGMAALIGSIAVGPRVGRFDAPGSTGEVSAQLYRATAAPQLYLMGTLLLWFGWYGFNPGSKLAISDYLQATIVSRTAVTTTLAACSGALSNLLLNYGRHRVWDLLSTCIGALAGLVAVTSGCSVIEPWAAIICGAISALFYEAGEQLLEKLKVDDPVSAFPLHAMCGVWGLLFTGLLANEGYITQVYNVPPGGHRMGLFYGGHAQLLLCQVIAIAVIAGWSAFNMCILFFGMKAAGLLRVGADKEATGMDMAACGQYNAAGAVNVHSSKVPTKSDAVLAAMSLNMERRMNGGPGMDYSVRNSSANPSAHNSSLHNTATATTVTTAAATPAAAGGIMLTSAVGGASSAGGSAVPSAAASAGVGDAAGSGDTVPPQ

*Chlamydomonas reinhardtii* AMT1;7

>gi|159483301|ref|XP_001699699.1| ammonium transporter, splicing variant [Chlamydomonas reinhardtii]

MQIGFVSAESGQGRAKNVRNILLKNSVNIMLCAICWWAVGYAFAYGNSGGGFIGTSRFFSDGESFGSKPWFFTWTFCLSCVTIASGCLAERTHLFVYPVYTAVVSIAVHPVIAHWVWAPDSWLNSMTGSPCRFLDFAGGAVVHTVGGLTGLIGAMLCGPRLGRFEDGVGKDIPGHDVSSVSLGSLMLWFGWYGFNCGSTYIYMAQGGSEASAAVSRVALNMTLCASVAGMTSLVVSSIQTGTFDLAVCCNGLMAGLSASTSNVGFLTPWAACITGALAGLLYVGISRLLVRMGVDDPLDSSAIHCGSGILGVLVSGFLARPSYVMQMVGCNCGGVVYATNGGMQLGMQLLGLSCSMVWTAMWALLTFGILKKLQLLRVDQQTELAGIDNMEHGGPAYPEFLQRVQSTRPGY

*Chlamydomonas reinhardtii* AMT1;8

>gi|159483299|ref|XP_001699698.1| ammonium transporter, splicing variant [Chlamydomonas reinhardtii]

MPLAELWRLTKLVPLACTVLLLSVRALAVDSGGLNDSADNSSRSLLQESGQASGTSQPYTYSSSDFVLLNGFLIFFMQIGFVSAESGQGRAKNVRNILLKNSVNIMLCAICWWAVGYAFAYGNSGGGFIGTSRFFSDGESFGSKPWFFTWTFCLSCVTIASGCLAERTHLFVYPVYTAVVSIAVHPVIAHWVWAPDSWLNSMTGSPCRFLDFAGGAVVHTVGGLTGLIGAMLCGPRLGRFEDGVGKDIPGHDVSSVSLGSLMLWFGWYGFNCGSTYIYMAQGGSEASAAVSRVALNMTLCASVAGMTSLVVSSIQTGTFDLAVCCNGLMAGLSASTSNVGFLTPWAACITGALAGLLYVGISRLLVRMGVDDPLDSSAIHCGSGILGVLVSGFLARPSYVMQMVGCNCGGVVYATNGGMQLGMQLLGLSCSMVWTAMWALLTFGILKKLQLLRVDQQTELAGIDNMEHGGPAYPEFLQRVQSTRPGY

Chlorella sp. NC64A, 1

>Chlorella sp. NC64A, 1 (JGI 56592)

MAEESVPLTFEDQFAEAFAPATADINATYVIISGVFVFLMQAGFCMLTAGSVRARNARGIILKNLMDTCVAALVYFLLGWGLAGLLIGDGNSFIGTSQFALGGLDPTMLYNWFFQFCFAAAACTIVSGALAERATFVSYILYSVLIIGFVYPVVTHWVWSPSGWASAFRSEQADGTFRSLLFGCGAYDFAGSGAVHMVGGVAAFAGAALLGPRMGRFDANGKPVPIPGHNVALAVLGTFILWFGWYGFNPGSTLMVMNGTSAIVARVCVTTTLGGAAGGIMTLFTHVLIQYKTTGKVVWDVMVAANGALAGLVAITAPCATVEPWAAIIIGMCGAWVYLGGSYLNSHILKIDDPVDAIAVHGWAGIWGVLANGLFATEGFVAQTYGTMPGTEDGVRLYGGFYGGGGKLFAAQLVYVLAIIGWVGGLMGMFFFALKMAGLFRVSAEVEAEGCDTSHYGGSVYHGLTPETGAGATAPASKGVFPSKGDAASSSSDSDELERLKAEVADLSSKVDALAKAGTSTV

Chlorella sp. NC64A, 2

>Chlorella sp. NC64A, 2 (JGI 136742)

MEDFSRQAAAALAGPAGGQQLAAGAEGVAAAAAAGGRTLLQGTLALNTTNATSLMDITVHPFDLGGYLDQQLTMLWVLQCSFLVFFMQCGFALLEAGTVRVKNTKNILLKNVIDACVSTMSWWAVGYAFAYGQCGENGFIGYHDFFSASAGSNSQPTYWTFWLFGWAFSATAATVVSGSMAERTKFRAYLLYTTCISAFIYPVVVHWVWSPSGWLSAHRRPDCGSDQKVPLISGTMGLMDFAGSGVVHMVGGSAALVGATMLGPRLGRFTKDGHVIQFDNSSPANMALGVFILWLGWYGFNAGSTQCFYGCMSVAALVAVNTTLATGAGGLTCLFLAVLNGNPGDIGPLLNGILAGAVSITAGCALVQSYAAVIIGAIGALIYTTFTKVLLKFQIDDPVEAAPVHFFCGAWGLLSVGFFATQTSTEMAYGYADDWGVFYGGSGKQLGMQVLGIVVIAAWSCGLSGGMFLVLKKLNWLRADKDAEQQGLDLSQGIGSGLRGNCFPCLPCCNDLG

Chlorella sp. NC64A, 3

>Chlorella sp. NC64A, 3 (JGI 36096)

MADVAPVLTEAMAAEVNALIEANVGTTLVADVNNYFTVFSGYLVFFMQAGFAMLCAGSVRSKNAKNIILLNILDACFGCCAWYLTGFAFAFGDPVPFEHCGGLEGPALLECEAGDPIPGLSASQAFIGNRFFAQSNLPRSSFWLWFFQFTFAATGATIVSGAVAERCKFEAYMLYELMIVMFVYPVVAHWMWSTCGWLSPWRSEATAAKQSYLYFAGSGVYDFAGDAAVHMVGGVASLAAAWVLGPRIGRFDAAGNPVDMPGHNASLTLLGVFLLWFGWYGFNPGSAVAFSLTGSENYSKVAIAVAINTTVGAAAGTICTLFIAMAYQYFTLGVVVWDLIIAGNGALAGLVSITGPCGFVNTWAAFIIGGIGGFVYFVASKVNLNVLKIDDPLDAIAVHAGCGVWGLLAGGAFSAPGMVTDVYGTMPDGSQRPYGFIMGGNGSVLAANSMAILVVFGWTLAIMTPFFILLKKVGLFRVSPEVEAQGLDVSHHGEFSAL

Chlorella sp. NC64A, 4

>Chlorella sp. NC64A, 4 (JGI 141357)

MASVAGRALAQADPAATMGGDVAAQFTNLEAELTSLWVLVCSFFVFQMQSGFALLEAGTVRAKNTKNILMKNAIDACVATVCWWSVGDAFAYGKCGQNGFIGAYNFFSSEASATGGTYWAFWLWNWAFSATAATIVSGAIAERLQFKAYLIYTTAISGFIYPVVVHWVWSNSGWLSARRRGCDQAEYDPLISGTMGLMDFAGSGVVHMVGGGAALMASIITGPRLGRFDDRGGKPGDFVPSNPVYQALGTFILWLGWYGFNSGSTNCFENCMGVAAQTAVNTTLAASFSALTCLVCAIVMGLPGDIGPVLNGVLAGAVSITAGCALVQSYGAAIIGIIGGLIYTASSRLLVRLKIDDPVDAAPVHFFCGAWGVVAVGFFATETSVQAAYTYAAGWGVFYGGNAKQLGVQVLGVVVIAAWACSLAATLFLVMKKMCWLRVPEHVERQGLDLAQGLSTGIMGKCFKRSAYTMAVESHLVRPSAKPSLSQVQEHEKAGGNGAY-

Chlorella sp. NC64A, 5

>Chlorella sp. NC64A, 5 (JGI 58614)

MSSTVGSGVPAGPSTGNSTAAAATADATSLELALTNFWLLICAALVFFQQCGFALLEAGSVRQKNVRNILLKNTIDASVAVAMWFAVGAAFEGSGVCSGNAFIGTTGFFLAGDVTSRPGYLSRWFFGWAFSATASTTVSGAVAERLRFRSYVMYTLAITGLVYPVAAHWVWSPVGWLSPTRVDCATGERVYTFSNAVGLIDFSGSGVVHLLGGTAALLGAWLLGPRQGRYTADGTLVEMVGCNPSHAALGTFILWFGWYGFNFGSTKCFNEACMELASKIAVNTTLGGGAGGLITLTLTILMGSPGDIGPLLNGILAGLVSVTGPCAVVTPWAAFLIGAIGGTVYTFCSKFFQTKASRTAGGRLWLPRCTSEYCCAWLGPRSASWARIDDPLDVSSVHGCCGAWGLIAGGLFATKTGVQEAYNSDAGYGLFYGGNVAQLGIQLLGLVCIAAWSAAICGSLFFALKKIGWLRASKEVEQRGLDYAQSVGAGTAFWFLHGGRPEQNGSIDQSLSSSTL

Coccomyxa sp. C-169, 1

>Coccomyxa sp. C-169, 1 (JGI 47532)

MAEVAAPAPAPSPFATEDFVTSAITDVSTGGPYIDTNTQYLLSSAYLVFFMHCGFAMISIGCVRARFARHIAVLILIDACASALGFYLFGFAFAFGDKTDADGNVAGNPFIGSQYFALHGLDGPTYFATAVRPYAFWVFEWAFAATACTIVSGAIAERARVEAYAIYSFFMAAWVYPVIVHSVWSSAGWASMFRTAPNFLGYFAKGSGAIDFAGSGAVHMVGGYAAAAGCWIIGPRIGRFNADGTANDFAGHNSSLFVLGVMILWFGWYGFNPGSQLVLVGGTNSFAVSVCAVTTTLAPAAAGLSSLLTKAIVTHFTTGKHVYDVGVMGNGALAGLVAITSGTSTVYPWAALIIGGIAGSLYVFASWVSIKLKLDDPLDAIAVHGWNGTWGVLAVGFFASETLITNSYGLNQDGEVRPYGCFFPGGNGALLAAQIAYALWIAGWVLGNMIPFWLLLKYTGLLRATADEEALGLDSSHHGGSAYAGGMEDDKSHTNGAAGVSSSEFAELKAEIAALKKAQAA

Coccomyxa sp. C-169, 2

>Coccomyxa sp. C-169, 2 (JGI 52218)
MAFNATDFLTSEEFTTTVTKSIQDETFGWIPNINAAFVLTDGYFVFIMQLGFAMLTAGSVRAKSAKSVCIKNVMDVMFGGVAYYLLGWAFAYGDKQECDADAVCSSVGNPFIGTEQFAMAGTPSTSFHTFYFQYVFAISTATIVSGAIAERIQFIAYAFYAFFICAWVYPVLSHWCWTASGFASPTRLASLGPLLLGSGVYDTVGSGAVHMMGGVAGLAGAWVAGPRLGRFNPTTGKPQPIHGHNAVYYTIGYLLLALGFFCFNSGTMAQIIAVDGTSFSGVVARCTISTTMGLCFGGITSVLVYLAYSKYTTGHAVWDLFSAGNGSLMGTIVITSGCATFAPWASAVGGIVGGLMYLPSSLFTLHVMKIDDPVDAFVVHGVGGAAGVIFYALFAEKDLVTELYGLLPDGSQRHWGCFMGDNGTVLAANLIWVLLIAGWTLGMMVPFFYILKLAGLLRLSPQDEEDGPDASHHGGSAYPGLEDAYGDYESSAKNGNAYKAVENGKGGSLNNKIASLESELATLSARVKKMDAVEA

*Dionaea muscipula* AMT1;1

>gi|506956845|gb|AGM31799.1| UNVERIFIED: ammonium transporter [Dionaea muscipula]

MSSPYSVCNPTELVPLLGGSANATAAANYLCNGFATVTYAVDNSYLLFSAYLVFAMQLGFAMLCAGSVRAKNTMNIMLTNVLDAAAGGLSYYLFGFAFAFGAPSNGFIGRQFFALKDVPSASNDGTFDYSFYLYQWAFAIAAAGITSGSIAERTQFVAYLIYSSFLTGFVYPIVAHWFWSVDGWASATRADNLLFGSGVIDFAGSGVVHMVGGVAGLWGALIEGPRIGRFDQAGRPVVLRGHSASLVVLGSFLLWFGWYGFNPGSFLTITKAYGAEGGYYGQWSGVGRTAVTTTLSGSTAALTTLFTKRLLVGNWSVLDVCNGLLGGFAAITSGCSVVDPWAAIICGFGASLVLIGFNKLAEKVGYDDPLEAAQLHGGCGAWGLIFTGLFATKKHVREVYGGSLDRPYGLFMGGGGKLLAAQIIEILVIFGWVTCLMAPLFYGLMVSKLLRVSPEDEMAGMDLTRHGGFAYAYYDEDDPLQKPAVLSTKRVEPASHTPSPYINSSPSV

*Lotus japonicus* AMT1;1

>gi|10952510|gb|AAG24944.1| putative ammonium transporter AMT1;1 [Lotus japonicus]

MALPECSAANLAQLIGPNATDAAAVAGFICDQFTAVGQRFSDTAFAVDSTYLLFSAYLVFSMQLGFAMLCAGSVRAKNTMNIMLTNVLDAAAGGLFYYLFGFAFAFGAPSNGFIGRHFFGLKDVPTVAFDYSYFLYQWAFAIAAAGITSGSIAERTQFVAYLIYSSFLTGFVYPVVSHWFWSGDGWASATNTGNLLFGTGVIDFAGSGVVHMVGGVAGLWGALIEGPRIGRFDHAGRAVSLRGHSASLVVLGTFMLWFGWYGFNPGSFTKILSAYDSGNYYGQWSAIGRTAVTTTLAGCTAALTTLFGKRIISGHWNVTDVCNGLLGGFAAITAGCSVVEPWAAIVCGFVAAVVLIACNMLAEKVRYDDPLEAAQLHGGCGAWGIIFTALFAKEEYVNQVYPGKPGRPYGLFMGGGGKLLGAHVIQILVIIGWVSATMGPLFFILNKMKLLRISTEDELAGMDLTRHGGFAYAYEDDESHKPGIQLRKIEPNSSSTPSAES

*Lotus japonicus* AMT1;2

>gi|31322044|gb|AAM95453.1| Ammonium transporter [Lotus japonicus]

MASLSCSATDLAPLLTATTNATATAAATYLCNQLDTISRKLSDTTYAVDNTYLLFSAYLVFAMQLGFAMLCAGSVRAKNTMNIMLTNVLDAAAGGLSYYLFGFAFAFGAPSNGFIGRHFFGLKHYPSPTYDYSFFLYQWAFAIAAAGITSGSIAERTQFVAYLIYSSFLTGFVYPVVSHWLWSSDGWASPTRTTGSLLFGSGAIDFAGSGVVHMVGGIAGLWGAFIEGPRIGRFDRTGRSVALRGHSASLVVLGSFLLWFGWYGFNPGSFLTIAKAYGNNGENGNYYGQWSAIGRTAVTTTLAGCTAALTTLFSKRLLEGHWKVLDVCNGLLGGFAAITSGCSVVEPWPAIVCGFVAAWVLIGLNLVAAKMKYDDPLEAAQLHGGCGAWGVLFTGLFAKGEYVAEVYGSAGRPYGLLMGGGGKLLAAQVIEILVVCGWVTATMGPLFYGLHKTKLLRISEDDETAGMDLTRHGGFAYAYHDDDDVSTKRGVMMSRIGPGSSSPSTMNTPAASAAANDC

*Lotus japonicus* AMT1;3

>gi|46409004|emb|CAE01484.1| high affinity ammonium transporter [Lotus japonicus]

MAAAFTCSAADLHPLLGTGANATAAAEFICSRFGAISNKFTDTAYAVDNTYLLFSAYLVFAMQLGFAMLCAGSVRAKNTMNIMLTNVLDAATGGIFFYIFGFALAFGTPSNGFIGKHFFGLNEFPSPSFDYGFFLYQWAFAIAAAGITSGSIAERTQFVAYLIYSSFLTGLVYPIVAHWFWSSDGWGSPARTENLLFGSGVIDFAGSGVVHLVGAVAGFWGAFIEGPRMGRFDHAGKPVPLRGHSGTLVVLGTFLLWFGWYGFNPGSFITILKSYGESGSGNYYGQWSAIGRTAVTTTLAGCSAALTTLFGKRLQTGHWNVTDVCNGLLGGFAAITSGCSVVDPWAAIICGFVAAWVLIGCNVLAEKFHYDDPLEAAQLHGGCGTWGIIFTALFAKKQYVNEVYAGLPDRPYGLLMGGGWRLLAAHVVQILTIAGWVSVTMGAVFFVLHKLNLLRISPEEEMAGLDMTSHGGLAYEYHEDVGSVQKALKFVFDFYCKGNYYKTLLE

*Lotus japonicus* AMT2

>gi|15799272|gb|AAL08212.1| putative ammonium transporter AMT2 [Lotus japonicus]

MATPTAYQENLPASPEWLNKGDNAWQMTAATLVGLQSMPGLVILYASIVKKKWAVNSAFMALYAFAAVLLCWVLLCYRMAFGDKLFPFWGKGAPALGQKFLINQASVPESTHYFNNGSIETQTQRPFYPMATLVYFQFNFAAITMILLAGSVLGRMNIKAWMAFVPLWLIFSYTVGAFSIWGGGFLYHWGVIDFSGGYVIHLSSGIAGFTAAYWVGPRLKSDRERFPPNNVLLMLAGAGLLWMGWSGFNGGAPYAANIDASIAVLNTNICAATSLLVWTSLDVAFFGKPSVIGAVQGMMTGLVCITPGAGLVQSWAAIVMGILSGSIPWVSMMILHKKSSLLQKVDDTLGVFHTHAVAGLLGGLLTGLLAEPELCRLVLPVSNSRGAFYGGTGGAQFLKQLVAALFVIGWNLVSTTLILLAIQLFIPLRMPDYQLEIGDDAVHGEEAYALWGDGEKYDPTKHGSTIVDETLASAYSVGARGVTVNL

*Oryza sativa* AMT1;1

>gi|15705368|gb|AAL05612.1|AF289477_1 ammonium transporter 1-1 [Oryza sativa]

MATCAADLAPLLGPVAANATDYLCNRFADTTSAVDATYLLFSAYLVFAMQLGFAMLCAGSVRAKNTMNIMLTNVLDAAAGALFYYLFGFAFAFGTPSNGFIGKQFFGLKHMPQTGFDYDFFLFQWAFAIAAAGITSGSIAERTQFVAYLIYSAFLTGFVYPVVSHWIWSADGWASASRTSGPLLFGSGVIDFAGSGVVHMVGGVAGLWGALIEGPRIGRFDHAGRSVALKGHSASLVVLGTFLLWFGWYGFNPGSFTTILKTYGPAGGINGQWSGVGRTAVTTTLAGSVAALTTLFGKRLQTGHWNVVDVCNGLLGGFAAITAGCSVVDPWAAIICGFVSAWVLIGLNALAARLKFDDPLEAAQLHGGCGAWGILFTALFARQKYVEEIYGAGRPYGLFMGGGGKLLAAHVIQILVIFGWVSCTMGPLFYGLKKLGLLRISAEDETSGMDLTRHGGFAYVYHDEDEHDKSGVGGFMLRSAQTRVEPAAAGCLQQQQPSVTNPERTTSQRRKKSRVSLPLRSRSSRHKFDPHI

*Oryza sativa* AMT1;2

>gi|15705370|gb|AAL05613.1|AF289478_1 ammonium transporter 1-2 [Oryza sativa]

MATCLDSLGPLLGGAANSTDAANYICNRFTDTSSAVDATYLLFSAYLVFAMQLGFAMLCAGSVRAKNSMNIMLTNVFDAAAGALFYYLFGFASRRTPSKGFIGKQFFGLKHMPQTGYDYDFFLFQWAFAIAAAGITSGSIAERTRFSAYLIYSAFLTGFVYPVVSHWFWSTDGWASAGRLTGPLLFKSGVIDFAGSGVVHLVGGIAGLWGAFIEGPRIGRFDAAGRTVAMKGHSASLVVLGTFLLWFGWFGFNPGSFTTISKIYGESGTIDGQWSAVGRTAVTTSLAGSVAALNHAVRQEMADGALERDRRLQRSPRRVRAITAGCSVVDPWASVICGFVSAWVLIGCNKLALMLKFDDPLEATQLHGGCGAWGIIFTALFARKEYVELIYGVPGRPYGLFMGGGGRLLAAHIVQILVIVGWVSATMGTLFYVLHRFGLLRVSTSTEMEGMDPSCHGGFGYVDEDEGQRRVRAKSAAETARVEPRKSPEQAAAGQLV

*Oryza sativa* AMT1;3

>gi|15705372|gb|AAL05614.1|AF289479_1 ammonium transporter 1-3 [Oryza sativa]

MATCADTLGPLLGTAAANATDYLCNQFADTTSAVDSTYLLFSAYLVFAMQLGFAMLCAGSVRAKNTMNIMLTNVLDAAAGALFYYLFGFAFAFGAPSNGFIGKHFFGLKQVPQVGFDYSFFLFQWAFAIAAAGITSGSIAERTQFVAYLIYSAFLTGFVYPVVSHWIWSADGWASASRTSGSLLFGSGVIDFAGSGVVHMVAACRTLGRPHRGPPHWRFDHAGRSVALRGHSASLVVLGSFLLWFGWYGFNPGSFLTILKSYGPPGSIHGQWSAVGRTAVTTTLAGSTAALTTLFGKRLQTGHWNVIDVCNGLLGGFAAITAGCSVVDPWAAIICGFVSAWVLIGLNALAARLKFDDPLEAAQLHGGCGAWGVIFTALFARKEYVDQIFGQPGRPYGLFMGGGGRLLGAHIVVILVIAAWVSFTMAPLFLVLNKLGLLRISAEDEMAGMDQTRHGGFAYAYHDDDASGKPDRSFGGFMLKSAHGTQVAAEMGGHV

*Oryza sativa* AMT2;1

>gi|19909968|dbj|BAB87832.1| ammonium transporter [Oryza sativa]

MAAAGAYSASLPAVPDWLNKGDNAWQLTASTLVGIQSMPGLVVLYGSIVKKKWAVNSAFMALYAYASSLLVWVLVGFRMAFGDQLLPFWGKAGVALTQSYLVGRATLPATAHGAIPRTEPFYPEATLVLFQFEFAAITLVLLAGSVLGRMNIKAWMAFTPLWLLLSYTVGAFSLWGGGFLYRWGVIDYSGGYVIHLSSGIAGFTAAYWVGPRLKSDRERFSPNNILLMIAGGGLLWMGWAGFNGGAPYAANIAASVAVLNTNVCAATSLLMWTCLDVIFFRKPSVIGAVQGMMTGLVCITPGAGLVQTWAAVVMGIFAGSVPWFTMMILHKKSALLMKVDDTLAVFHTHAVAGLLGGILTGLLATPELFSLESTVPGLRGAFYGGGIKQIGKQLGGAAFVIAWNLVVTTAILLGIGLFIPLRMPDEQLMIGDDAAHGEEAYALWGDGEKFDATRHDLSRGGGGGDRDGPAGERLSALGARGVTIQL

*Oryza sativa* AMT2;2

>gi|28875523|dbj|BAC65232.1| ammonium transporter [Oryza sativa Japonica Group]

MSGDAFNMSVAYQPSGMAVPEWLNKGDNAWQMISATLVGMQSVPGLVILYGSIVKKKWAVNSAFMALYAFAAVWLCWVTWGYNMSFGHKLLPFWGKARPALGQSFLLAQAVLPQTTQFYKGGGGADAVVETPWVNPLYPMATMVYFQCVFAAITLILLAGSLLGRMNIKAWMLFVPLWLTFSYTVGAFSLWGGGFLFHWGVMDYSGGYVIHLSSGVAGFTAAYWVGPRSTKDRERFPPNNVLLMLTGAGILWMGWAGFNGGDPYSANIDSSLAVLNTNICAATSLLVWTCLDVIFFKKPSVIGAVQGMITGLVCITPGAGLVQGWAAIVMGILSGSIPWFTMMVVHKRSRLLQQVDDTLGVFHTHAVAGFLGGATTGLFAEPVLCSLFLPVTNSRGAFYPGRGGGLQFVRQVAGALFIICWNVVVTSLVCLAVRAVVPLRMPEEELAIGDDAVHGEEAYALWGDGEKYDSTKHGWYSDNNDTHHNNNKAAPSGVTQNV

*Oryza sativa* AMT2;3

>gi|20160650|dbj|BAB89595.1| putative ammonium transporter [Oryza sativa Japonica Group]

MASPTRPGPYMPRPPAVPEWLNTGDNGWQLAAATFVGLQSMPGLVVLYGSIVKKKWAVNSAFMALYAYASTLIVWVLVGFRMAFGDRLLPFWGKAGAALTEGFLVARASVPATAHYGKDGALESPRTEPFYPEASMVLFQFELAAITLVLLAGSLLGRMNIKAWMAFTPLWLLFSYTVCAFSLWGGGFLYQWGVIDYSGGYVIHLSSGIAGFTAAYWVGPRLKSDRERFSPNNILLMIAGGGLLWLGWAGFNGGAPYAPNITASIAVLNTNVSAAASLLTWTCLDVIFFGKPSVIGAVQGMMTGLVCITPGAGLVHTWAAILMGICGGSLPWFSMMILHKRSALLQKVDDTLAVFHTHAVAGLLGGFLTGLFALPDLTAVHTHIPGARGAFYGGGIAQVGKQIAGALFVVVWNVVATTVILLGVGLVVPLRMPDEQLKIGDDAAHGEEAYALWGDGERFDVTRHEGARGGAWGAAVVDEAMDHRLAGMGARGVTIQL

*Oryza sativa* AMT3;1

>gi|115441305|ref|NP_001044932.1| Os01g0870300 [Oryza sativa Japonica Group]

MSGDAFNMSVAYQPSGMAVPEWLNKGDNAWQMISATLVGMQSVPGLVILYGSIVKKKWAVNSAFMALYAFAAVWLCWVTWGYNMSFGHKLLPFWGKARPALGQSFLLAQAVLPQTTQFYKGGGGADAVVETPWVNPLYPMATMVYFQCVFAAITLILLAGSLLGRMNIKAWMLFVPLWLTFSYTVGAFSLWGGGFLFHWGVMDYSGGYVIHLSSGVAGFTAAYWVGPRSTKDRERFPPNNVLLMLTGAGILWMGWAGFNGGDPYSANIDSSLAVLNTNICAATSLLVWTCLDVIFFKKPSVIGAVQGMITGLVCITPGAGLVQGWAAIVMGILSGSIPWFTMMVVHKRSRLLQQVDDTLGVFHTHAVAGFLGGATTGLFAEPVLCSLFLPVTNSRGAFYPGRGGGLQFVRQVAGALFIICWNVVVTSLVCLAVRAVVPLRMPEEELAIGDDAVHGEEAYALWGDGEKYDSTKHGWYSDNNDTHHNNNKAAPSGVTQNV

*Oryza sativa* AMT3;2

>gi|215713454|dbj|BAG94591.1| unnamed protein product [Oryza sativa Japonica Group]

MSSSATVVPLAYQGNTSASVADWLNKGDNAWQLVAATVVGLQSVPGLVVLYGGVVKKKWAVNSAFMALYAFAAVWICWVTWAYNMSFGEKLLPIWGKARPALDQGLLVGRAALPATVHYRADGSVETAAVEPLYPMATVVYFQCVFAAITLILVAGSLLGRMSFLAWMIFVPLWLTFSYTVGAFSLWGGGFLFHWGVIDYCGGYVIHVSAGIAGFTAAYWVGPRAQKDRERFPPNNILFTLTGAGLLWMGWAGFNGGGPYAANSVASMAVLNTNICTAMSLIVWTCLDVIFFKKPSVVGAVQGMITGLVCITPAAGVVQGWAALVMGVLAGSIPWYTMMILHKRSKILQRVDDTLGVFHTHGVAGLLGGLLTGLFAEPTLCNLFLPVADSRGAFYGGAGGAQFGKQIAGGLFVVAWNVAVTSLICLAINLLVPLRMPDDKLEVGDDAVHGEEAYALWGDGEMYDVTKHGSDAAVAPVVV

*Oryza sativa* AMT3;3

>gi|50725757|dbj|BAD33268.1| putative ammonium transporter [Oryza sativa Japonica Group]

MAAGAIPMAYQTTPSSPDWLNKGDNAWQMTSATLVGLQSMPGLVILYGSIVKKKWAINSAFMALYAFAAVWICWVVWAYNMSFGDRLLPFWGKARPALGQSFLVAQSELTATAIRYHNGSAEAPMLKPLYPVATMVYFQCMFASITIIILAGSLLGRMNIKAWMAFVPLWITFSYTVCAFSLWGGGFLFQWGVIDYSGGYVIHLSSGIAGLTAAYWVGPRSASDRERFPPNNILLVLAGAGLLWLGWTGFNGGDPYSANIDSSMAVLNTHICASTSLLVWTILDVFFFGKPSVIGAVQGMITGLVCITPGAGLVQGWAAIVMGILSGSIPWYTMMVLHKKWSFMQRIDDTLGVFHTHAVAGFLGGATTGLFAEPILCSLFLSIPDSKGAFYGGPGGSQFGKQIAGALFVTAWNIVITSIICVIISLILPLRIADQELLIGDDAVHGEEAYAIWAEGELNDMTHHNESTHSGVSVGVTQNV

*Ostreococcus lucimarinus* 1

>gi|145356298|ref|XP_001422370.1| Amt family transporter: ammonium [Ostreococcus lucimarinus CCE9901]

MAVGDDVDINFMLTNTFLVFFMQAGFAMLCAGSVRSKNTKNILIKNVLDACVGAIAWFLFGYGFAFGATTGKKENAFIGSGNFAMKDVTTASELAMYLFQWSFSAAATTIVSGSVAERTKFEAYLGYSFFLTAFVYPVVVHWTWSGGAGWLGTGYTLGDKVADVGMHDFAGSGVVHMVGGFAGLMGAMIVGPRTGRFASDGRVNPMPGHSAPLVVLGTFVLWLGWYGFNPGSAGGVVDNAETVARCAITTTLSAAAGGVTAMLLNYKLYHVWDLIAVCNGVLAGLVSITAGCAVTEPWAALVCGSLGALVIHFSSKLLLKFQIDDPLEAAPMHGFCGAFGVLWVGFMAKKEYAEAQGARAGAFYEGGGKQLGAQIVGILVIIAWTCGTIGPFFMLMKKFNLLRTTVEEETLGLDESKHGGKAYALELVAPEPA

*Ostreococcus lucimarinus* 2

>gi|145348152|ref|XP_001418520.1| Amt family transporter: ammonium [Ostreococcus lucimarinus CCE9901]

MYCATLVFAMSIGFALLEVGSVSIRNTKSVLVKNVLDLCASGVAYYAVGYGLAWGAGEDGFAGRDGFGMRSAKFDSANHATAAGAHANAFFSACFAATSATIVSGAVAERFPFQSYAVLSSVVAGVIFPIVSHWAWAENGWANPVRGGGRVLFDVGVLDYAGSGVVHVTGGLCALWAVYVVGARAGRFSHGRRANDMPQQNPVYQVIGGMLMWYGWFGFNCGSVRTLAEEHLITVARVGLVTALSGCLGGITVVCIDMYRDRSRVRPARMINGILTALVSSSGGAAFVEIGVSLVIGVVAGVLYVWASDFMVRKKLDDVVDAVAVHFFGGIWGIVASALFSAPKFLKHFYGDSYKGCGLLYGCSHGGAVFGAALVFLLALCAWVSVTALAVLAVLKYFNALRVSADRECNGLDRSMHGGSSYTEFQTTIFRFKDKSGSESQMEMRVRAGDAARFAMVLSEIMETDAKPTSVATSRRSSTSDGSLTPSPSQSPGRSSAPLLPGDLGSLGEAKLTITEE

*Ostreococcus lucimarinus* 3

>gi|145348949|ref|XP_001418904.1| Amt family transporter: ammonium [Ostreococcus lucimarinus CCE9901]

MTTTTTTTTRGARLSTDARARASAARASGCARAARANGAEKNSRVLGRAIGATAGRRARVAGAVSVDAARATLAEAESNAARSVFARAKTPVAAAAVAVAGLTLAGPAAAAAAAVPAVVGADTCWILISSALVLFMTIPGLAAFYAGLVRRTNTLSVLIQCFALTAHMSVLWFLCGYSLSFSDVGMKEGVTTLASFIGGWDKVGLAGVTAASVAGTIPEALWVLYQMTFAIITPALMIGALVERMKFNAVMWFSTLWMFAVYFPACHMVWGGPGAFFADMGVLDFAGGIVVHITAGIGALVAAMYLGERKENKMHQGNLVLTFLGTAMLWVGWFGFNGGSAGAASAGAAFACLATQLSASVAAIVWTALDVIENGKVSVLGTCTASIAGLAAITPAAGFVGPVGACLMGLASAIVCRFFSTTVKEKFGYDDALDVFGVHGVGGFLGTILLGVLAHPALGGFNDVPMMKQTIVQVIAAVSTAVYTAGASWACLKLTDKITGGIRVPAEAEELGLDVYSHGETAYTPMPPVR

*Ostreococcus lucimarinus* 4

>gi|145346881|ref|XP_001417910.1| Amt family transporter: ammonium [Ostreococcus lucimarinus CCE9901]

MAIDAGTTAFMIVSMALVNLMTPGLAFFYGGLVRSSNVLTIMMQNYVSMGLITLVWAVWGYSLCFGTSKNGVIGSARTHYFLENLATQELPYAEGMPGLVFAGFQGMFAVIAPSLMTGAFADRVRFVPYVLFIFFWVHLVYFPFAHWVWGGGWMARNGVKDFAGGIVVHITAGFSALATVVALPNRRKLEGDDVDTNPHNIPFVALGTGLLWFGWFGFNGGSALAANESAALAAMNSEISASAALFFWLMLEWKMEGKPTLVGACVGAIAGLATITPCAAYVKPWAALTIGTLCVPWCYACIELTKKYNLDDALDVWAVHGMGGFFGTLCVGIFSSAAMTGNAFEAGGKLFGKQLLAAVGCAAYSFVMGYAIIKVLEKFMDVVPREGDRRMGLDVSMHGAKAYSEDPSGSKKGGAVNV

*Phaseolus vulgaris* AMT1;1

>gi|300119971|gb|ADJ68003.1| AMT1;1 ammonium/proton co-transporter [Phaseolus vulgaris]

MSLQTCSADQLAQLFGSNTTDPHAAATLICDQFAAVSRNFVDTGYAIDSTYLLFSAYLVFSMQLGFAMLCAGSVRAKNTMNIMLTNVLDAAAGGLFYYLFGFAFAFGSPSNGFIGKHYFGLTDIHAQNLDYSNFLYQWAFAIAAAGITSGSIAERTQFVAYLVYSSFLTGFVYPVVSHWFWSPDGWASAFNLNNLLFSTGVIDFAGSGVVHMVGGVAGLWGALIEGPRIGRFDHTGRAVALRGHSASLVVLGTFMLWFGWYGFNPGSFNRILVPYNSGSKYGQWSAVGRTAVTTTLAGSTAALTTLFGKRVLSGHWNVTDVCNGLLGGFAAITAGCAVVEPWAAIVCGFVASVVLIACNKLAERVRFDDPLEAAQLHGGCGAWGVIFTALFAKKEYVFEAYGGDASRPHGLFMGGGGRLLAAHLIQILVIVGWVSATMGPLFWLLKKLNLLRISSEDELAGMDMTRHGGFAYAYEDDETQKHGIQLRKVEPNSSTPTTDE

*Physcomitrella patens* AMT1;1

>gi|168068394|ref|XP_001786055.1| predicted protein [Physcomitrella patens]

MERIQGFLGAVSNATGLSIFLCDKLDNIDGRLGYTKLAVDNTYLLFSAYLVFAMQLGFAMLCAGSVRAKNTMNIMLTNVLDAACGGISYYVFGFAFAFGLGGKTNGFIGHFNWGLNGFPNGTFDYSFFLFQWAFAIAAAGITSGSIAERTQFVAYLVYSSFLTGFVYPIVSHWLWSADGWLSASKTVGPGGLLFGSGAIDFAGSGVVHMVGGVAGFWGALIEGPRIGRFDKSGNSTNFRGHSATLVVLGTFLLWFGWYGFNPGSFLTILQPYEGVKGHWSGVGRTAVTTTLAGCTAAVTTLFGKRFLDGHWNVLDVCNGLLGGFAAITASCSVVAPWASILCGFGSAWVLIGLNKLAARLHFDDPLEAAQLHGGCGAWGLLFVGLFAEKNYVNQVYQTTFDSPYGLFMGGGGKLLAAQIIEIISIAAWVTVTMGPLFYGLHKFRLLRITPQDEIAGMDVTRHGGTAYIHHDNSEHHLQMHTINGQKRTDNDQMPI

*Physcomitrella patens* AMT1;2

>gi|168067175|ref|XP_001785500.1| predicted protein [Physcomitrella patens]

MSIFGCQDGDLATIQGFLGAVPNATGLSSFLCDKLNLIDGRLGSTKLAVDNTYLLFSAYLVFAMQLGFAMLCAGSVRAKNTMNIMLTNVLDAACGGISYYVFGFAFAFGLGGKTNGFIGHFNWGLNGFPNGTFDYSFFLFQWAFAIAAAGITSGSIAERTQFVAYLVYSSFLTGFVYPIVSHWLWSADGWLSASKTVGPGGLLFGSGAIDFAGSGVVHMVGGVAGFWGALIEGPRIGRFDKSGNSTNFRGHSATLVVLGTFLLWFGWYGFNPGSFLTILQPYEGVKGHWSGVGRTAVTTTLAGCTAAVTTLFGKRFLDGHWNVLDVCNGLLGGFAAITASCSVVAPWASILCGFGSAWVLIGLNKLAARLHFDDPLEAAQLHGGCGAWGLLFVGLFAEKNYVNQVYQTTFDRPYGLFMGGGGKLLAAQIIEIISIAAWVTVTMGPLFYGLHKFRLLRITPQDEIAGMDVTRHGGTAYIHHDNSEHHLQMHTINGQKRTDNDQMPI

*Physcomitrella patens* AMT1;3

>gi|168011825|ref|XP_001758603.1| predicted protein [Physcomitrella patens]

MTTYGCAEGDLATIQGFLGAAGNSTGLSEFLCTKLDGIDARLGFTALAVDNTYLLFSTYLVFAMQLGFAMLCAGSVRAKNTMNIMLTNVLDAACGGISYYVFGFAFAFGVGGKANGFIGHANWGLKNVPNASFDYSFFLFQWAFAIAAAGITSGSIAERTQFVAYLVYSSVLTGFVYPIVSHWLWSTDGWLSATKSMGPGGLLFGSGAIDFAGSGVVHMVGGLAGFWGALIEGPRMGRFDKSGNSLNFRGHSATLVVLGTFLLWFGWYGFNPGSFLKILVPYEGVKGNWSGVGRTAVTTTLAGSTAGVTTLFGKRLLDGHWNVLDVCNGVLGGFAAITASCAVVSPWASIICGFGAAWVLIGLNKLAARFHYDDPLEAAQLHAGCGAWGLLFVGLFAEKNYVQQIYGGDPERPYGLFMGGGGKLLAAQIVEILAITGWVSVTMGLLFLGLQKFKLLRISPEDEVAGMDLTRHGGTAYIHQDGSEHIMHMSTINGDKRSLNNQGPI

*Physcomitrella patens* AMT1;4

>gi|168011817|ref|XP_001758599.1| predicted protein [Physcomitrella patens]

MTTYGCAAGDLTILQGYLGASGNATGLSEFLCSKLDGIDARLGFTTLAVDNTYLLFSTYLVFAMQLGFAMLCAGSVRAKNTMNIMLTNVLDAACGGISYYVFGFAFAFGQGGKPNGFIGHANWGLQNVPNASFDYSFFLFQWAFAIAAAGITSGSIAERTQFVAYLVYSSVLTGFVYPIVSHWLWSTDGWLSATKSVGPGGLLFGSGAIDFAGSGVVHMVGGLAGFWGAFIEGPRIGRFDKSGNSMNFRGHSATLVVLGTFLLWFGWYGFNPGSFLKILVPYEGVKGNWSGVGRTAVTTTLAGSTAGVTTLFGKRLLDGHWNVLDVCNGVLGGFAAITASCAVVSPWASIICGFGAAWVLIGFNRLAARFHYDDPLEAAQLHAGCGAWGLLFVGLFAEKNYVQQIYGGNADRPFGLFMGGGGRLLAAQIVQILAITGWVTVTMGPLFLALHRFRLLRISSEDEIAGMDLTRHGGTAYIHNDGSEQLVHFTTKGDKNHNGKIPI

*Physcomitrella patens* AMT1;5

>gi|168011819|ref|XP_001758600.1| predicted protein [Physcomitrella patens]

MTTYGCAAGDLTILQGYLGASGNATGLSEFLCSKLDGIDARLGFTTLAVDNTYLLFSTYLVFAMQLGFAMLCAGSVRAKNTMNIMLTNVLDAACGGISYYVFGFAFAFGQGGKPNGFIGHANWGLQNVPNASFDYSFFLFQWAFAIAAAGITSGSIAERTQFVAYLVYSSVLTGFVYPIVSHWLWSTDGWLSATKSVGPGGLLFGSGAIDFAGSGVVHMVGGLAGFWGAFIEGPRIGRFDKSGNSMNFRGHSATLVVLGTFLLWFGWYGFNPGSFLKILVPYEGVKGNWSGVGRTAVTTTLAGSTAGVTTLFGKRLLDGHWNVLDVCNGVLGGFAAITASCAVVSPWASIICGFGAAWVLIGFNRLAARYHYDDPLEAAQLHAGCGAWGLLFVGLFAEKNYVQQIYGGDSRPFGLFMGGGGRLLAAQIVQILAITGWVTVTMGPLFLALHKFRLLRITPEDEIAGMDLTRHGGTAYIHQDGSEHVMHLSTINGDKRNANDQMPI

*Physcomitrella patens* AMT1;6

>gi|168011841|ref|XP_001758611.1| predicted protein [Physcomitrella patens]

MSTFGCSAEDVTTLQGFLGNTANAAGLSSFLCKKLDDVSSQLDATTLAVDNTYLLFSAYLVFAMQLGFAMLCAGSVRAKNTMNIMLTNVLDAACGGLSYYIFGFAFAFGIGSKTNGFIGHYNWALRGYPNESFDYSYFLFQWAFAIAAAGITSGSIAERTQFVAYLIYSSVLTGFVYPIVSHWLWSTDGWLSASKSVGPGSLLFGSGAIDFAGSGVVHMVGGIAGFWGAFIEGPRIGRFDKTGNPMVFRGHSATLVVLGTFLLWFGWYGFNPGSFVNILVPYSGNRGAWTGVGRTAVTTTLSGCTAAVTTLFAKRLLHNHWSVLDVCNGLLGGFAAITAGCAVVAPWASIICGFVSAWVLIGLNKLAARVHYDDPLEAAQLHAGCGAWGLIFVGLFAQQNYILQIYDGAGDRPHGLFMGGGGKLLAAQIIEILAIFGWVTVTMGPLFFLLKLFNLLRISPEDEMAGLDLTRHGGTAYIHHDGSDHPMHMHSMNGQKRPNEGPGGNYDGTNV

*Physcomitrella patens* AMT2;1

>gi|168004233|ref|XP_001754816.1| predicted protein [Physcomitrella patens]

MATPVVQSAYNQTGGVSPDWLNKGDNAWQLTASTLVGLQSMPGLVILYGSIVKKKWAVNSAFMALYAFAATLLCWVTWAYKMSFGEQLLPIWGKAGTTLGYTFMLDQARLPSTIHYYKNNTIEMPASEGFYGMADMVLYQFFFAAITLVLLAGSVLGRMSFRAWMLFCPLWLTFSYTVGAFSVWGGGFLWQWGVIDYAGGYVIHVSSGVAGFVAAYWVGPRLAKDRERFPPNNVLLMLTGAGLLWMGWSGFNGGAPFSANLISGLAVLNTHICAATSLLTWTTLDVIVFGKPSVIGAVQGMMTGLVMITPAAGFVPGWAALVMGILAGSIPWWTMMCLHKRWSLLQRVDDTLGVFHTHAVAGCLGGLCVGLFAEPRLCEYMNHAVTNSNGLFYGGDGGMQVLKQVVGAAFIIGWNIVVTTLIMFAIRFVMPLRMSEEHLLVGDDAEHGEEAYALWGDGEKFDVTRHIHSDLEMEQSRFGVRQSQTFAPNTILHI

*Physcomitrella patens* AMT2;2

>gi|168058415|ref|XP_001781204.1| predicted protein [Physcomitrella patens]

MIAESTSYVPSAYQNTGGVLPDWLNKGDNAWLLTASTLVGLQSMPGLVILYGSIVKKKWAVNSAFMALYAFAATLLCWVGWAYKMSFGDQLLPLWGKAGTTLRYTYLLSQAGMPSTIHLFKNGTQEASALTLNYGMANMVLFQFFFAAITIILLAGSVLGRMSFRAWMLFVPLWLTFSYTVGAFSVWGGGFLWQWGVIDYAGGYVIHVSSGVAGFVAAYWVGPRLPKDRERFPPNNMLLMLTGAGLLWMGWSGFNGGAPFSANIVSGLAVLNTHVCAATSLLTWTTLDVIIFGKPSVIGAVQGMMTGLVTITPAAGFVPGWAALIMGVLAGSIPWWTMMWLHKRWSLLQNVDDTLGVFHTHAVAGCLGGLCVGLFAEPELCTYMNLPVTNSNGLFYGGNGGVQVLKQIVGALFIIGWNIVVTTLIIFAVGMLMPLRMSEEHLLVGDDAEHGEEAYALWGDGEKFDVSRHARSADSYEMEQSHHGPRPDRNAILHI

*Physcomitrella patens* AMT2;3

>gi|168052164|ref|XP_001778521.1| predicted protein [Physcomitrella patens]

MNTGEVLPDWLNKGDNAWQLTAVTFVGLQSMPGLVILFGSIVKKKWAVNSAFMSLYAFAATLLCWVTWAYKMSFGEWLLPMWGKAGTTLGYEFLLEQAKMPSTVHFYKNGTVESDAVEAMYGMADFVLFQFFFAAITLILLGGSVLGRMSFRAWMLFVPLWLALSYTVGAFSLWGGGFLWQWGVIDYAGGYVIHVSSGVAGFVAAFWVGPRLPKDRERFPPNNMLLMLTGAGLLWMGWSGFNGGAPFSANIISGLAVLNTHVCAATSLLTWTTLDVIFFGKPSVIGAVQGMMTGLVTITPAAGFVPGWAALIMGVLARSVPWWSMMWLHKHWSLLQDVDDTLGVFHTHAVAGCLGGVCVGLLADPTLCAYMDVPVTNSNGLLYGGNGGVQVLKQIVGALFIIGWNIVVTTLIIFAIGMVMPLRMSEEHLLVGDDAEHGEEAYALWGDGEKFDDLSLNTYRR

*Physcomitrella patens* AMT2;4

\>gi|168035110|ref|XP_001770054.1| predicted protein [Physcomitrella patens]

MEAPPPLPPGVTSAYTNSGYGVEPEWLNKGDAAWQLAAATLVGIQSMPGLVILYGSIVKKKWAVNSAFMALYAFAATAICWVLWAYKMSFGEQLVPIWGKAGSVLGFEFLLSQAGLPSSAHYDSNGALVTPALKAHFGMASMVLFQFFFAALTVILLAGSVLGRMSFRAWMLFVPLWLTFSYTVGAFTMWGGGFLWQWGVIDYAGGYVIHVSSGAAGFVAAYWVGPRLPKDRERFPPNNMLLVLTGAGLLWLGWSGFNGGASFSAHVVTGLAVLNTHISAATSMLTWTAMDVFFFGKPSVIGAVQGVMTGLVVITPAAGLVPGWSALLMGILAGSIPWWTMMVLHKRWWVLQKVDDTLGVIHTHGVGGVLGGICVGLFADPTLCKYMHVAGDYTNGAFYGGNGGIQLLKQIVGALFIAGWNLVLTTLILFAIGLVTPLRMSEEHLLVGDDAEHGEEAYALWGDGEKFDVTRHPTATIAAEFEQRSAVNYADRAVTLHI

*Physcomitrella patens* AMT2;5

>gi|167999618|ref|XP_001752514.1| predicted protein [Physcomitrella patens]

MADATPAIPSAYVSTGGVNPDWLNKGDNSWQLTAATFVGMQTVPALVILYGSIVKKKWAVNSAFMAFYAFAAVLLCWVGWAYKMSFGEKLIPIWGKAGTTLNFNYLLSQAELPASATYYSDGSVQVATLMPWYPMATQVYFQFVFAAITLVLLAGSVLGRMSFRAWMVFVPLWLTCSYTVGCFSVWGGGFLWQWGIIDYAGGYVIHLSSGVAGFTAAYWVGPRLSKDRERFPPNNVLLMLAGAGLLWMGWAGFNGGAALSANLVASIAIINTHVCAATSVLVWTFLDVVVFGKPSVIGAVQGMITGLVVITPGAGLVQGWAALVMGVFAGSIPWFTMMVVHKRSSLLQKVDDTLGVFHTHAVAGFLGGILSGCLAEPTLCNYFLPVLDLQGAFYGGIGGKQLGKQIAGALFIIGWNVVVTSIILNVIKLVIPLRMDDEHLLIGDDAEHGEEAYALWGDGEKYDSSVHGASEMDITNHGSKNNDKRPTVAL

*Physcomitrella patens* AMT2;6

>gi|168020545|ref|XP_001762803.1| predicted protein [Physcomitrella patens]

MALTPPAPVTPEWLNKGDNAWQLTASTLVGIQTVPALVILYGSIVKKKWAVNSAFMAFYAFAAVLLCWVAWAYKMSFGEKLIPIWGKAGTTLSFKYLLSQAELPASATPDVPKLMPFYPMATHVYFQFVFAAITLVLLAGSVLGRMSFRAWMLFVPLWLTCSYTVGAFSLWGGGFLWQWGVIDYAGGFVIHLSSGIAGFTAAYWVGPRLTRDRERFPPNNVLLMLAGAGLLWMGWAGFNGGAAVSANLIASIAVINTNVCAATSLLVWTGLDVVVFGKPSVIGAVQGMITGLVVITPGAGLVQGWAALVMGVFAGSVPWFTMMVVHKKSKLLQKVDDTLGVFHTHAVAGLLGGLLSGCLAENTLCDYFLPVVGLQGAFYGGSGGKQLGKQIVAALFIIGWNIVVTSIILNVIKLITPLRMSDEHLLIGDDAEHGEEAYALWGDGEIYDLSKHGASELDNTKHGSMEFDKQPTVAL

*Physcomitrella patens* AMT2;7

>gi|168003074|ref|XP_001754238.1| predicted protein [Physcomitrella patens]

MADALPTVPIAYNQTGGVTPAWLNKGDNAWQLTASTLVGVQSVPALVILYGSIVKKKWAVNSAFMAFYAFAAVWLCWVGWAYKMSFGEKLIPIWGKAGTTLSYKYLLSQAELPSTAHYHKNGDIETYALTPFFPMASLVYFQFVFAAITLVLLAGSVLGRMSFRAWMLFVPLWLTCSYTVGAFSLWGGGFLWQWGVIDYAGGFVIHLSSGIGGFVAAYWVGPRLTKDRERFPPNNVLLMLAGAGLLWMGWAGFNGGAALSANLIASIAVMNTNVCAATSLLVWTCLDVLIFGKPSVIGAVQGMITGLVVITPAAGLVQGWAALVMGVFAGSVPWFTMMVVHKRSSILQRVDDTLGVFHTHAVAGLVGGLLVGCFAEPTLCDYFLPVLGERGAFYGGVGGKQLGKQIVGALFITAWNVVMTSIILNVIKLVMPLRMTDEHLLVGDDAEHGEEAYALWGDGEMFDMSKHGALTSNLSTLSSNDFGSEKSRPTITL

*Pinus pinaster* AMT1;1

>gi|584432016|gb|AHI94951.1| ammonium transporter 1.1 [Pinus pinaster]

MSNFDYSCTAPELAMLQSLLNGTAGDFFCNKLQAVSDRLSATSRAVDSTYLLFSAFLVFSMQLGFAMLCAGSVRAKNTVNIMLTNVLDAAAGGIFYYLFGFAFAFGRGKHSNGFIGHYFFGLTEVPNQEYQYDYAYFLYQWAFAIAAAGITSGSIAERTQFVAYLIYSSILTGFVYPVVSHWVWSPDGWMSASNASGLLFGSGVYDFAGSGVVHMVGGIAGFWGALIEGPRIGRYDKEGKPNGIRGHSATLVVLGFPSLWFGWYGFNPGSFAKILVPYGAGGGSYVDGQWTAIGRTAVTTTLAGCSAALTTLFGRRLLTGHWNVLDVCNGLLGGFAAITGGCSIVDPWAAILCGFVSAWVLIGFNILAGKMKYDDPLEAAQLHGGCGAWGIIFTALFAKEEYILQAYGLLPNGKSSRPSGLFMGGDGRLLAAHVIQIIVTTGWVTATMAPVFYVLHRFKLLRVSAADEMAGMDVTRHGGGAYVYHDSDDDKHIHPGGFMMKASRNMTEFPHNNNYRNGAEDNDDDTL

*Pinus pinaster* AMT1;2

>gi|584432018|gb|AHI94952.1| ammonium transporter 1.2 [Pinus pinaster]

MGGTFESLLMSTVSSGSSINGTLQGDQAAVLDSMTAIKDAINAVYLLFSAYLVFSMQLGFAMLCAGSVRAKNTMNIMLTNVIDAAIGGVFYYLFGFAFAFGTSSGFIGHSFFALTGVPNDTYDYSYYLYQWAFAIAVAGITSGSIAERTQFGAYLTYSCVLTGFVYPIASHWFWSSYGWASPDRNKGLLFGSGAIDFAGSGVVHVVGGVAGLWGALIEGPRVGRFDAFGKSVAMRGHNATLVVLGTFLLWFGWYGFNAGSFIQILTTSTSGLLEGNWSGVGRTAVTTTLAGSMAALTTLFGKRLLSGHWNALDVCNGLLGGFVAITSGCSVVDPWAAIVCGFVAAWVLIGFNALALRLKFDDPLEAAQLHGGCGVWGVIFTGLFAAENHMVEVYPPANGDTTRPFGLLMGGGWRLLGAQVVEVLAIVGWVTVTMGPLFYAMQRMDLLRIGREEETAGMDISSHGGFAYDHSLPDEGPSRRYIDYLQK

*Pinus pinaster* AMT1;3

>gi|584432020|gb|AHI94953.1| ammonium transporter 1.3 [Pinus pinaster]

MSSSSYTCTAPELAALQNLLNGTAGDFFCNKLQAVSDQLSATSRAVDTTYLLFSAFLVFAMQLGFAMLCAGSVRTKNTVNIMLTNVLDAAAGGIFYYLFGFAFAFGRGKHSNGFIGHYFFGLKDVPNENFQYDYAYFLYQWAFAIAAAGITSGSIAERTQFAAYLVYSSILTGFVYPVVSHWVWSPDGWLSASKASGLVFGSGVYDFAGSGVVHMVGGVAGLWGALIEGPRIGRYNHEGKPHAIRGHSATLVVLGTFLLWFGWYGFNPGSFVKILVPYSSGGGSYVDGQWTAIGRTAVTTTLAGCSAALTTLFGRRLITGHWNVLNVCNGLLGGFAAITGGCSMVEPWAAIVCGFVSAWVLIGFNILAAKMKYDDPLEAAQLHGGCGAWGIIFTALFAKEEYVFQVYGLSPDGISSRPYGLFMGGGGRLLAAHIIQILVTAGWVTATMAPAFYILHKFNLLRVSGDDEMAGMDKTSHGGVAYVFHDGDGDGDAQIHPTGFMVKASPSISLTP

*Pinus pinaster* AMT2;1

>gi|584432023|gb|AHI94954.1| ammonium transporter 2.1 [Pinus pinaster]

MATYLPTAYQNGTTSPDWLNKGDNAWQMVAATLVGMQSMPGLVILYGSIVKKKWAVNSAFMALYAFSAVMICWVTWAYKMAFGHKLLPLWGKAGPALGQKYLIGAADLPASQHNHHNGTLETAMIAPFFPMATMVFFQFSFAAITLILLAGSVLGRMNIKAWMAFVPLWLTFSYTVGAFSLWGGGFLFQWGVIDYSGGYVIHVSSGISGFTAAYWVGPRLTKDRERFPPNNVLLMLAGAGLLWMGWSGFNGGDPYSANIDSSMAVLNTNICAATSLLVWTCLDVIFFGKPSVIGAVQGMITGLVCITPAAGVVQGWAAIAMGVLSGIIPWFTMMVLHKRSTLLQKVDDTLGVFHTHAVAGVLGGALTGLFAEPTLCSLFLPVTNSKGAFYRGPGGVQFLKQLAGACFIIGWNVVATSIILLVISLVIPLRMTDEQLLIGDDAIHGEEAYALWGDGEKYDNTKHGWYDDTTAGDGRGQGARGVTIEL

*Pinus pinaster* AMT2;3

>gi|584432025|gb|AHI94955.1| ammonium transporter 2.3 [Pinus pinaster]

MAYQGVNGTASPPWLNKGDNAWQMVAATLVGMQSMPGLVILYGSIVKKKWAVNSAFMALYAFAAVLICWVTWGYKMSFGNQLLPLWGKAGPALDQYYLVGQAAIPAEPATALFPMATMVYFQFVFAAITLILLAGSVLGRMNIKAWMVFVPLWLTFSYTVGAFSLWGGGFLFQWGVIDYSGGYVIHVSAGIAGFTAAYWVGPRLKQDRERFPPNNVLLMLAGAGLLWLGWAGFNGGDPFAANIDSSMAVLNTNICAAASLLVWTCLDVIFFGKPSVIGAVQGMITGLVCITPAAGVVQGWAAIIMGVLSGSIPWFTMMVLHKKSKLLQMVDDTLGVFHTHAVAGVLGGALTGLFAEPVLCDLYLSSPGTKGAFYGGSGGKQFLKQLVGAGFIIGWNVVATSIILLVIRFVIPLRMPDEQLLIGDDAVHGEEAYALWGDGEKYDISKHGWSDDTTGHGDVTGQGARSIIELGKPARGATIEL

*Selaginella moellendorffii* AMT1;1

>gi|302767418|ref|XP_002967129.1| hypothetical protein SELMODRAFT_169094 [Selaginella moellendorffii]

MALAAVASIRCSSQDLLSFGLAANASSALCSKLDFVADRLYATQLAVDNSYLLFSAYLVFAMQLGFAMLCAGSVRAKNTMNIMLTNVLDAACGGISYYLFGFAFAFGTGGTTNGFIGRYFFALSEIPDSSAGFDYSFFLFQWAFAIAAAGITSGSIAERTQFVAYLIYSTFLSGFVYPIVSHWAWSVDGWASASKPSGRLFGSGAIDFAGSGVVHLVGGLAGFWAAQIEGPRIGRFDKGGAATLVLKGHSASLVVLGTFLLWFGWYGFNPGSFVTILSPYGAGSFTGNWTGVGRTAVTTTLAGCSAAITTLFGRRLLTGHWAVVDVCNGLLGGFAAITAGCSVVDPWASLICGFVSAWVLIGLNLLAERFHYDDPLEAAQLHGGCGTWGLIFTALFAKEEHVLNVYGRTSTPYGLFLGGGGRLLAAQIVYILAIVGWVTVTMGPLFWMLHRLNLLRISPEDEVAGMDLTRHGGMAYYHQDGSHHDGHKFQLHNLSKHGGGNAPGMVHDAASPL

*Selaginella moellendorffii* AMT1;2

>gi|302754918|ref|XP_002960883.1| hypothetical protein SELMODRAFT_163770 [Selaginella moellendorffii]

MALAAVASIRCSSQDLLSFGLAPNASSALCSKLDFVADRLYATQLAVDNSYLLFSAYLVFAMQLGFAMLCAGSVRAKNTMNIMLTNVLDAACGGISYYLFGFAFAFGTGGTTNGFIGRYFFALSEIPDSSAGFDYSFFLFQWAFAIAAAGITSGSIAERTQFVAYLIYSTFLSGFVYPIVSHWAWSVDGWASASKPSGRLFGSGAIDFAGSGVVHLVGGLAGFWAAQIEGPRIGRFDKGGAATLVLKGHSASLVVLGTFLLWFGWYGFNPGSFLIILSPYGAGSFTGNWTAVGRTAVTTTLAGCSAAITTLFGRRFLTGHWAVVDVCNGLLGGFAAITAGCSVVDPWASLICGFVSAWVLIGLNLLAERFHYDDPLEAAQLHGGCGTWGLIFTALFAKEEHVLNVYGRTSTPYGLFMGGGGRLLAAQIVYILAIVGWVTVTMGPLFWTLHRLNLLRISPEDEVAGMDLTRHGGMAYYHQDGSHHDGHKFQLHNLSKHGGGNAPGMVHDAASPL

*Selaginella moellendorffii* AMT2;1

>gi|302793378|ref|XP_002978454.1| hypothetical protein SELMODRAFT_108685 [Selaginella moellendorffii]

MKCSYLSSSSTPCWMDKGSNAWMMVAAILAGLATMPGLLLLYSGIARKKWAVNTAFLTLYAFTASLICWVTICHNLAFGSHLLPFWGTPGPVLYTKFLLSRSQHPATHQDLDFPSATMVAFQFGFAANSVAIVSSAVSARITFQAWAVFVPLWLIFSYTVGASSIWSGGFFSRWGVLDFAGGYVVHLSAGVSGAVLAHWVGPRHPVDRARYPPNNVMLVLAGAGLVWLGWIGFAGGSAFLSPQQASLAVVNTNIAAATSLLVWTSLDVFYHGQPSVLGAVQGLMTGLVAISPAAGLVEGWASMCIGLCSGSLPWLTRLCHQKTTAKFCEEVDDTAGAVHTHGIAALIGVLLTGFFAHPRLTAMVSPVSGSEGVIFGNFQLVLKQLAAALLVILWNVAVTTLICVVVKRLMKLRMSDDQLRIGDDAVHGEEAYAVWEDGEKTTL

*Selaginella moellendorffii* AMT2;2

>gi|302764038|ref|XP_002965440.1| hypothetical protein SELMODRAFT_84585 [Selaginella moellendorffii]

MAARTTPITAVTPEWLEKGDNAWQLISATLVGMQSVPGLVILYGSIVKKKWAVNSAFMALYAFAAVLICWVSFAYKMSFGHQLLPFWGKAGPALDQKFLLRQAHLSATEYKFHNGTVETQMVQPFYPMATMVFFQFVFSAICLILIAGSLLGRMNIHAWMLFVPLWLTFSYTVGAFSLWGGGFLFQWGVLDYSGGYVIHLSSGVAGFTAAYWVGPRLTKDRERFPPNNVLLMLGGAGLLWMGWAGFNGGDPYAANIDASLAVLNTNICAAASLLVWTAFDTIFFGKPSVIGAVQGMITGLVCITPGAGLVQGWAAICMGILSGSIPWYTMMVVHKRSPFLQKVDDTLAVFHTHAVAGTLGGFLTGLFAQPTVTGMFSSVPGTHGAFYNHDIRGAMQVVKQLGGALFIISWNLVMTSIICWVISKIVPLRMPEEHLMIGDDAAHGEEAYALWGDGEKYDSSKHGLYNEAVEAGNRGSSRYNEPPVAV

*Selaginella moellendorffii* AMT2;3

>gi|302783527|ref|XP_002973536.1| hypothetical protein SELMODRAFT_173475 [Selaginella moellendorffii]

MATVPATPAYALAATVPEWLNKGDNAWQLTAATLVGMQSVPGLVILYGSIVKKKWAVNSAFMALYAFAAVLICWVGWAYRMAFGHKLLPFWGKADVALGQKYLMQQASIPSSGHYYKNGTVETAEITPFYPMATMVYFQFVFAAITVILIAGSLLGRMNFRAWMIFVPLWLTFSYTVGAYSLWGGGFLFHWGVIDYSGGYVIHVSAGTAGFVAAYWVGPRLTKDRERFPPNNILLTLTGAGMLWLGWNGFNGGDPYAANIDASIAVLNTNICAATSLIVWTILDVTFFNKPSVIGAVQGMITGLVCITPGAGVVQSWAAIVMGICSGSIPWFTMMVVHRRSTLLQKVDDTLGVFHTHTVAGVLGGGLTGLLAEPVLTSYFVPVTGSRGGFYGGVGGGQFGRQIAGALFIIGWNIVATTIILLAIRLFVPLRMPDDELQVGDDAVHGEEAYALWGDGEKYEASRHGNDDIHHDGGNGRKVTVQV

*Solanum lycopersicum* AMT1;1 (EMBL)

>sp|P58905|AMT11_SOLLC Ammonium transporter 1 member 1 OS=Solanum lycopersicum GN=AMT1-1 PE=2 SV=1

MACSVDTLAPFLGPNTTNAVAAASYICNQFSGVSDRFVDTGYAIDSTYLLFSAYLVFSMQLGFAMLLAGSVRNTMNIMLTNVLDAAAGGLFYYLFGFAFALGGPSNGFIGRHFFGLKEIPSNSFDYMNFLYQWAFAIAAAGITSGSIAERTQFVAYLIYSSFLTGFVYPVVSHWFWTPDGWASPTNSNLLFGSGVIDFAGSGVVHMVGGIAGFYGALIEGPRIGRYDHTGRSVALRGHSASLVVLGTFLLWFGWYGFNPGSFNKILVTYGASGGYYGQWSAVGRTAVTTTLAGCTAALTTLFGKRILSGHWNVTDVCNGLLGGFAAITAGCSVVEPWAAIICGFVAALVLIGFNMLAEKFKYDDPLEAAQLHGGCGAWGIIFTGLFAKGEFVDQVYPGKPGRPHGLFMGGGGKLLGAHIIQILVIIGWVSATMGPLFYILHKFKLLRISSEDEMAGMDLTRHGGFAYYHEEDPKLGMQMRRIEPTTST

*Solanum lycopersicum* AMT1;2 (EMBL)

>sp|O04161|AMT12_SOLLC Ammonium transporter 1 member 2 OS=Solanum lycopersicum GN=AMT1-2 PE=2 SV=1

MASAMTCSAAELFPHLGSSANATAAAEFICSRFSAVSEYLTNTTYAVDTTYLLFSAYLVFAMQLGFAMLCAGSVRAKNTMNIMLTNVLDAAAGGFSYYLFGFAFAFGAPSNGFIGKHFFGLKEFPSQAFDYSYFLYQWAFAIAAAGITSGSIAERTQFVAYLIYSSFLTGFVYPIVSHWFWSGDGWASASKTDGNLLLRFGVIDFAGSGVVHMVGGIAGLWGAFIEGPRIGRFDRSGRSVALRGHSASLVVLGTFLLWFGWYGFNPGSFLTILKSYDHTIRGTYYGQWSAIGRTAVTTTLAGCTAALTTLFCKRLLVAHWNVVDVCNGLLGGFAAITSGCAVVEPWAAIVCGFIAAWVLIGFNALAAKLKYDDPLEAAQLHGGCGSWGIIFTGLFAKKEYVNEVYPGFPNRPYGLFMGGGGKLLGAQVIQVVVIIGWVSVTMGPLFYLLHKFKLLRISRDDETAGMDLTRHGGFAYIYHDEDEGSSMPGFKMTRVEPTNTSTPDHQNRSVNVVV

*Solanum lycopersicum* AMT1;3 (EMBL)

>sp|Q9FVN0|AMT13_SOLLC Ammonium transporter 1 member 3 OS=Solanum lycopersicum GN=AMT1-3 PE=2 SV=1

MDSSWEASVTDSINAIYLLFSAYLVFVMQLGFAMLCAGSVRAKNAMNIMLTNVVDAVVGSLSYYLFGFAFAFGDSNPFIGASYFALKDIPSSSYDYSFFLYQWAFAIAVAGITSGSIAERTQFTAYLVFSFFLTGFVYPVVAHWLWSSNGWLSPNSTYLLFGSGAIDFAGSGVVHLVGGIAGFWGSIVEGPRVGRFDAFGNPVKMRGHNATLVVLGTLLLWFGWFGFNPGSFNKILVAYPHMADQGNWTSVGRTAVTTTLAGSTAGIVTLFGRRLLVGHWDAMDVCNGVLGGFVAITSGCSVVEPWAAILCGFCAAWVLIGLNILALKFKFDDPLEAAQLHGGCGAWGLIFTGLFAKEEFVLQAYNSGKTQIIRPSGLILGGGWGLFGAQIVELLSIVVWVSLTMGPLFYLLQKLGILRISSDEEVAGLDISSHGGYAYDASQEESNARFYGEYLRMQQQ

*Volvox carteri* 1 (JGI)

>Volvox carteri(JGI 87801), Vocar.0028s0082.1.p - (M=11) PTHR11730 - AMMONIUM TRANSPORTER

MDFVPEQDAPYSDLLILHGAIVFFLQVGFVSLEVGYGRSKNVKNILLKNTVNILICAIVWWSVGYAFSFGGSAGGFIGTSGFFHDGSLGAVKIWFYSWTFCLATVAIVSGCLAERTSLVAYPVLTLLMASWVHPVAAHWTWQRGSWLLGISQECRFLDFAGGAAVHVCGGLMGLVGATVVGPRIGRFEDGRAKDMPGHDVSSAAIGTLFLWFGWFGFNCGTTYVYLGSMAAGGSPPPAASSSAAAAGQPAGSPADRVALNTTLTASISSLTALLLSSIRSGTVDLVVCCNALLAGLVISTPAAGFITSWAAVVYGLAAAGIYLGGARLLIRMQVDDPLESSVVHFACGMAGTLLLGFLARPPYVAQLTGFTCGGLVYGGRKGGVLLGLQTLGVAAVAAWTGAFSVLAFWALRRTGRLRVDQVTELAGLDNMEHGGPAYPEFNLVPYNNGSAER

*Volvox carteri* 2 (JGI)

>Volvox carteri (JGI 69712), Vocar.0019s0251.1.p - (M=11) PTHR11730 - AMMONIUM TRANSPORTER

MGASSFSEPLGKCDLTTLQSLQNYGLQEADINQLCAPPKGCQGFQDCLNVYLLARTAAANTKASDVARSLDVAFMLYNGYMVFMMQLGFAVLCAGSIRTKNCMNILLKNVLDACVGAIGFYLFGYAFAFGLKPGSDGNGFIGNWNFALSYTTQISPDGGSDFSSSGWHNFFFQWSFCAATTTILSGAVAERCNFIAYLLYSFFLSSFVYPVVVHWVWGANGWLSVYNTSDGGYPSILKVGAIDFAGSGVVHMTGGFASLMGAWIIGPRVGRFGADGTVNEMKGHSATLVVMGTFLLWFGWFGFNPGSNLIISTPLAAQTVSRVAVCTAMSAGAGGVGMLLYKYLVTRSWDTVAVCNGVLAGLVAVTGASAVIEPWAAIICGLIAAVLFCLFDYLTLYVLKIDDPVSAFPLHGGVGMFGVLFPGLLARPDYVQQVYGSRIWGVDAKDTKRYGILYGGHGQILLANFIELVTVIGWSCFMMGTFFYLLHKAGLLRVSVETELVGLDVSHRCRSQGGLNGGNTKSLPECLYSITEHTGEKGLAP

*Volvox carteri* 4 (JGI)

>Volvox carteri (JGI 65690), Vocar.0008s0224.1.p - (M=11) PTHR11730 - AMMONIUM TRANSPORTER

MAVGSCDEVLFARVLALLGGDAATARSICGAPDVAILGADGAVTRWMVGQWRTTQQALQQVQNGLNVSFVLTSAYQIFVMQLGFALFAAGVVRPKNIVAIFLKNLFDTCIAGIVFYLVGYAFAFGAKDGDANGFIGNWNFALSSSGAGAGRSGDGPQPWHLFVWNWSFCSAATTILSGSIAERGTFVAYSIYAAVMPSWVYPVVAHWLWSPFGWLSVRNGPNRILGIGAIDYAGSGVVHLVGGTAALVGSFMVGPRVGRFGGDGISPQVYRATAAPQLYLMGTLLLWFGWYGFNPGSRLQIADDVSSMVVGRTAVVTTLSACAGALTGLLLSYTRHKVWDLLSTCVGALAGLVSVTSGCSVLEPWAAIICGIIATFVCEAGDDLLERFKIDDPVSAFPLHGLGGAWGLIFTGLMAKESYIQQVYGVAPGGHRMGLFYGGHAQLLLCQSIAVAVIAGWTLTNMIVLFYVLKRARILRVPADKEVAGMDLTECGQFNARGAVHLQSSKLLTMKGDILLSAFSVERRINGITGFDVSLRNRSTSHAHAGLIMACVPADGTAAVVTSATAAGGSAAAVAIASDASAAGISTINSCGCADAVGDCGGTVNAAAALMVTDAPPPTPDRDGSVHIPTPNGEA

*Volvox carteri* 5 (JGI)

>Volvox carteri (JGI 61118), e_gw1.22.94.1

MHTLSLTQALCSQFASSSKLQDAIIGLNTLYLLLCGALVFVMHAGFAMLCAGAIRSKNTMNILLQTVMDAAVSAIAFYVLGYGFAYGVGDKPNGFIGDALYGLSRWRSHNYDLATGMGGDTGLGWQSWFFQWAFAATATTIPAGAVAERLNFNAYLVYSAFISAFVYPVVVHWVWSTEGWLGYVRRAPYGRLFGSGMIDFAGSGVVHMTGGLAGLAGCVLLGPRMGRFDSSGRPVEMPGHSASLVVLGTVLLWFGWYGFNPGSQLLINSLANAAVVGRAAVTTTLSGAAGCLTCLGVAFTRHKAWDLVAGCNGALVGFVTVTAGAHVLEPWAALIAGSTGALVFEMGCALLLKLKVDDPLAAAPMHAAGGAWGVVFVGLLAKKQYICESYGKDCTEPGSGVVPHGLFYGGGGRLLASQVIGVISIFAWVYGLLTLLFAVLNAINLLRISAQEEQAGLDLSKHGGSAYYYDHGLGKPEKAPTAAESVLTPSPPSTSI

> Volvox carteri (JGI 61118),Vocar.0049s0040.1.p - (M=11) PTHR11730 - AMMONIUM TRANSPORTER

…MHAGFAMLCAGAIRSKNTMNILLQTVMDAAVSAIAFYVLGYGFAYGVGDKPNGFGWQSWFFQWAFAATATTIPAGAVAERLNFNAYLVYSAFISAFVYPVVVHWVWSTEGWLGYVRRAPYGRLFGSGMIDFAGSGVVHMTGGLAGLAGCVLLGPRMGRFDSSGRPVEMPGHSASLVVLGTVLLWFGWYGFNPGSQLLINSLANAAVVGRAAVTTTLSGAAGCLTCLGVAFTRHKAWDLVAGCNGALVGFVTVTAGAHVLEPWAALIAGSTGALVFEMGCALLLKLKVDDPLAAAPMHAAGGAWGVVFVGLLAKKQYICESYGKDCTEPGSGVVPHGLFYGGGGRLLASQVIGVISIFAWVYGLLTLLFAVLNAINLLRISAQEEQAGLDLSKHGGSAYYYDHGLGKPEKAPTAAESVLTPSPPSTSI

*Volvox carteri* 6 (JGI)

>Volvox carteri (JGI 41671), gw1.19.140.1

QVENLRNGVNQFYLLFGTALVFVMHGGFAMLAAGAIRSKNAVNILLQTLMDACASALMWYLIGFGFAFGIGDKPNPFIGDAMFALARVTTHNTGSGTGRWLDFVFQWAFCATAVTIPAGSVAERCNFNAYLCYSTFISAFLYPLVAHWVWCSKGWLGYGTLKPLLRCGMMDFAGSGVVHMLGGLSGLAGAVLLGPRMGRFDIENQPVPLPGHSAVLVVLGTVLLWFGWYGFNPVSTLFIDTAAAADVASRSAVTTTLAGAAGGVGCLIWTLLRTRTFDLYALCNGVLCGFVAVTAGCHVYEPWAAILCGSGAGIIFDLACILLIKLRIDDPLGAAPMHGGCGAYGVLFVGLLAKKEYIMQTYTRDGYPHGLFYGGGARLLACQVIGALTIAGWTLGVMFPFFWILKVRGLLRAPPEEELNGLDISR

>Volvox carteri (JGI 41671), Vocar.0054s0046.1.p - (M=11) PTHR11730 - AMMONIUM TRANSPORTER

MKCPPEILSSLSAALSSLPNPEAVALAICDATRETALNVENLRNGVNQFYLLFGTALVFVMHGGFAMLAAGAIRSKNAVNILLQTLMDACASALMWYLIGFGFAFGIGDKPNPFIGDAMFALARVTTHNTGSGTGRWLDFVFQWAFCATAVTIPAGSVAERCNFNAYLCYSTFISAFLYPLVAHWVWCSKGWLGYGTLKPLLRCGMMDFAGSGVVHMLGGLSGLAGAVLLGPRMGRFDIENQPVPLPGHSAVLVVLGTVLLWFGWYGFNPVSTLFIDTAAAADVASRSAVTTTLAGAAGGVGCLIWTLLRTRTFDLYALCNGVLCGFVAVTAGCHVYEPWAAILCGSGAGIIFDLACILLIKLRIDDPLGAAPMHGGCGAYGVLFVGLLAKKEYIMQTYTRDGYPHGLFYGGGARLLACQVIGALTIAGWTLGVMFPFFWILKKLKLLRAPPEEELNGLDISRHYSRAYNMDHPEAAVMPVTAFTNGGIVTYVTKDGGAVGDAIMRAELVGTSSSDGAPAVAWVTPATFAKGDSAKTQQLQQLGPGPVALMPMASSRRPAAETQQQQKQGQELSGIPAAGWGPLSQQQQQQQQQPPLPGTFGVQNNSGVPSFGGTTMEAQLQKLKSFDDGGLEKGAVAMGTKKSPSQKAGLPFAKSAKDMTLEAYNDDDRAANGAGKLSPAGEQVGAPSPPSPSSPRNQPAAVAAARVMMPAAASSRSMKKAVVAPLQTAGVSGNFEDDGADISVHRLAA

*Volvox carteri* 7 (JGI)

>Volvox carteri (JGI 77636), Vocar.0022s0058.1.p - (M=11) PTHR11730 - AMMONIUM TRANSPORTER

MAEAESCFDRIQKALTDANTPESMASAIATAICDELSTSASSVADVSLGVNTFLLLFGGALVFLMHGGFAMLEAGAIRSKNAMNILLQTILDAAAAAIMWYCVGFGFAYGIGDKPNKFIGNAMFGLARYKSHYTGSGMGMWTDFFFQWAFCATAATIPAGSVAERFNFNAYMGYSIFIAGFVYPVVAHWAWCVEGWLGFGVNTPLFKAGYIDFAGSGVIHMTGGLAGLIGATMVGPRLGRFDADGNPVEMPGHSAILVVLGTVLLWFGWYGFNPTSMLMITSTPAATVVGRAAVTTTLSGAAGGIACLLNGFRRHRGWDLVGLCNGILCGFVAITACCHVVEPWAAILCGLVAGLWFDFLCWVLLKLKIDDPLSAGPMHFGTGMWGVFFTGLLAKQEYIKEAYTFRSDDVDAYHLYGAFYPHSSGKLLASQVIGLLAIIGWVCGTMIPFFFIFKLAGALRIPPEMEEMGLDRSKHGGSAYNGTGVSPSAPGNDVMRNQPSAKVLPLNG

*Volvox carteri* 8 (JGI)

>Volvox carteri (JGI 67734) e_gw1.67.86.1

MSNQGPLSTGGCTSEQFAEVVKALDGDSAAATALCQVFATSNRTEEIAFGLNTVFMVLSGALVFIMASFAMLEAGAIRSKNAMNILLQTVLDGAASAIMWYLVGFGFAYGIGDRPNGFIGDALFGLARYKSHETVTDVRGDWTSWFFQWAFCATAATIPAGAVAERFNFNAYLGYSLFLGGFVYPVVVHWVWCRTGWLGYGTDAPLLGAGMIDFAGSGVVHMTGGLAGLIGATMVGPRMGRFDSNGQPVHMPGHSAILVVLGTVLLWFGWYGFNPGSALVADNANVASVAGRAAVTTTLSGAAGGLTCLVIAVWRGRSWDLTYLCNGMLVGFVSITAGCSVLEPWAALLCGGVAAVLFEAACEGLLKLRIDDVVSAGPMHGVCGAWGVLIVGLLAKKEYVEQTFDRDIYPHGLFYGGGGKLLASQVIGILAIAGWVFGTMGPFFAAFRAVGSLRISAEDETLGLDASKHGVRNLGRAGVRKAMPSCRTLDPLNCVVSHISCRVESAASFPITPSSCTT

> Volvox carteri (JGI 67734) Vocar.0051s0018.1.p - (M=11) PTHR11730 - AMMONIUM TRANSPORTER

MSNQGPLSTGGCTSEQFAEVVKALDGDSAAATALCQVFATSNRTEEIAFGLNTVFMVLSGALVFIMHGGFAMLEAGAIRSKNAMNILLQTVLDGAASAIMWYLVGFGFAYGIGDRPNGFIGDALFGLARYKSHETVTDVRGDWTSWFFQWAFCATAATIPAGAVAERFNFNAYLGYSLFLGGFVYPVVVHWVWCRTGWLGYGTDAPLLGAGMIDFAGSGVVHMTGGLAGLIGATMVGPRMGRFDSNGQPVHMPGHSAILVVLGTVLLWFGWYGFNPGSALVADNANAATVAGRAAVTTTLSGAAGGLTCLVIAVWRGRSWDLTYLCNGMLVGFVSITAGCSVLEPWAALLCGGVAAVLFEAACEGLLKLRIDDVVSAGPMHGVCGAWGVLIVGLLAKKEYVEQTFDRDIYPHGLFYGGGGKLLASQVIGILAIAGWVFGTMGPFFAAFRAVGSLRISAEDETLGLDASKHGGSAYNMPTTLHQFIGGLGDMTGTGDNSNHGRYSLNPHLYSTGGGRAVGDSIMRAMTIGGGGGGGGTVLGAGGSSAAVAGPANGTPVGAPFGGLYNILGTNAPSPPASPTAGQSYGCRTGAGNGFEANAGSDVPRNFEQGAPAVRLHVSVGRTVSGQLMDVGRRPGDTSVGGGAAIAAVPAASAASVLDSKADVQLSVAADAIRTRSGSGSRKGRSGWGAASGKAAEAPAEGSGAVATTSSPPRELSAAAATTTTTAAAAVAAAT

*Volvox carteri* 9 (JGI)

>Volvox carteri (JGI 92451)

MRVNVFMQIGFVSAESGQGRAKNVRNILLKNSVNIMLCAICWWAVGYAFAYGKTAGGLLGISHFFSDGETFDSKPWFFTWTFCLSAVTIASGCLAERTHLFVYPVYTAVVSIVVHPVVAHWVWGKDSWLNSRVGSPCRFLDFAGGAVVHIVGGLTGLIGAILCGPRMGRFEDGVQKDIPGHDVSSVSLGSLMLWFGWFGFNCGSTYIYMSDGGPAASSAVSRVALNMTLCASAAGMTSLVLASLLSGGSAGTFDLGVCCNGLMAGLSASTANVGFITPWASCITGTLAGLLYVASSRLLVRLGIDDPLDSSAIHCGSGLLGVVISGFLARPSYVRSMVDKNCGGLVYGMNGGTQLGMQLLGKYCRISGRNGFGGKRGGRVMTLWGGLRRWVKSDPWGSYVVRRLLVTIAWTALWSILAFSTLKRFQLLRVDQQTELAGIDNMEHGGPAYPEFLQRVQSSRTGF

> Volvox carteri (JGI 92451)Vocar.0001s1695.1.p - (M=11) PTHR11730 - AMMONIUM TRANSPORTER

MHEGTKNQEFGTIRHCQGGKLLAKNTLQLFDGILLYLKGAWNRLIGHISIKEKPVEPNLALPYLPVRLTFAAVLMGSAVATGVDSDIWQDHNRFLQEQASSHESSRSTASYTASDFVLLNGMRVNVFMQIGFVSAESGQGRAKNVRNILLKVRIVYTTFPNRPPYSPIKSAFPNTPNPQKPPPPRKQNNKKGKVPPKHAGSPTWPPNSVRLLDNLRVCVCFPLNPRGTFEKFWILSQSCGQNSVNIMLCAICWWAVGYAFAYGKTAGGLLGDGETFDSKPWFFTWTFCLSAVTIASGCLAERTHLFVYPVYTAVVSIVVHPVVAHWVWGKDSWLNSRVGSPCRFLDFAGGAVVHIVGGLTGLIGAILCGPRMGRFEDGVQKDIPGHDVSSVSLGSLMLWFGWFGFNCGSTYIYMSDGGPAASSAVSRVALNMTLCASAAGMTSLVLASLLSGTFDLGVCCNGLMAGLSASTANVGFITPWASCITGTLAGLLYVASSRLLVRLGIDDPLDSSAIHCGSGLLGVVISGFLARPSYVRSMVDKNCGGLVYGMNGGTQLGMQLLGKYCRLLVTIAWTALWSILAFSTLKRFQLLRVDQQTELAGIDNMEHGGPAYPEFLQRVQSSRTGF

*Volvox carteri* 10 (JGI)

>Volvox carteri (JGI 75428) Vocar.0069s0013.1.p - (M=11) PTHR11730 - AMMONIUM TRANSPORTER

MSDPLQECIDAIKPLFGDDADKAAVLCGQFAPITVGDQLNDAVVGLNTLYLTSCGALVFVMHAGFAMLCAGAIRSKNTMNILLQTVMDAAVSAIAFYILGYGFAYGIGDKPNGFIGDALFGLARWAPKNVALNPAAGDGWQNWFFQWAFAATATTIPAGAVAERLNFNAYLIYSFFISAFVYPVVVHWVWASEGWLGMARYIGYSHLFRSGMIDFAGSGVVHMTGGLAGLAGCIMVGPRMGRFDSNGNPVEMPGHSATLVVLGTVLLWFGWYGFNPGSQLLINTAVSASVVGRAAVTTTLAGAAGCLSCLLTAFFRHKAWDLLAGCNGALVGFVSITASANVVEPWAALICGLMGGWVFDFVCWVFLKLRIDDPLSAAPMHAFCGAWGVFFAGLLAKRQYVCDSYGRDCDNTYYIPNGLLYGGDGRLLASQVIGIICIFAWVFGLMSILFAVMKVLKILRISAEEEQAGLDVSKHGGSAYNYDHGLGKPEKAQAMGL

**Volvox carteri*

>Volvox carteri (JGI 84428), Vocar.0063s0027.1.p - (M=11) PTHR11730 - AMMONIUM TRANSPORTER

MSVPTILYSCTPEQTQEVSNLVQDANIASAICHNYDCSVSAASCVLRYLATQQQTLIEEVQQTAKAEVSLSLDVAFVLFSAYLVFGPMQLGFALLCAGAIRSKNSMNVLMKNILDACTGAIGFYLFGYAFAFGHEAKKSSNGFIGNWNFALSFTTKESSLSNVLEFDDDFPDEGWHSWFFQFSLCAIATTIVSGAVAERCTFGAYLAYSFFISAFVYPVVAHWVWSPDGWLSAFNTSKGGYALILRSGAIDFAGAGVVHVTGGMAALMGAWIIGPRIGRFDASGKVNEIKGHSATLVVMGTFLMWFGYYGFAPGANLSVATADAAVVVSRVAVTTTLSAASAGLTTLFLRYAMSSTWDTVLVCNGCLGGLVAITAGCAVVEPWAAVLCGSAAALVFVGSDYLLLYKLKIDDPVSAVSMHLFCGLWGLFFPGLLAKPQYMADVYGDDGLADSVKAAGKYGILYGGHGQVLLCQLIEAVAICLWVGLMMGTFFYVFKAAKRLRVPVDQELAGQDVTKEGVEPYDGAGGSVGPQQGVGTTAAFGPGATVTTSAFYSNGEGEEGVSGGYATND

**Animals**

*Anopheles gambiae* AgAMT

>gi|31323879|gb|AAP47148.1| ammonium transport-like protein [Anopheles gambiae]

MANGTTMGSPGAASTTPSPGAFQSLARNNSYVIPGLYDLNVEDTNWVLTSSFIIFTMQTGFGMLESGCVSVKNEVNIMMKNIIDIVLGGFTYWLFGYAMAFGRGELNNPFVALGDFLIDPGVSDPLFGPIFAAFLFQLSFSTTATTIVSGAMAERCNFKAYCIFSFFNTIVYCIPAGWVWGEHGFLKNLGVVDIAGSGPVHLIGGASAFASAAILGPRLGRYAKGTDPLPLGNPVNACMGLFVLWWGWLAFNSGSTYGVSGAKWAYAARAAVMTMMGSFGGGSFSIIYSMINNDGRMDVVDLINGILASLVSVTAGCYLYHAWEAILIGAIGSALCCFGMPLFDRMGVDDPVGASSVHGIAGIWGVLAVGFFADNPLRMDTTSGRSGLFKGGGWYMLGVQSLSALCLACWGVCSTFVLLWLINKVVPIRMDPNEELLGADLMEHRIRHTQIGISRALSALAPIQLDLDDVIDAPPIGRNPGHERCVDEIEAASQKLHQWRQAMDKFADRPAREKTEPASGASSRRRSKSFLSKSHRNGKDNAAFERGGITTVKMSGREGKETHQLSVIGSEQSYQVGSTDRNADRNFAWID

*Branchiostoma floridae* 1

>gi|260805384|ref|XP_002597567.1| hypothetical protein BRAFLDRAFT_165534, partial [Branchiostoma floridae]

MQAGFGFREAGSVRSKNTTNILIRTMLNVFISGIAYWSIGYAFAYGTPSNFFLGHSNFFLSLPVVDVTHARWFFQFTLAATCVTIVSGALSERVEFAACLVYAMLISGFLYPVVSHWAWAPTGWLRTGAGDKGLVFPDFAGSGVVHMTGGAIALAGAIMVGRRIGRFEGRRAVRIPEHNVPLVSLGTFMIFLGLLAFNVGAQGTISRSGSGEAVSVITVNTIISASIGGLVAMLMKRVGILGKPNWTYLSAANGALTGMVSVAAGCDMVYTWGALVIGTVAGLTYCVWSRLLQALRIDDPVDTIAVHLGGGFWGLVAAPILSRNGIIQEVSTMNLKVRFDRFLPTIKRLLGWNLAGAVAIFGWGVATAVVIFGIMKCFGALRVDGEVEMQGLDVPKHDEPAYPQDGYGHGW

*Branchiostoma floridae* 2

>gi|260830860|ref|XP_002610378.1| hypothetical protein BRAFLDRAFT_72414 [Branchiostoma floridae]

MAANLTILAADFATLQKNADQLFLLIMGCMVFLMQSGFAFLEAGSVRSKNTTNILIKNMLDIFITGLCYWMFGYAFAFGQVGNPFIGHSDFFAGSLRHSPDPAFWFFQFVFCATATTIVSGAMAERTEFVCYLVYCVVIGGFMYPVVTHWAWSDVGWLANGPGGIVYKDFAGSGVVHVVGGTTALVGAKILGPRIGRFKDGQPVQIPGHTVPLVALGAFILFFGFLAFNGGSQASISGAGDGQIVSIAMVNTLVSGAAGGLVAMMVKRSGLLGGVNWSLLTSINGALSGMVAICAAADSAYSWGAVVIGVVAGVVYCAWSKLVIKLGVDDPLDAVAVHLGAGLWGVLAAPILARDVGLITADKEYALRLFGWNLLGVLVIMVWCAVVSALMFGTLKCLGILRVPPEIELRGKLRFFVWILS

*Branchiostoma floridae* 3

>gi|260805452|ref|XP_002597601.1| hypothetical protein BRAFLDRAFT_225820, partial [Branchiostoma floridae]

MFSLVFPVMQAGFAFLEAGSVRSKNTTNILLKNILDVCIGAISYWAVGYAFAFGTPSNLFIGHGHFFLEGIEGSQYAHWFFHFVFAATASTIVSGAMAERTEFSAYLVYCAVLTGVVYPVAAHWAWDPKGWLLTGVGTGLSFQDFAGSGVVHILGGTAALAGASVVGPRIGRFQHGKPVQISGHTVPLAALGAFILFFGFLAFNGGSQGAISNPGDGVVVALAITNTIISGDAGLIAMVIKRVGLLGDLHWSLLTTLNGALTGMVAICAGCNNVYPWAACIIGVVAGAAYCGWSQLILKVLKVDDPLDAVAVHFGGGFWGVIAVPIFATEGGIITSGGSVMAFKGLGWNILGGIAIAVWTGIITTTMFGFLRLAGVLRVDPEIEEKGLDIPKHGEPAYPQESYGHGW

*Caenorhabditis elegans* AMT-1

>gi|17550180|ref|NP_508784.1| Putative ammonium transporter 1 [Caenorhabditis elegans]

MTTPTNFTTEIDKLHAEITRLETGFYENVNSFFLCSMALIIFFMQCGFAYLEAGAVRSKNTTNILIKNLLDSCICIIGYWAIGWALAYGDSGEGVNLFVGHSQFFLSGFSDYPRFFFQYVFSATAATIVSGAVAERCEFITYVTYCTVISTFIYPVLTHWGWTENGWMAKGITSGIIDTKYDDFAGSGLVHLCGGSISFLAAWIMGPRIGKFPDDEDDESDEILGHSVPFTALGGFILMFGFLAFNGGSVASISHAGDGHTVALAMINTILSGAFAALIYLGVHYYQHGKWTLLLTINACLSGMVAACAGCNKMEPWACIWVGLGAGLIYLAFSKLMIRLKIDDPLDAFAVHAGGGFWGLMSSSIISHGGVAYALADAVSGAKNSGDHLTQAFAQLGWQMICALAIIAWSLGVMLPIFWILKKTGKLRVSEEVEINGLDVFKHGEMAYPLRAYGHGWHDFERANKIQAFSAKITVGEGKNTRIMKIHPEMSIEQLASVYDRSGNIIPMPKKSRTLFTNSAERKMSQMMYDENKM

*Caenorhabditis elegans* AMT-2

>gi|392901400|ref|NP_502496.3| Putative ammonium transporter 2 [Caenorhabditis elegans]

MNTLQNLTLKMDRQPTIRMKPDKQLDCSLSQDDGVWMMASSFIIFTMTAGFGLLESGRVSSKDEVNCMVKNVFDVIFGGLAYWMFGYGLTFGDSKHQLGRYVGFGDFFFDPERVSDDDSTDEKGISYSLFIFQMSFATTTSTIVSAGMSERIHLKSHCFISFFITLVHSVAGHWVWDQEGVFRMMGVVDSAGCSAVHLVGGVSGLVATLYLKPRRNRFAKNGIRTVSDPTKAILGFLMIWWGWLAFNTSSNYAVTHGQWTEGMRSAVGTILASAGGGVVTVIITRLSTKKIQMDMLIDGMLASLVASTGGCLYFTPWQATLVGAIGSSLALAAYPVTEWLKIDDPVGVFPVHVVGSIWGMIAPAIFVYRRPMNFGPPECDFQTSDEINGLLYGGGFYLLFLQSFVILVIGTYSAICAFIILFLIHHSPVGLRVDKYTEELGADLIEHGLAGFNVMTYTIEKKLDTKTLSAVLMIIVRWRAKAKLGAQRRKKIHDSGSVAPQQAESVEMNVIHRRH

*Caenorhabditis elegans* AMT-3

>gi|32564042|ref|NP_495761.2| Putative ammonium transporter 3 [Caenorhabditis elegans]

MAGPEGSIFNASAMQIVQIHHYAEGSVTPEVDKLYQDDAVWIISSSFIIFTMHSGFGLLESGSVSAKDEVNIMVKNVVDVVFGGLSYWSCGFGFSYGDIPEWRNPYVGFGKFFYDPTRDYGTRETINQEGWSYASFLFQLSLATTASTIVSGAVAERAKLKSYILLGCIVILIQALPAHWVWDKEGVFYKKGVVDFAGCSAVHLVGGIIGLIATVFLKPRRNRFNEDSVHQMSSPTNALLGTFLLWWGWFGINAGSVWGITGGRWRLGARAAVATIMASIGGGATAITISFVKTKKLQVNFLINGILSSIVSITAICAVSRPWHALVIGSISSVFSIAVLPLLDRLHIDDPVGIVPIHLTSSIWGMIAVGIFCEEDKYLGSATNNRSGLLYSWSFELLWVQLQCTAAILIYSATTGFLALFLISKSPLGLRVTDYEEQIGADVIEHGLAGTNVARYVLEKPLSTRTFQTVTKAITKWKMLAKKKSRQKRMEAAKLKRQEEQETFTNGTAIANGNGNVLHHRTNATESNGTGAPKRSNGPAFNNQITPLAVSSTVSTARNGPSTGRRTESTAIEIEQPIEAVPPEVVAAAVLPPEERPGPSTNSNVSIEASVEKSPSSSTSRRSISIRSSPSIHTVSAISTAAPDSRPSTASATSIISKKSSKNSTVGKFVKAPAPRALSPPDNNPPV

*Caenorhabditis elegans* AMT-4

>gi|392925874|ref|NP_508783.2| Ammonium transporter [Caenorhabditis elegans]

MATPNYTAQIILLQQEIDEMKEDFSENDNAFFLCSMSLIIFLMQCGFAFLEAGAVRSKNTTNILIKNLLDSCIAIVGYWALGWALAFGDCPNNTIGLFVGYSEFFLANFSNYPKFFFQYVFAATSATIVSGAVAERCEFANYITYCSVISTLVYPILTHWGWHPKGWMALGITSGVINTHYDDFAGSGVVHLCGGSISFLAAYMIGPRIGRFPEDDDDECDEILGHSVPFAALGGFILMFGFLAFNGGSMADIVKPGEGHIVALAMVNTILSGAFAALTYLIAHYLYHGKWTLLLTINACLAGMVASCAGCNKMEPWACIWVGVGAGLIYLGLSKLMVRLKIDDPLDAFAVHAGGGFWGLTSVAFIGHDGVVYSIGNTIGGATNGGDQIAQAFAQLGWQWVCALAIVTWSILWMWPIFALLRKIGKLRVSAEVEINGLDIYKHGESAYPLHAYGHGWHDFEAAPDSKINHSKHLPVGRKNRIMSVHPEMSLEQLASVYDRTGSVGESDQPKRLFMNQTERRKSRMIEANALHALYLDDNEVPERKNTNPKTVSLQVPTTIIEAAEDEADKMTENDRM

*Capitella teleta* 1

>gi|443695561|gb|ELT96437.1| hypothetical protein CAPTEDRAFT_62344, partial [Capitella teleta]

DDAIWILTSTSIIFTMQSGFGLLESGSASTKNEVNIMVKNAVDVIFGGLTFWLFGYSFSFGQDPKYTNFFTGWGNFLAFSSDENMGNVYSKFFFQASFATTATTIVSGEEACAMAERTRLESYIIFSLFNTLVYCFPAHCVWGKKGWLAELGMIDVAGCGSIHVVGGVAGLVATTILRPRHGRFGQEGKTPMMGSPTNVVLGTFMLWWGWLGFNCGSTFGVSGDKWKLAIRSAMATLNSSLAGGIMGCLISYIFKKRTFDVPYMVNGILGSLVGITALCALAEPWESFTIGAIGGTFALAGAEIVTKLKIDDPVGCVGVHAFGGFWGLVSVGLFKKKDSLSYSLGISEEVEGLFYGGGFHLLGIQMLGFVAIATWTAILSCLMLNTINLLVPLRMPLHEELLGSDIVEHGI

*Capitella teleta* 2

>gi|443726120|gb|ELU13413.1| hypothetical protein CAPTEDRAFT_23023, partial [Capitella teleta]

SGFGLLESGTVTSKNEVNIMIKNAVDVIVGGISYWMFGYAFSFGTGEWSNPFSGWGTFFVTANEKNLGSLYSKFFFQASFATTATTIVSGAMAERTKLEAYLFFSFINTLVFCFPAHWVWGDNGWLKTMGMVDIAGAGPVHLVGGVTGLVATLLLKPRHGRFLREEGEEWLAMGSPTNALLGMFMLWWGWLGFNCGSTFGISGFKWKLAARSAVSTLFGSMSGGLIALSSSYIFKRRKFEVDFVINGILGGLVAITGHCALAEPWEALIIGGIGAMLTLTTIELLAKARIDDPVGVVGVHAVGGTWSLISVGIFSRDDKLSGALAISHDQVGLIYGGGFKLLGIQMAGIVSIATWSAVTSFLILYAIDRVMTIRVPLQREILGADVVEHSI

*Capitella teleta* 3

>gi|443719784|gb|ELU09794.1| hypothetical protein CAPTEDRAFT_169940 [Capitella teleta]

MPSVSDVTSGIEDNFWEAAFETNCSTFRISCSSSSSLHCSWGSLTNNLSTTFQDQCFVSRGQTNVSSSGLGTDDKLTGRDASWILTSAVIIFTMQTGKFYFFQLFIWRFGLLEAGCVSPKSETNIMVKNALDVVFGGIMYWSVGFALSFGKGKGSNFFVGTGNYFVQIDDGEDIGFIYALFLFQLSFATTSTTIVSGAMAERTKLLAYNIFSMTNTLIYSVAAHWVWAENGFLHNWGAIDVAGCGPVHLVGGMTGLVAAIILGPRASAKHSQGGTKETLPSPLKAILGMFMLWWGWLRFNSGSVFIVSSSNEWKKAVRAAVNTIVASMGGGVTAVLVGKCTHQGRYDIGTIINGVLGSLVSVTAICTICAPYEAFVVGCIGALLTIAAEWVVRKLKIDDPVGVIPVHLVCAVWGLISVGLLVPEDESLVEGQAGLLKGGQWYLLGVQALSVVLIGSWTLVTSGVSLKVLDLTIGLRLTPEEEERGADIVEHGTTIRPDDLAFHLNNMNHMNSQEEMSIGETSLSNGISTEDIRKHVFRIKAQEMFKELLTSCQRNDLQVIINSSSLQSICLEFRVLPTSSSDDSGVGLHM

*Capitella teleta* 4

>gi|443711312|gb|ELU05140.1| hypothetical protein CAPTEDRAFT_140549 [Capitella teleta]

MSGRDASWVLTSAVIIFTMQTGFGVLESGCVHPKNEVNIMVKNALDVVFGGISFWVFGFGLSFGSDQGTNAFTGVGCFFVDAADEVFGDVFAVFLFQLSFATTSTTIVSGAMAERVKLHAYLAFCIVNTFTFSIVAHWVWSETGFLHTMGAIDAAGCGPVHLVGGVTGLVATIILKPRSGTFDRPKKPTLPSPLKALLGMFMLWWGWLRFCSGSIFIVSTDEQWKSAVRAAVNTMVSSMGGGITGVMILSRFCQRVKYDVSTIINGIMGSLVSITAICTLCKAWESLIIGIVGALIATATDSLLWKLKIDDPVGVVPVHGACSVWGLIAVGLFVDKNGTLTLRGGLLNSGDPHQLLVQFITVICIIAWTLFMSVTLLKILDCTLGLRFEEGDEKIGADVIEHGVSVFSSWKTRNLQTIKRISSTLSTNTGDTTVSQAVELNEFKQRVAILLHDMATSCNR

*Capitella teleta* 5

>gi|443699181|gb|ELT98791.1| hypothetical protein CAPTEDRAFT_22997, partial [Capitella teleta]

SGFGLLEAGIVHPKNETNIMMKNLVDVVFGGLSYWLFGYGLSFGQDSGNNPFCGIGHWFVTSKDHQMGLVYSNFIFQLSFTTTATTIVSGAMAERTKLDAYIIFSFFNTLVYCLPAHWVWADNGFLRTLGVLDIAGAGVVHLLGGVSALVAAIILGPRTGRFDKDSTTGNMGHPTNAVMGLFMLWWGWLAFNAGSTYGISGGKWKLASKSATTTMISSIGGGVQGIFMSYILTRKFLIDDIINSILGSLVSITGSCAVVNPAEGILIGVIGGFICMMMGRLMERIKVDDPVGAFPVHGIGGLWGLLAIGLFVDIDESEDTTMGQRGLFHGGGVKLLGVQALAAVIITAWTVLTTLIILLPLHFSLGLRLSVEDELLGSDFCEHGIDH

*Capitella teleta* 6

>gi|443706197|gb|ELU02379.1| hypothetical protein CAPTEDRAFT_205931 [Capitella teleta]

MNDSFVVNSTTESTEGPVRTTFALGDGVVSTWDDATWILTSSFIIFTMQSGFGLLEAGVVDSKNEVNIMIKNVVDVVFGGISYWMFGYGLSYGTDPGTNPFCGIGHWFVTSKDPDQMGLVYSNFIFQLSFTTTATTIVSGAMAERTKLTSYIIFSFFNTLVYVFPAHWVWADAGFLRQLGCVDVAGCGPVHLLGGVSALVGAAMLRARLGRYDSGKPYNTQPCNPTNSVLGLFMLWWGWLAFNAGSTYGISDGKWKLASKSATVTMIASIAGGVVGILASYATSGKYLVTDIINSVLGALVSITASCALVHPYEGVIIGSLGGLLCMFTPRAVDYFKIDDPVGAFAVHGMGGLWGIVAVGFFVDYDENQNMSNGQRGLLHGGGFYLLGIQPLKYTIGIRMDEIEETLGSDFVEHGIDQSARLAMLTCAPKQEAQSMNGRQNDVIAYPTTVKRRSYFGSLRDAPSIRVKRASPRKTVMDIEDRADTGISTTSLSQAASDVTQSDCF

*Ciona intestinalis* AMT-1

>gi|74136071|ref|NP_001027965.1| ammonium transporter 1 [Ciona intestinalis]

MFIFFMQCGFAFLEAGSVRSSNTVNILMKNTLDVFVGSLSFFAFGYPFAYGRPGNSFIGHNNFFGANVQYFEKSVIANNQTLGGYETSSSYFANFFFQYVFAATAATVVSGAVAERADFSCYIIYSFTITGFIYPVGAHWVWSPDGWLNTGAGTGLSMQDYAGSCVVHVCGGMAALAGAKILGPRLGRFSDDGLDKPIPGHSVPLVSLGAFILLVGFLCFNGASIQRISKNGDGLEVSLSVTNTILSGSAAGLSSMLTHRVHSMVKGKGKYWSLLMTINGGLTGMVASCAGCNDIAPWAALLTGSVAGVVFVFARQFVLWLKIDDPLDAISVHLFGGTVGTLAAPILAIRGQTINGVSVGGPIYGGGVDAWRGVGWSLVGLFSIGLWSGLCCFTLFTTLDMVGLMRIPKSVEESGIDMQKHGEHAYPETFVRIIDDRPETPRKNKVVKRNVASALVDKSLPAITTSAWDGQHPSEAGSSTNGCIEDPTRLSPSTAYNRK

*Ciona intestinalis* AMT-2

>gi|74136061|ref|NP_001027964.1| ammonium transporter 2 [Ciona intestinalis]

MQTGFAFLEAGSVRSKNTTNIIMKNVLDLFIGAIMYFAVGYSFAYGQPGNRFIGHNYFFATNKDEVMKTKILTLNPQIFSFLLTNKINTQNFTYASWFFQYVFAATASTIVSGAMAERTEFTTYMVYCTFLTGFVYPVIVHWCWTDVGWLQYGTGTGLKYIDYAGSGVVHLTGGTAAFVGAIILGPRIGRFNNDGTVNDLPGHTVTVAALGGFILFVGFMAFNGGSQLTISNPGDGAIVALSIMNTVLGGASGAIVAMLVYKTTDAIRGEDHYWSLLITINGGLTGMVSMCAGCNDMLPWAAVVIGTVAGLAFMTWHHIMLKLRIDDPLDAVAVHLGGGLTGVLLAPIFVVKGLQLAETKEYVGGIIYGKHWLAFQGFGWNLLGAVAIIIWTGVISILLFGSMSLLGVLRVSEEVELKGLDKLKHGEPAYPIESYCDGVPPWSKGKVKQCNIKV

*Ciona savignyi* Amt1

>Ciona savignyi, Amt1 (Huang and Peng, 2005) MSQQDEIELLKRQMAIMKENQNQFFMLINGMFVFFMQCGFAFLEAGSVRSVNTVNILMKNILDVFVGSLSFFAFGYPFAYGRPGNSFIGHNNFFGANVDYFEKTITVDNSTLAGYETSSSYFANWFFQYVFAATAATIVSGAVAERADFACYVIYSFTITGFIYPVSAHWVWSPDGWLYTGAGTGLRMQDFAGSCVVHVCGGVAALVGAKILGPRLGRFSEDGKLNAIPGHSVPLVSLGAFILFLGFLCFNGGSIQRISEDGDGLIASLSITNTIISGSGAGLSAMLANRVYNTVKGNGKFWSLLMAINGALAGMVASCAGCNDIAPWAALTIGSVAGVMFVVARNVVLWAEIDDPLDAISVHLIGGTIGTISAPIFAIRGQMVGGKPEGGPLYGGGIIAWKGVGWNLVGLLSIALWSGFYLGICCITLFSTLNMVGLIRIPKSVEEVGIDIKKHGELAYPEAFVRVISDEKKKKTEDGALKQRNLGADSKSLPIIKTSAWECDTNTPPTSSNGMTIDPALLSPNSVYSNL

*Ciona savignyi* Amt2

>Ciona savignyi, Amt2 (Huang and Peng, 2005) MQTGFAFLEAGSVRSKNTTNIIMKNVLDLFIGAIMYFAIGYSFAYGKPGNAFIGHNYFFATNMDENFTYAGWFFQYVFAATASTIVSGAMAERTEFTTYMVYFTFLTGFVYPVVAHWCWSEVGWLKVGMAHGLSYIDYAGSGVVHLTGGSAALVGAIALGPRIGRFKPDGTVNDLPGHTVTVAALGGFILFVGFMAFNGGSQLSISNPGDGNVVALSVMNTVLGGASGAIVAMLVYKTTDAIRGEDHYWSLLITINGGLAGMVSMCAGCNDMLPWAAVVIGTIAGLAFMGWHHLMLFCKIDDPLDAVAVHLGGGLVGVLLAPIFVMKGLTSPETQQHVGGIIYGGNYLAFQSFGWNLLGAVAIIGWTVAISTLLFGSMKLLGVLRVSEADELRGLDEIKHGEPAYPIKSYSDQSPPYLTCTGNGNGNGMNGNNQSTANFDQGIVMSNDVVLESDETHLEDEQHARTKL

*Drosophila melanogaster* CG6499

>gi|161078314|ref|NP_001097800.1| ammonium transporter [Drosophila melanogaster]

MNNGTVGAPRNHSNYVIPGLYDLSVEDTNWVLTSSFIIFTMQTGFGMLESGCVSIKNEVNIMMKNVIDIVLGGFTYWLFGYGMSFGRGPLSNPFIAIGDFLLDPPVGDALMGQIFAAFLFQLSFATTATTIVSGAMAERCNFKAYCLFSFLNTAVYCIPAGWVWGEHGFLNKLGAVDIAGSGPVHLIGGASAFASAAMLGPRLGRYSEGYDPLPLGNPVNACMGLFVLWWGWLAFNSGSTYGVSGAKWQYAARAAVMTMMGSFGGGFTSSIYSFWRHGGGMDIMDLINGVLGSLVSITAGCFLYRAWEALVIGAIGSLFCVLAMPLFDRMGVDDPVGASAVHGVCGIWGVIAVGLFADNPIPLDTTNGRSGLFKGGGWYLLGIQTLSALCLACWGVCSTFLLLYVINKIIPIRMDPHEELLGADLTEHRIRHSQIGLSRAISALAPIKVDLKDIAGIQPIGINPGHERSIDQLRAAEDKLQQWQSYLEQVSGSRANVKMDAGQSHADINFKPRTAGNVFNRRIPARKSISGGLYRSNNSELPVIGSVMGQKPEKDPNFAWVD

*Lottia gigantea* 1

>gi|676455191|ref|XP_009053925.1| hypothetical protein LOTGIDRAFT_188698, partial [Lottia gigantea]

MDGSNGTKDMNNTMTQMEMDFNALQSNLDQFFLLTNGMIVLLMQCGFAFLEAGAVRSKNSTNILIKNLMDQFFAGISYWLVGYAFAFGKGHWFIGYSYWASADMPQEDFALFFFQYVFAATAATIVSGAVAERCEFIAYIIYSVLITGFIYPVVTHWVWYGEGWLLTLGYLDFAGSGVVHVLGGIAAFVAAFFLGPRKGRFHKSSKTVAPIRGHSVPLAALGGFILLFGFLAFNGGSQLSISKTGDGSAISLAVVNTVISGSTSALFTLVINRAVGTSKWSLLTTLNGALAGMVAICAGCNVYYPWASCIVGFFAAISYRLINWSVTKLWIDDPLDAVAVHFGGGSWGVIAVAFLHKERGIIFNFDNESGLLLGYQLAGLLSIGVWTAVTCTIMFGGLKLAKIFRVSSELEAKGLDIPKHGEPAYPTEAYGHGWEEK

*Lottia gigantea* 2

>gi|676477210|ref|XP_009061039.1| hypothetical protein LOTGIDRAFT_126447, partial [Lottia gigantea]

MDEGNFTYHGSRNLKIRSHDDATWILTSAFIIFTMQSGFGLLESGSVSAKNEVNIMVKNAVDVICGGLTYWMFGYGFSFGDDEPYTNPFCGWGKFFVKASEEELGWVYSKFFFQASFATTATTIVSGAMAERTKLESYIVFSMLNTFVYCFPAHWIWGPKGWLQEMGVIDIAGAGPVHMVGGLSGLVATMVLKPRHNRYKKKKMPQMGSPTNALLGMFMLWWGWLGFNCGSTFGISGLKWKLAARSAVSTISASTAGGVVGLLSSYIVKKRTFDVAYLINGVLGSLVGITATCAMVHPWEGLVIGLIGGSVTITGVALLDRFHIDDPVGCVGTHALGGAWSMLAAGLFARRDTFAKELHLDAVQNGLLNGGGFYQLGVQTLAVVTLTVWTLFTSFFFLKIIDLVMGLRVPLEEEILGADIVEHGI

*Lottia gigantea* 3

>gi|676477213|ref|XP_009061040.1| hypothetical protein LOTGIDRAFT_93771, partial [Lottia gigantea]

DDATWILTSAFIIFTMQSGFGLLESGMATRRNEVNIMMKNAIDVIFGGLLYWAFGYGISFGTSKGSNGFTGWGSFFLDPDDDTMGTEFSKFTFQASFATTATTIVSGAMAERTKLHAYIIFCLTNTLVYSFPAHWVWADNGWLKQLHVVDVAGVGPVHLVGGVTALIAAVMLKPRYGRFDSKQQLKSSSPVNSLLGLFILWWGWLGFNCGSTFGISGGKWKLAAKAAATTIMASCGGGITSFIISYLFHNKKVNVRIITNGILGSLVSITGICAVTYCWSAIIIGSIGAIVEASGEALLVKLKIDDPVGATSVHFFCSIWGLLCCGLFARFDNIQMFSAYTGLFLGGGFELLGIQCLAIASIIGWSAIISYINLKLLDFVIGLRVDVEDELLGADVSEH

*Lottia gigantea* 4

>gi|676420862|ref|XP_009043740.1| hypothetical protein LOTGIDRAFT_102290 [Lottia gigantea]

MEVSNNSSLSYEQTGGLGPSGPITWDDATWILTSSFIIFTMQSGFGLLEAGSVSGKNEVNIMMKNVVDVVFGGVTYWAFGYGFSFGQDEGSNPFCGFGSFFVDTHGDDMGLVYSTFVFQLSFATTATTVVSGAMAERTKLMAYMIFSVFNTVVYCFPAHWVWGHNGFLRDLGVVDFAGAGVVHLVGGASSLVAAAFLKPRIGRFDEGKTAPPMNNPAGAIVGMFMLWWGWLAFNCGSTFGISGGKWKLAAKSAVTTLISSFSGGIVGIGLSFVVYKKKYDVAYMINSVLGALVSITACCALVRPWEALVIGMVGGFCTIFVIKITEIAKIDDPVGAIGVHGGGGLWGLIAVGIFAAEDSLEQLTYGRAGLTHGGGFYLLGVQTLCIVCEVFWSGLVTFIILFAIDKIIGLRMSAEDELLGSDLVEHNVGEWPNTNSDSKEENEPKEKIEDSDKLENRRHSLLIGDSGWSKATRLYRDKRNKVRISSIKEQLQSSSTSETSTNRTLQVSLHL

*Nematostella vectensis* 1

>gi|156378092|ref|XP_001630978.1| predicted protein, partial [Nematostella vectensis]

IGALGYWLFGYAFAFGTKSNAFIGEWNFALSYLPTNMYSSWFFQFVFAATAATIVSGAMAERTEFKTYLIYSIFLTGFIYPVVSHWGWDANGWLKKGVYYNDGNRTEHDFAGSGVVHVVGGTCALVGAAILGPRIGRFVNGEPVTIPGHTVPMTALGGFILFMGFLAFNGGSQGSISNAGDAETVSVAVVNTFISGAAGAVTSLLIKRIIPGAGKNWSLLTTINGALTGMVAICAGCDVVHPYGAFVIGLLAGVTYILWSAALLKMKIDDPLDSAPVHMGGGTWGLLAAPLFNHKTGVLYRWSTLSFRLWGWNILGLVSIMAWSAACAGIMFGFCHLIGQLRVSEEIEIRGLDVPKHGEPAYPVVSYGDGWNK

*Nematostella vectensis* 2

>gi|156361112|ref|XP_001625364.1| predicted protein [Nematostella vectensis]

MAGDTVDLQSLKQDLESYKANGDQKFLIIMGCLVFFMQCGFAFLEAGSVRAKNTTNILIKNVLDVFIGALGYWLFGYAFAFGAKSNSFIGQWNFALSYLPTSEYSTWFFQFVFAATAATIVSGAMAERTEFNTYLIYSIFLTGFIYPVVSHWGWHDNGWLKKGVYYTDGNITQHVYYQDFAGSGIVHVLGGTCALVGAAIVGPRIGRFVNGDPVTIPGHTVPMTALGGFILFMGFLAFNGGSQGSISNPGDAETVAAAVVNTFISGAAGALTALFIKRIIPGAGRNWSLLTTINGGLTGMVAICAGCDVVYPYGAFVIGILAGVTYILWSGALLKMKIDDPLDSAPVHMGGGTWGLLAAPLFNPKTGVLYHWSTLSFRLWGWNLLGLIAIIAWSSCCAGIMFGFCQLIGQLRVSEDIEIKGLDVPKHGEPAYPVVSYGDGWDTEEALGPEGFLANYGRGKNKKSASYEPVETVAMVKLDENGASADNVEQGP

*Nematostella vectensis* 3

>gi|156406743|ref|XP_001641204.1| predicted protein [Nematostella vectensis]

MQCGFAFLEAGSVRSKNTTNVLIKNALDVFIGAVAYWLFGYAFAFGSESNSFIGYSNFALAYVPTDKFSRWFFRFVCAATAATIVSGAMAERTEFKTYLIYSLFLTGFIYPVVSHWGWHERGWLKKGITTIRFSFQDFAGSGIVHVVGGTCALVGAAILGPRIGRFVKGKPVTIPGHTVPMTALGGFILFMGFLAFNGGSQGSISNPGDAEIAAVAVVNTFISGSAGALTALFIKRIIPGAGKNWSLLTTINGALTGMVAICAGCDAVYPYGAFVIGIFAGVTYILWSAIILKMKIDDPLDSCPVHMGGGAWGLIAAPLFNNKTGVLYQWNKLSFYIWGWNILGLVVIMAWSAGCAIIMFGSCRLVGQLRVPEDIEIKGKPSIITIIME

*Nematostella vectensis* 4

>gi|156406997|ref|XP_001641331.1| predicted protein [Nematostella vectensis]

MAELNASLLMSKVQSDLEILKSNNDQIFLIVMGMLIFFMQCGFAFLEAGAVRSKNTTNILIKNVLDGFLGALAYWLFGYSFAFANESNAFIGHWNFAMSYLPLNLFSKWFFQFVFAATAATIVSGAMAERTEFMAYLFYSCLLTGFIYPVVTHWGWDGNGWLSKGLNYDDGGVTVNVPYQDFAGSGIVHVVGGTCALVGAAILGPRIGRFVNGKPVTIPGHTVPMTALGGFILFMGFLAFNGGSQAQMSQAGDAEAVALSVVNTIIAGAGGAVTALFIKRIVPGAGKNWSLLTTINGGLTGMVSICAGCNAVYPYCALVIGILGGMTYVLWSAAILKMKIDDPLDSCPVHMGGGVWGVLAVPLFNYKTGILYRWNKYSFYAWAWNIVGLLAIMAWSAGCAALMFGFCHLIGKLRVPEDIERKGLDIPKHGEPAYPVVSYGDGWNMEEYQLGPGGFVDKSRHVNGNSSEQGPVEAIAIGKLDGDKRTNGDSSPPGRVNTGF

*Nematostella vectensis* 5

>gi|156372933|ref|XP_001629289.1| predicted protein, partial [Nematostella vectensis]

WDDAIWILTCSFIIFTMQSGFGLLESGMVSRKHEINILVKNIADVLFGGLAFWMFGYAFSFGKSHGTNPFIGFGDFFVDADGPNMGLTFSKFFFQASFSTTATTIVSGSMAERVKLESYCLFSFMNTIIFCLPAHWLWAENGWLKNLGAIDEAGACTVHLVGGVTGLTATIMLKPRSGRFEEGEDKSCNDMGSPTTALLGLFMLWWGWLALNCGSTFGVTDGKWKVAARAAVMTINASVGGGTTAILLSYFTKARKVDVKLLIRGILGSLVSISGLCGWARPWEAILVGAVGSLIACYGVSLLERVRIDDPVGSVSIHMFPAIWAMVAAGIFLEPDASAKRPGGLVQTGSAYTLGVELLATVVVSVWCALMATVILFFIDKTIGLRVSYQEEVLGADIVEHSIGGYSKARRGAVSQE

*Nematostella vectensis* 6

>gi|156382289|ref|XP_001632486.1| predicted protein [Nematostella vectensis]

MANNTTNTTLTPPPMEYPMNLRPDDATWILTSAFIIFTMQSGFGLLESGMVSRKNETNIMTKNAIDVIYGGLAYWIFGFAFSFGQPISNAFCGLSYFFADADESNMGMFAKYFFQLSFATTATTIVSGAMAERVSLKAYILYSFLNTLSYCFPAHWVWAGDGWLARMGAIDIAGCGPVHLVGGVSGLVATLFLGARTGRFNAKGELVEKPMGSPTNVLLGTFMLWWGWLGFNCGSTFGISGGKWKLASRAAVVTINGSIGGGIFATFYSYIAHANKLMVDVFVTGILGGLVGITAICAVVRQWESLLIGFIGGMIACVGDALVKKLRIDDPVGCIATHGMAGIWGLLAVGLFVEVDNLENFSAEFGVFKGGSWHLLGIQLLVVVSIGAWAAVTNFILLYVIDKVVGLRMPLAMELDGADIWEHGIHLQFCQYEENNAEERESPAVSTRCHAPSVMQNNPAVSTRCHAPSVMQNNFITGCINENVISMEDFEESKPGGVGQVPRGASTTALVN

*Nematostella vectensis* 7

>gi|156364369|ref|XP_001626321.1| predicted protein, partial [Nematostella vectensis]

MNKSNLTSASPPADQRDIIVPDDATWILTSAFIIFTMQSGFGLLEAGMVSKKNETNIMVKNAVDVIYGGLSYWLFGFAFSFGEGEGTNAFCGVGYFLTDADEYEMGDVFAKYFFQLSFATTSTTIVSGAMAERVNLKAYTLFSFINTLSFSFPAHWIWAKTGWLKKMGAIDVAGCGPVHLVGGVSGLVATLMLKPRTGRYDDQGRPKPLKMASPTNVLLGTFMLWWGWLGFNCGSTFGITGGKWKLASRSAVVTLNGSIGGGISGMVYSYLFHKNKLLIDTFATGILGGLVSITAICAIVRPWEAILIGAIGGAIACGGCSLLNRLRIDDPVGCVPTHFFASVWGLIVVGLLVEKDSLMDLSKEYGVLKGGSWRLLGAQLLAIVCISVWSAVMTFVMLCGVDRVVKLRMPLIMELEGADKWEHGI

*Nematostella vectensis* 8

>gi|156401043|ref|XP_001639101.1| predicted protein, partial [Nematostella vectensis]

DDATWVMSSAFIIFTMQSGFGLLESGMVSRKNEVNIMVKNVVDVIFGGLSFWAFGYAFAFGDSPSSNAFCGIGRFFTDATQPEMGTIFSKFFFQLSFATTAVTIVSGAMAERTKLESYCLFSSLGILVYCFPAHWVWAENGWLATLGAVDIAGCGVVHVVGGVTGLVATIILKPRIGRFNCSKPLSMGSPVTAILGLFVLWWGWLGFNCGSTFGITGEKWQLAARAAVNTLSASIGGGIFAFSYSYARYNRKVDVSYVVVGILGGLVSVTGICPLARPWESILIGTIGGVLSCFGRNLIQKLKIDDPTGCLAAHILSGIWGLVSVGFFTEVDNLEHFSTINGVLKGGNGWLLGIQLLTVVALIAWSAAVSFILLMILNKLVGLRVTEEEENLGCDAVEHEMDYRKTENTKQMKAIIGLNMFLNSKI

*Nematostella vectensis* 9

>gi|269785089|ref|NP_001161500.1| ammonium transporter-like protein [Saccoglossus kowalevskii]

MDTNMTVLGIELESVSANLSALQINADQFFLLVMACLVFFMQCGFAFLEAGSVRSKNTTNILIKNVLDVWLGAFAYWAMGYAFAFGGGNAFIGYRHFFFEGFSRSLMSHWFFHFVFAATAATIVSGAMAERTEFAAYLIYSFLITGFVYPVVSHWAWADNGWLLVGPGNGISYQDFAGSGVVHVVGGMAALVGAVILGPRIGRFENGKPVTGLSGHTVPLVALGGFILMFGFLAFNGGSQASISQPGDGYVVAIAIVNTVISGSLAALTSLTIRRMGLSGGGQWSLLTTINGALTGMVAICAGCCCVYAWGAAIIGVLSGIIYIVVSWLVLKAGIDDPLDAVAVHMGGGVWGVLSAPILAVDTGIVFNISSPLSWEALGWNFAGLAAIMGWTAIISILLFGVMKFAGILRVEATLELKGLDIPKHGEPAYPIESYGHGWGSMGMTLLTAPVNGTETSPVSGVETVRMNGIDNQALVMDHPTLRETTPVKGSPKTCRSIGVMVNMEVENATDQLEVDETVSTFM

*Strongylocentrotus purpuratus* Amt1

>gi|72171193|ref|XP_781954.1| PREDICTED: putative ammonium transporter 1 isoform X1 [Strongylocentrotus purpuratus]

MASNESDLVVMQLQAEFDYLKENTDHFFLVVMSCIIFLMQLGFAFLEAGSVRSKNTTNILLKNMLDVCIGAVAYWGVGYAFAFGKSSNPFIGYRNFFFEDMVPSDYSHFFFHFVFAATAATIVSGAMAERTEFAAYFMYSSLITGFIYPVVTHWAWSSDGWLGNSGIEIADGTYLAFKDFAGSGVVHCVGGTAALVGATILGPRIGRYDANGKLVVIAGHTVPMVSLGGFILFFGFFAFNGGSQGRISGEGDGEVVALAIVNTIISGAFAALTAMICRRVGFSGSYWSLLTTINGGLTGMVAICAGCNVVYAWGAAVIGTIAGVTYILWSSAVIRMRIDDPLDAVAVHLGGGFWGVLAVPIFDMNDGILFNIKSRVPWMHFAWNLLGVVAIFVWTALLSFFMFGIFRCLKILRVDPEIEKKGLDIPKHGEPAYPLASYGNGWEDESVIDASPVRPPPSYKMNGNSHDNKAYSLGETQNSVDHEHIQIDDTPRVQFD

*Strongylocentrotus purpuratus* Amt2

>gi|115965790|ref|XP_001181765.1| PREDICTED: similar to ammonium transporter 2, partial [Strongylocentrotus purpuratus]

IGAVAYWGVGYAFAFGKSSNPFIGYRNFFFEDMVPSDYSHFFFHFVFAATAATIVSGAMAERTEFAAYFMYSSLITGFIYPIVVHWAWSSDGWLANGPSGIGYQDFAGSGVVHCVGGTAALVGAAILGPRIGRYDENGKSKVISGHTVPMVALGGFVLFFGFFAFNGGSQASISVEGDGTTVSLAIINTIISGACAALVAMIIRKIGFSGSYWSLLTTINGGLTGMVAICAGCDAVYPWGAAIIGAVAGATYMMWSALMVAVKVDDPLDAVAVHLGGGIWGVLAAPLFDMNDGVVFTFSATSWQSFGWNLLGVFVIVVWTSVLSLVLFGLMQACGVLRVTPEMELKGLDVPKHGEPAYPIESYGSGWESNQTNNGNGHQIGQRQEYTNAAFEGAVAINNPSYTDKENGENDLPL

*Strongylocentrotus purpuratus* Amt3

>gi|115812241|ref|XP_795931.2| PREDICTED: putative ammonium transporter 3 [Strongylocentrotus purpuratus]

MNTTTVIWEEENLTTTESPTAYRATTWDDATWILTSAFLIFTMQSGFGLLESGSVTLKNEVNIMVKNAVDVLFGGISFWMFGYGLSFGVDKGSNAFIGIGDFFVDSKMMDAEMGHLFAHFFFHASFATTATTIVSGAMAERTKLEAYIVFSFVNTFVYALPAHWIWAPNGWLHAMGVVDISGAGPVHLLGGVTGLVATLMLGPRHGRFGEIEERPVQSSPINTLLGLFMLWWGWLGFNCGSTYGVAGQKWILTIRSAVSTILSSISGGITGITLSYLFLNRRFNIPWLINSVLASLVSITAYCALAHPWEAIIIGAVGGVIGCASTPFIEWLKIDDPASVISVHFFPAIWSLMAVGIFGQIDTLGDFNRHNGLIHGGGFWLLGVQLLSVVCLVVWTAITSFIFLFALKVTVGIRVSLHEELIGADVVEHGLHGNYDKRSGELTDQEGVVILRIDRSTPHDYELSLAALRRRVKLFGGTVFQRQWTGGAHTLDVPSKKSTSKKYECDSFQERQDESIRSDGPAVHVHFLNAALEGRSLSVNGGIGNER

*Trichoplax adhaerens,* 1

>gi|196015992|ref|XP_002117851.1| hypothetical protein TRIADDRAFT_61877 [Trichoplax adhaerens]

MSTSGILIASNVTSTKNPWQLNTLEYNVIGIFHIVTGLIVFFMQTGFAFLEAGSVRSKNTTNILIKNVVDMLIGAISYWAVGYAFAFGDGNTFIGTKYFLLINLPGPGYSFWWCNYVYAATAASIVSGAVAERIKFTSYLVYSAVITSFIYPIASHWAWDPHGWLMIGVPGASYQDFSGSSLIHGVGGMCALIGALFLGPRLGRFDKNGNPKNLPGHTVPFVALGGFILAFGLITFNVSSYFRVNGSLDAPKIALIGINSILAGSGGAIVALFTKRLHPKLSNHWSLLTCINGLITGIASVCASCNSIYPWAALLIGAIAGLTFMVWSWAILKFRIDDPLDAISVHLNAGIWGMIAQSLFNFKTGLFYRHMDKTSWLQFGWNMLGLLAILTWTSATSGLMFLILKKTGYLRVSEEIEIKGLDVPIHGEPAYPRISYGSGWGIESDVEMDTSVLMLLPSDNEDNEARVVKSQN

*Trichoplax adhaerens,* 2

>gi|196015994|ref|XP_002117852.1| hypothetical protein TRIADDRAFT_33117 [Trichoplax adhaerens]

MDDLQVESTAAPTNTTTHPYNVVTNENLNEVFHVIIGMIVFFMQAGFAFLEAGSIRSKNTTNILVKNLMDVFIGAVSFWAIGYALAFGKGTPFFGTEYFFLIGLPLTGYSYWWYHYVYAATASTIVSGAVAERTAFGGYLIYSSIITAFIYPIVVHWAWDPQGWLLYGVGKDFAGSSVVHCVGGVTSLTGAFILGPRIGRFDENGKPKTIPGHTVPLVALGGFILIFGLFGFNVGSQFRVTFYGGGPVAALIAVNTAMACSGGGLTALLIKRLHPKIGGNSWSLLVCLNGSLAGIVSICASCNAVYPWAALLIGAIAATAYVCWSEIFLRCKIDDPLDAAAVHMGAGIWGVIAQPIFNFKTSIFYTGFTALSWARFGWNLLGLGCIILWTGLTSGLMFYLLNKFGMFRVSEEIELKGLDLPIHGEPAYPRAAYGSGWGMDDDYDVEESIILLKQRKTSVPEQDQEEKE

*Trichoplax adhaerens,* 3

>gi|196002111|ref|XP_002110923.1| hypothetical protein TRIADDRAFT_1344, partial [Trichoplax adhaerens]

DDAGWLLASGFIIFTMQSGFGMLESGLVSKKNEVNIMVKNTVDVIFGGISFWMFGYAIAFGIDTVPGRNNPFTGIGNFFVDGHVNSSGWLYTNFFFQLSFSTTSTTIVSGAMAERTKLDSYCVFSFFNTLVYAFPAHWIWDSKGWLRQMGVVDAAGGCAVHIVGGFTGLIATLILRPRIGRFSSNYQPEMSSPTNAIVGMFMLWWGWLGFNCGSSFGVTGHLWKFSARAAVTTMMSSIGGGLYAIIFSYIAFKKKIKINHMINGILGALVAITPCCMVVDAWASIVIGIIAAACSCHGATLLARIRIDDPVDCVATHGFCGMWGMLAAGLFAQKELTDQFSIYDGLFYGGGMHLFGIQMLAVVCVTIWTLVTSFIVLLLIKVSMGIRLTEEEEHLGADAVEHAFD

**Fungi**

*Bactrachochytrium dendrobatidis* (BROAD INSTITUTE)

>*Bactrachochytrium dendrobatidis* BDEG_00136.2 conserved hypothetical protein (Transcript:BDET_00136)

MSVMVSDPGTSSIGFVIACTGLVFIMIPGVGLFYSGLARSKNALSLIMLSMLSMSVVTIQWVLFGFSLAFSDTGSAIIGNFAMGGLNSVGMDPIPVVATFIPGILFAVYQMQFATITVALVYGSVVERIRLVPALVFAFVWTTVVYDPIAYWSWANRGWVRNMGCLSTTALNQTPCGIGSLDFAGGGPVHVASGFAGLAYCLVVGKRRSIGPGSEKVEFLPHNITNVFIGAALLWFGWFGFNGGSALAATPRAAMAVTVTTISAATGSLAWTIMACLNHNRLSGIGFCSGAIAGLVAITPSAGYVAPWAALIIGFLSGVSCYYATRIKDAFGFDDSLDAWGVHGVGGILGSLLTGVFAQKKIAALDGASIDGGWIDGNWIQMGYQISGTVSIAVWSFVGSLLILLVIDVIPGLSLRPTPEDEDAGADYSEMGETCHSNESTRIINGTNPSLSDVSANRSSTHTLRVGTEVF

*Cladonia grayi* Mep1a

>gi|338808076|gb|AEJ07740.1| ammonium transporter mep1A [Cladonia grayi]

MSSTIAAVTSGAAQKTAQATAAIASASASVNPVAPPWLDPGHNAWQMTAGTLVALQSIPGLVVLYAGWVKHKWSINSAFMAFYAFAAVLVCWVVWGYQMAFGTQMLPLVGYPAIAVDMGFELEDSNLPTADLTQAFPQASMVYFQFVFAAITLVLIAGSCLCRMNFIAWMIFVPLWLTFSYTIGAFSIWGGGFLFKYGVIDYSGGYVIHLSSGTAGFVAAYWIGPRLPADRVDAKPSNITLMLVGAGILWTGWNGFNGGDPYAASPDAGAAVLNTNIATATSLLTWTIMDLIFFKKPSVIGAVQGMITGLVAITPAAGVVAGWGAILLGLGSGTVPWVSMNILGRTKIMRQVDDTLDVFHTHFVGAIVGGVGTGLWATPKGCAAFGITNPGGAVFGNGRQIGLQIVGALFIIGWNIVWTSLIMCFIKYVCRVPLKMSDEMLEIGDYAIHGEEPYTFEYYNRNYRRVAGFEHPGQMTDEEKAIREVRASKDFRRHDGSSGDGSHDAVIMGQDPNAGVHKAELVGDHSKKTE

*Cladonia grayi* Mep1b

>gi|338808461|gb|AEJ07739.1| ammonium transporter mep1B [Cladonia grayi]

MATNPPWLDTGDNAWQMTAATLVGLQSVPGLMILYAGIVKSKWAINSAFMAFYAFASVLICWVIWAYKIGFGKHMLPFAGIPGPALSMEYQLTQAILPTSGITANFPMSTMVYFQFVFAAITLVIIAGALLGRTNFKAWIIFVPLWLTFSYCVTAYSIWGGGFLAVLGVIDYSGGYVIHLSSGTAGFVAAWWIGPRLDADREDNRPNNIGSVLVGAGILWIGWNGFNGGDPYAASADAGVAVLNTNICTAMSMLCWTLCDVLYYGKPSVIGAVNGMICGLVAITPAAGVIAGWSAIALGLGSGTVPWASMNIFGKKVAFMRSVDDVLGVFHTHFVAGIVGGCATGIFATISGCAAFGITNPGGAIEGNGVQVGYQIAGACFVVGWNIVWTSLILMFIKYVLRIPLRMSEEDLLVGDYACHGEEAYVFGENSSAYAEGALRGKKIEGIAMGEDKGGIIMGKNPDPELEGSSNGSHGIQREEAREIKHD

*Cladonia grayi* Mep2

>gi|338808078|gb|AEJ07741.1| ammonium transporter mep2 [Cladonia grayi]

MASGPVLDGIDLGALNYTEQVPYNGTIPTGGDSLTNDLNVYYDAGDIAWMLTSTALVLLMIPGVGFFYSGLARRKSALSLIWLSMMATGVTSFQWFFWGYSLTFSHSAGRFIGDLANIGFRNTLAAPSVGSHKIPDLLFAVYQGMFAAITVALAVGAVAERGRMLPCVIFMLVWSTVIYDAIACWTWNPAGWVAHMGGLDFAGGTPVHISSGSAALAYSLMLGKRRGHGTHELNFRPHNVTQIIIGTVFLWVGWFGFNAGSALSASMRAVMAAVVTNLAASVGGVTWCLLDYRLEKKWSAVGFCSGVVAGLVAITPGSGYVPAWSAVVYGVAAGSACNYGTKIKFLLGIDDALDIFAVHAIGGLVGNLCTGFFAADYIAHLDGFTEIQGGWLNHHWIQLAYQLADSVSGFAYSFGGSCLILFVMNLIPGLRLRASEEAEVLGMDDAEIGEFAYDYVELTRDVINGDALVDEGESKYSGDVDSPIGGQGREKGFSSSTDETALRGPLR

*Cladonia grayi* Mep3

>gi|338808080|gb|AEJ07742.1| ammonium transporter mep3 [Cladonia grayi]

MTSYPAPAGYAPSYSWIGAPTSFNGTNSLTGGDSSVENLNQWFAPGDQAYIIVASAMVVVMIPGLGFLYSGLARRKSALTMIWVCMASFSVITFQWYLWGYSLAFSAQGMSGFIGDLNHFGLINTLGAPSPGSPLIPELMYSFYQLQFCATTAAIVAGAVAERGRVLPFMVFIFCWATLVYCPIACWAWNINGWGFNYGVMDYAGGGPVEIGSGMSALAYSLVLGKRQEKMMANFRPHNVSLIVLGTTFLWFGWLGFNGGSSFGANLRAVQACWNTNLCAMFAAASWTILDWRXARKWSMVGWCSGAISGLVAATXASGYCTAWGCVVLGVVSGIVCNYATKVKYWLRIDDAMDVFAEHAVAGMLGLMANSLFGATYIIGLDGVNTGIITGGWINHNYRQFYVQLAYILAATGWAFAISLLLAFLINLIPGLHLRASEEAELMGMDDDQLGEFAYDYVEVRRDYLAWTPTRKDMQTEDGIHVPAHERHGISQQKDMLLGKDVDGSSGKSGEEHTGIAGDRHAAAYEKQQEGQAHG

*Cryptococcus neoformans* var. *neoformans* Amt1

>gi|58258403|ref|XP_566614.1| ammonium transporter MEP1 [Cryptococcus neoformans var. neoformans JEC21]

MVNITYGALLSSSDGAVHFEPLGTDIISTLAGQPTAFDPGDIAWVLTCSALIVFMLPGLGYLYSGLARRKNALSMLFLSLVSLGIVSFQWFFIGYSLVFSETGGSFWGDGRNIGFRQVLERPIPEANGKLPEIVFATFQLMFACLVPAVLLGAAAERSRILPAMIFMFCWTTLVYDPLAHWIWSANGWANKWGVLDYAGGVPVEIASGTAGLAYSYFIGKRRGYGTDRVLFKPSNVGNVVLGTVFLWVGWLGFNGGSCYCASLKAALAIFNTNLAGSVGGVVWLIMDFRLERKWSMVGYCTGAIAGLVAITPAAGYVGAPAAALIGLVSAAVSNLATRLKVTMRVDDPMDIFAVHALAGIVGVLMTGLFAQSSVAANDGFSDIDGGWLDRHYVQLGKQIAWACVGMAWTFVVTYAIMFFINLIPGCHFRATDEAEIVGMDEVELGEYVADYAFHQRDLEGEYEAHTLSQCPSATKLHLQEFRHNKDGESSSSSQPKELPPTMPRGGAVVGDLDGDTAVESQHSRSRSRGRSQHRVRIEKEGDNEAMEMSDVRHTKTSDQRQEGSNLEGSYMDHRE

*Cryptococcus neoformans* var. *neoformans* Amt2

>gi|58259902|ref|XP_567361.1| ammonium transporter [Cryptococcus neoformans var. neoformans JEC21]

MVNVTYTDSSSDMIYTADDGTQYLYNLGDMSFVIAAMALVWIMVPGVGLFYSGLLRRKNALSMIFLSMAGVAVGSFQWFFWGYSLAFSDTGSKYIGDLRYFGLKGVLAEPSAGSDRIPALLFCVYQCMFCLITGVLAIGGFAERSRIGPVMVFLFCWLTLVYCPLACWTWNPNGWSFVMGGLDFAGGTPVHISSGTASLAIALYLGKRRGYGTERLAYKPHNTAFVVIGTVFLWFGWFGFNGGSALSANLRAVQACIVTNLSASVGGLVWMFLDYRLERKWSAVGFCSGAISGLVGITPAAGYVGSPAALAIGAITAIACNFATKLKFLIGVDETLDVFASHGIGGMVGCFLTGLFAQGSVAGFDGITDIPGGWVSHYWIQAGYQMADLTAGFAYTFVMTTIICWLLHFIPGLRLRASEEAEIIGIDDAYLGEFAYDYVGTDPELRLHRIDSKPQFTSGDVIAEVSASNGHESSTTEKVDPHATGNAAAGGGRVDV

*Hebeloma cylindrosporum* Amt1

>gi|20501863|gb|AAM21926.1| ammonium transporter [Hebeloma cylindrosporum]

MVNVTYDDSGSLVAYTPDGTPIVYNLGDMAWIAASMALVWIMIPGVGLFYSGLLRRKNALSMIWTSLASIAVVSFQWFFWGYSLAFSETGNAFIGDLKYFGLKGVLDQPSIGSSRIPAILFCIYQLMFAAITAALAVGAIAERGRLGPLLVFIFVWSTIVYDPIANWTWNTNGWSFILGGLDFAGGTPVHISSGTAALAISIFLGKRRGYGTERLAYKPHNTTYVILGTVFLWFGWFGFNGGSALSANLRAIQACIVTNLAASVGGLTWMLWDYRIERKWSAVGFCSGAVSGLVAITPASGFVGAPAAVAFGVLGGTACNFATQLKFVLGYDDALDIFATHAIGGIVGNLLTGLFAQASVAGFDGITEIPGGWLDRHYIQLAHQLADSVAGFAYSFVMTTIILWVMHIIPGLTLRTTEEAEILGVDDAEMGEFAYDYVGIDQEIGHTLDTGLTATGGGREPDHAKAVSTSSVEEKSA

*Hebeloma cylindrosporum* Amt2

>gi|15042693|gb|AAK82416.1| ammonium transporter [Hebeloma cylindrosporum]

MVNVTYDASQPLVWTDAAGNTGVYSPGDIAWVLASTALVWIMVPGVGFFYSGLLRRKNALSMIYLSMMTVAVVSFQWFFWGFSLAFSDGASLFIGDLKYFGMKGVLEEPSIGSTRIPAIVFSVYQLMFAAITPMIAVGGFAERAHLGPLLFFVFVWSTLVYDPIACWTWNSKGWSFVHGSYDFAGGTPVHISSGTAALAISIYLGKRRGYGTEVLTYKPHNTTYVILGTIFLWFGWFGFNGGSALAANLRAAQACIVTNLAASVGGLTWMLWDYRIEKKWSAVGFCSGAIAGLVAITPGSGFVGSPAAVLFGFMAGTVCNFATQLKFFAGYDDSLDIFASHAVGGVVGNLLTALFAQASVAGFDGFTVIPGGWLDRHYIQLAWHVADSAAGLSYSFVVTTIILWVMHFIPGLRLRVPEETEIIGIDEADMGEFAYDYVGLETELKPHVYVRSTGTAYDSGRNSELKERSSA

*Hebeloma cylindrosporum* Amt3

>gi|15042695|gb|AAK82417.1| ammonium transporter [Hebeloma cylindrosporum]

MSNLATGQFDRGDISFIVVAGAMVFFMVPGLAFLYSGLSRRKSALSLIWAVAASNAVVIFQWYFVGYSLAFSSTATNGYIGNLRNFGLMKVEADPSPGSPLIPEILYSFFQMEFACVTAGILMGGLAERGRVFPAMIFIFFWMTFVYCPLACWAWSTHGWAFKWGVLDFAGGGPVEIGSGVGGLAYAWVLGRRNERELLNFRPHNVGLVGLGTFMLWFGWIGFNGGSAFGANLRAIFAIWNTMIAASFGGMVWCLLDFRIERRWSMVGFCSGTIAGLVAATPSSGYLRPWSSVVVGVLAGTLCNFATKVKFLLRIDDALDLFAEHAVGGVVGLMLNAFFAETAVIAMDDVTIGVRGGWVDHNWKQLYIQFTYVVATCAYSFVMTALVAKTVDLIPGLKLRSSPEGEMLGMDEVEIGEFATDYIELRRDVADCSTFGFTRGSNGYIPHAATNETHAQDTSSNDDPHHRIDEPTAPERLEPLTEKNECGTVTPS

*Neurospora crassa* Mep1

>gi|85112788|ref|XP_964410.1| ammonium transporter MEP1 [Neurospora crassa OR74A]

MSYSWFGAPTPFNGTTPNGGDSASENLNQWYQSGDQAFILVASCMVLIMIPGIAFLYSGLARRKSALSLIWVCMMSSSVVMFQWYFWGYSLALSSTATNGFIGNLRHFGLMNTLGAPSPGSNLIPELLYAFYQMQFVGVTAALVVGATAERGRVVPAMVFIFIWATLVYCPLCCWAWNVNGWAFKYGVMDYAGGGPVEIGSGVSALAYSWVLGRRNEKMMLNFRPHNISLITIGTVFLWFGWLGFNGGSAFGANLRAAMACWNSCLTAMFAAMTWTLLDYRLAKKWSLVGWCSGTISGLVAATPASGYIPPWASIILGVVTGIVCNFGTKIKFMIGIDDALDIFAEHALGGVVGLIFNGFFAADYIIGLDGVNQEIPGGFLNHNYKQLYIQIAYVCATMGYSFVMSAILAKGVDMIPGLHLRASEEAELLGMDDDQHGEFAYDYVEVRRDYLAWTPAEKEQRQDGDIIVPQHGIEGHQELLSSSRHGPTMSELPHHEEEDPREKRGSTSE

*Neurospora crassa* Mep2

>gi|85104138|ref|XP_961677.1| ammonium transporter MEP2 [Neurospora crassa OR74A]

MSSGPVETYEGVAGVANNGGNPLLEDLNRYYSAGDFGWIMTCTALVLLMVPGVGFFYSGLARRKSALALIWLSLMSIAIVGFQWFFWGYSLAFSHTGSAYIGNLSNFGLMKTLAQPSVGSGKIPDLLFCLYQGMFAAITPALAIGACADRGRMLPALVFMFIWTTIVYDPIAYWTWNGNGWSFKMGGLDFAGGTPVHISSGAAALAYSLMLGKRNGYNKVNGLPYRPHNVTHVVLGTVFLWVGWFGFNGGSALAANLRAVMACIVTHIAACVGGLTWVLLDYRLERKWSTVGFCSGAIAGLVAITPAAGYVPPWSAFIFGVVGGISCNFATKLKFLLGIDEALDVFAEHGVGGIVGNLLTGIFAADYIAALDGVSIGDSAIAGGWVNHHYIQLAYQLADSVAGFSYSFVMTCVILFLLNLVPGLSLRVDHHQEDIGLDDDQLGEFAYDYVEVTRSLDVIPGLTAPNSTAGSISGRDPEKQV

*Neurospora crassa* MepA

>gi|85095497|ref|XP_960103.1| ammonium transporter 1 [Neurospora crassa OR74A]

MAEPEIVPVTEWPDWHANPNGGDPFTQNLNSPYDKGDLCWMLVCTALCWQITPAIGFLYAGMHRRKAALTMVLQSLFCACACGIQFWIYGYSLYQARTTNPILGDLSLAVFRNVLAQPSMANSDIPDILYAAFGFTFVSCTAMILAGAMLERGRLLPSMLFLLCWTTFVYYFIAYWEWNPSGWLYKLGVYDFAGSGPVHIASGFGALAWSLMLGPRVADSDVVDRKKLVHHKPHSPLLMCLGTVFIWFGWFAFNGGSTPNLSLRSIYAIVNTNLAGCGGGVGWVLIDYLYKRKFSLVGFCSGIIAGLVGITPAAGYVPVYVASLIGFLTSATCYYAAKYKHLLSVDDGLDIFAIHGVGGYVGDLLTGIFADNFVPALDGYSGSSYAGGWWNRNFRQLGLQFAGATTAAAWSFVVSCILLFIINKIPGLHLRASEDSEIRGLDIKYLEDVDEEGFYMNECILHGRTPPRCSGSLKAPTSVTEPVGVEGGEKRD

*Neurospora crassa* 4

>gi|85107333|ref|XP_962355.1| ammonium transporter [Neurospora crassa OR74A]

MTVSPVLAAWEACDKTDTLFVLICTVICWTIVPTVGIAYSGYTWKRNALHAAMPAILTIATCSIQWFIVGYTLAYGEGGWFFGSLKHFFHKDVLSEPVGRIPAILFSEFQLVFEATVCAIAVGGFCERGRLLPVIPFIFLWATFIYCPLAHMVWGGGLLGENLGVLDFAGGTPVHVCSGATATAISIYLSYPLFRSRKSDVRTPTHIRIHRPGNSFFQLLSLIIIWGSWLAFDAGTTLALNFQSVMALCVTNLCAASGALTWNLITFYETGKWSVDSTFMGAISGLVMITPSAGFIDMPTACFFGIFGAVLCRQALRIKFTKFAHHWRWVDNGDTFATHCIGGVAATIMTGLFAQKELAAYGGVEVAGGVIFDGNIRQLWVQMVEAVVGFTWAFCGSYIIIALIDCVPGLEVLAVDKHISQGLDMNEVEEFLGNLEYPDEVDYAPTARGTISLE

*Saccharomyces cerevisiae* Mep1p

>gi|6321559|ref|NP_011636.1| Mep1p [Saccharomyces cerevisiae S288c]

MESRTTGPLTTETYDGPTVAFMILGAALVFFMVPGLGFLYSGLARRKSALALIWVVLMATLVGILQWYFWGYSLAFSKSAPNNKFIGNLDSFGFRNVYGKKFDEDAYPELAYATFQMMFSCVNLSIIAGATAERGRLLPHMVFLFILATIGYCPVTYWIWSPGGWAYQWGVLDWAGGGNIEILSAVSGFVYSWFLGKRNEKLLINFRPHNVSLVTLGTSILWFGWLLFNSASSLSPNLRSVYAFMNTCLSAITGGMTWCLLDYRSEKKWSTVGLCSGIISGLVAATPSSGCITLYGSLIQGIVAGVVCNFATKLKYYAKVDDAMDILAEHGVAGVIGLIFNALFGADWVIGMDGTTEHEGGWVTHNYKQMYKQIAYIAASIGYTAAVTAIICFVLGYIPGMRLRISEEAEEAGMDEDQIGEFAYDYVEVRRDYYLWGVDEDSQRSDVNHRVNNAHLAAERSSSGTNSSSDGNGEMIQSEKILPIHQEDPANR

*Saccharomyces cerevisiae* Mep2p

>gi|6324187|ref|NP_014257.1| ammonium permease MEP2 [Saccharomyces cerevisiae S288c]

MSYNFTGTPTGEGTGGNSLTTDLNTQFDLANMGWIGVASAGVWIMVPGIGLLYSGLSRKKHALSLLWASMMASAVCIFQWFFWGYSLAFSHNTRGNGFIGTLEFFGFRNVLGAPSSVSSLPDILFAVYQGMFAAVTGALMLGGACERARLFPMMVFLFLWMTIVYCPIACWVWNAEGWLVKLGSLDYAGGLCVHLTSGHGGLVYALILGKRNDPVTRKGMPKYKPHSVTSVVLGTVFLWFGWMFFNGGSAGNATIRAWYSIMSTNLAAACGGLTWMVIDYFRCGRKWTTVGLCSGIIAGLVGITPAAGFVPIWSAVVIGVVTGAGCNLAVDLKSLLRIDDGLDCYSIHGVGGCIGSVLTGIFAADYVNATAGSYISPIDGGWINHHYKQVGYQLAGICAALAWTVTVTSILLLTMNAIPFLKLRLSADEEELGTDAAQIGEFTYEESTAYIPEPIRSKTSAQMPPPHENIDDKIVGNTDAEKNSTPSDASSTKNTDHIV

*Saccharomyces cerevisiae* Mep3p

>gi|6325396|ref|NP_015464.1| ammonium permease MEP3 [Saccharomyces cerevisiae S288c]

MARGDGHLWTETYDSSTVAFMILGAALVFFMVPGLGFLYSGLARRKSALALIWVVIMATLVGILQWYFWGYSLAFSKTATNNKFIGNLDSFGFRNVYGKISDDSTYPELIYAIFQMMFMCVALSIIAGATAERGKLFPHMVFLFVFATLVYCPITYWIWAPGGWAYQWGVLDWAGGGNIEILSAVAGFVYSYFLGRRKENLLINFRPHNVSMVTLGTSILWFGWLLFNAASSLSPNMRSVYAFMNTCLSATTGGMTWCLLDYRSEKKWSTVGLCSGIICGLVAATPSSGCITLYGSLIQGIIAGVVCNFATKIKYYLKVDDSLDLLAEHGIAGVVGLIFNALFAADWVIGMDGTTKHKGGWLTHNWKQMYIQIAYIGASAGYCAVVTAIICFVLGKIPGVHLRVTEEAEALGLDEDQIGEFAYDYVEVRRDYYQWGVDTDALHTTCNGANSASETNPTEDSQNSSLSSATVSGQNEKSNNPKLHHAKEA

*Ustilago maydis* Mep1

>gi|71023613|ref|XP_762036.1| hypothetical protein UM05889.1 [Ustilago maydis 521]

MVNASYTPSGDLITYYDDQGTPTELIYNLGNMSWIIVSTALVFIMIPGLGFFYAGLLRKKSALSMIWMSMVILSVASFEWFFWGFSLAFSTSSSNKFIGNLDNFGLINVDIGPSTGGTGLPQLLFCIYQMMFAAITPVIACGAFADRARLGPVLIFAFCWCTIVYNPIAYWTWNVGKGWANVMGGLDFAGGTPVHISSGTAALAISIFLGKRKGYGTEQLAYRPHNVAYVILGTVLLWFGWFGFNGGSALGANLRAVQAMMVTQVATSVGGLTWMFWDWRLERKWSAVGFCSGAISGLVAITPASGYVGTPAAVAFGVLGGTACNFATGLKNLFGYDDALDIFAAHGIGGIVGNLLTGIFADNRVASFDGTAPIPGGWINKNWIQLGYQLADSAAGFGWSFVVTLILLFAIDRIPGCHFRSSEEDESLGIDLTQIGEEVMVMPLLHDFVRPEDGAEIHHATASTDLKFSPAHSEKDLEAARPQPTAV

*Ustilago maydis* Ump2

>gi|71020879|ref|XP_760670.1| hypothetical protein UM04523.1 [Ustilago maydis 521]

MVNITYGVLQAPDGSSATSSAIANGTDIIVSYGPNGESVASYSPGDISWVLTCTALVWLMIPGLGYLYSGLARRKNALHLLLLTLAALAVVSFQWFFWGYSLTFSQTSGAFLGNLRNFGYMGVLENPVPQAYNKVPEIVYAIYQCMFANLVPAIAIGAACERGRVWPFLVFTFVWTTLVYDVIACWVWNPTGWAFKWGVLDYAGGGPVEINSGITGLVISYYLGPRTGYGTERLLFKPHNVSYIFLGTAFLWFGWIGFNGGSVFAANLRAAMAISTTNLAASSAGLTWMFLDWRLERKWSVVGFCTGAIAGLVAITPAAGFVGMPAALLVGVVSSAVSNFATVLKGPMRVDDVMDIFSVHALAGIVGTLLTGIFAQASVANNDGNTVIAGGWLDSNWIQLAYQLAYCVAVTCWCGGVTFAIMFVIDHIPYIGPFRSSEMGEVVGMDEDQCGEWAYDYAFINRDLEGNYKPDHGQTLDINEKMPNVHQTDSLRSETHADSSQDNSNSQLAAAEHQSPSTAEPAVEMKQA

**Other eukaryotes**

*Dictyostelium discoideum* AmtA

>gi|31323873|gb|AAP47145.1| ammonium transport protein A [Dictyostelium discoideum]

MVAGEIIKGVAAEITNGSSSSVVQKYLDCANQVAPDPGNTTWVLLSTILVLGMMPALAFFEAGLLRSKNTLSIITQIMSGIVVLTVMWQAFGYSLTFGPDQKGIIGNLDHAFLINVSYDDCSPNAPNIPAAAYAFFMMMFANITPLLMTGAFAERVKFKAFIALTVAWEIIVFYPVAHWIWGGGWLHKYFGVLDFAGGIVIHTSAGVSALVIALYVGRRKDFEKYGGEFPPSNLPLATIGAALLWMGWFGFNAGSALAAGNIATSAVASTQIGGSFSAIVWIILSAAKGKPNTVSVINGVIAGLAGITPASGYINSQYSIGLGICLGLASYYSVVLLKHKLHIDDALDVSSVHGLTGIIGSLAIGFCAELSVNPNGANGAFYGNPKLIGTQLLGVVSVAVWAAAWTWVLLKIIDATIGVKIDESEEELGLDLVEHGEFAYHNISLQGNENHYSSVINSHDFFK

*Dictyostelium discoideum* AmtB

>gi|13429834|dbj|BAB39710.1| ammonium transporter AmtB [Dictyostelium discoideum]

MEYLELNGTKEGLSAAVDDMWVLNATYLVFYMQAGFCMLEAGVVRAKNAKSIIMKSIIDTAIGSLLFWALGFGLAFGNADKASNPVIGTSHFFLINYQNLSFFAFQWAFCATSITIVSGSLAERVHVTSCLVYTIVMSAFIYPLSAHWVWSYNGWLRMIGFNGIIDFSGSIVVHIVGGCIGLVGTYLVGPRIGRFDSESGKPKPLPGHSITIYTLGAFIIWYGFYGFNTGSTLGISGGGIAIASRSAVTMTIIACASCATTLLAIKIKSGKYDVVKSVNSLLGGLVSSAAVCSLIDPWAAFIIGCVTSFVYLGCSHLLIKLRIDDPLDSSAIHLGCGIWGALSVGLFSTQENLSLVLKKSTNVYGLFFGGGFEQLGIQLLGIIIVMAWCFFCASILFTILKKFHLLRIEPTKELMGIDIDSAGGPAYQWDN

*Dictyostelium discoideum* AmtC

>gi|31323877|gb|AAP47147.1| ammonium transport protein C [Dictyostelium discoideum]

MEQFSTSSSESSDSSSEYSLEFYMDTSWVLDAANLVFFMQAGFGMLEAGMVRAKNTKSILLKNLINTAICAISYYCVGHSFAYGKVNPNSFVGFGNFFLMDYTHYAYWMIQWAYAATATTIATGAMAERLQLHCYILFTLVQTILIYPFVAHWIWSQNGWLFDLGIVDFAGGAVIHIVAGITGACGSFLLGPRIGRFNQESGKPKNLPGHSVVLMSLGAMILWYSWYGYTAGASLGMTRSRVLPASRVSVVVTLSGATGLITVLGIGKIFNGHYDLVKGINGLIAGLVSSTSSCAYIEPWAAIIIGFIGGIVYWFSSWALLNWLRLDDPVDSTAIHLFGGCWSLISVAFFATPGRVRNPDILLPGGIIYGGGISLLWVPLVGMVLAILWAGFLSGIFFFTMDYFGKLRVDVDSELAGLDNSNHGGRA

**Archaea**

*Archaeoglobus fulgidus* Amt-1

>gi|2649619|gb|AAB90264.1| ammonium transporter (amt-1) [Archaeoglobus fulgidus DSM 4304]

MSDGNVAWILASTALVMLMVPGVGFFYAGMVRRKNAVNMIALSFISLIITVLLWIFYGYSVSFGNDISGIIGGLNYALLSGVKGEDLLFMMYQMMFAAVTIAILTSAIAERAKVSSFILLSALWLTFVYAPFAHWLWGGGWLAKLGALDFAGGMVVHISSGFAALAVAMTIGKRAGFEEYSIEPHSIPLTLIGAALLWFGWFGFNGGSALAANDVAINAVVVTNTSAAVAGFVWMVIGWIKGKPGSLGIVSGAIAGLAAITPAAGFVDVKGAIVIGLVAGIVCYLAMDFRIKKKIDESLDAWAIHGIGGLWGSVAVGILANPEVNGYAGLLFGNPQLLVSQLIAVASTTAYAFLVTLILAKAVDAAVGLRVSSQEEYVGLDLSQHEEVAYT

*Archaeoglobus fulgidus* Amt-2

>gi|2648804|gb|AAB89503.1| ammonium transporter (amt-2) [Archaeoglobus fulgidus DSM 4304]

MVGRMCVAVLIVLLLVATAGADPNGAETLKENPELPVDFVWALICGFLVMFMQAGFAMLEAGFSRAKNVANVMMKNLMDFAVGSLAFFAVGFALMMGADWQGIAGTTGWFLAGESYDVSTIELWFFMLVFAATAATIVSGSIAERPKFSVYLVYSAVVSAVIYPIYGHWLWGGGWLSSSEFMVKLGGGYGALDFAGSGVVHALGGYIALAAVMLLGPRLGKYDSDGNPRAIPGHNLAFAVIGTFILWFGWFGFNAGSTLSAHELRVSIIASNTNLAAAAGAVTAMAITWLRNGKPDVGMTCNGAVAGLVAITAPCAWVQPWSSVVIGTIAGFIATYGYWWLEKRGLDDVVGAIPVHGFSGTWGLIALGIFADGSYGLYATESPLVTGLLYGNWGFFIVQLISAIVNFAWAFGTGFALFWILKKVIGIRVSPEEEMLGLDIAEHAAVAYPNFVCTETELPLAMKQGGGR

*Archaeoglobus fulgidus* Amt-3

>gi|2648802|gb|AAB89501.1| ammonium transporter (amt-3) [Archaeoglobus fulgidus DSM 4304]

MQSGDISWLLISTALVVLMTPALGLFYGGMVRAKNVLSTITMCFVALAIVSVQWILLGYTIAFGDDVSGLTGNLVFIGLRNVSMSDYGFVAFQMAFAAITLAIIVSALAERVKLSAFLLFGILWTTFVYDPIAHWVWGGGWLAGIGALDFAGGTVVHISSGFSALALALLVGRRTGYGEYNIEPHNIPMTLLGTALLWFGWFGFNAGSALEANDVALNAFLVTNTSAAMGALSWMFASWLKGKPGSLGVASGAIAGLVAITPAAGYVDPLSALVIGGVAGVVCYAAMLLRVRAGVDESLDAWAVHGVGGLWGAIATGIFASVGATGLVAGNAQQLLLQIIAALVATAYAFLVTLILAKAVDAAVGLRVSSQEEYVGLDLSQHEEVAYT

*Caldivirga maquilingensis*

>gi|501137207|ref|WP_012185740.1| ammonium transporter [Caldivirga maquilingensis]

MVYILSVVYIVVSSVLIVKAQSQQLSGYPSSSVPNWLDTGSNAWVLTAATLVGLMSVPGLMLFYGGLTKRKYAINTMMMVLYAFSIVLVLWVLFEYEESFGSPLLAIKGFGVLGKPIPILSSGLLGSQAVIPLAQQAPNITLSTLAYFQFVFAAITPALIVGALIERMNFKAWMLFVPLWSALVYGPVAYWLWGGGWLMRLGAVDFSGGYVIHLDAGVAAVIAAMLVGPRLNEERKLAPHNLTQVAAGLGLLWLGWNGFNGGDPYGVTIDASIAVLNTNLAAAMALLAWVILDEAVYGKVSLTGAASGAVAGLVGITPAAGYVNGLGALVIGVVSGAAAWASLNLIQYRIMPLRNIDDATGVFSTHTVPGFLGGVLTGVFADPRIADFIDPGLAGALYGNVYQLLIQVIAAMVVIAYSGLMTFAILKLVSKVTPLRASEWVLRVGDREMHGEVAYDEYAFPIQVGDVEVINGRSRE

*Picrophilus torridus*

>gi|499491622|ref|WP_011178262.1| ammonium transporter [Picrophilus torridus]

MSFDVSWLNTGNNAWMLTAATLVGLQSIPGLALYYAGMTKRKYMVNAMMMALYAFSAVLVVWVIAGYSFGFGTVPLLKIDGYYIFSMPVPVGSASFIAGQAIVGPERIALSIPNSTVIFFQFVFAAITPVLLMGGILERMNFKAWMVFVPVWSFLVYSPVAYWLFAGGWLNQLGAVDFSGGYVIHLDAGVGALAAALAIGPRIKEDMNLKANNLGLVMLGLGLIWLGWDGFNGGDPYGSGIDAAIAIINTNVATAVSMITWMLMDMKFFKKASLIGASVGAIAGLVGITPAAGYVNIYGAMVIGIATGIIPWIALYKIEPHFKIDDALGVFSSHAVAGIVGGILTGVLADPFITQYIDPGLRGAIYGDFYQLGIQALAAAVVFAYTFAMTIAILKIIGLFIPLREKEENLKLGDLAIHGEIGFEDSYQAVRK

*Sulfolobus tokodaii*

>gi|499287353|ref|WP_010978643.1| ammonium transporter [Sulfolobus tokodaii]

MEIQKTFPQNNLIKLLIILISFSIILLSLHSFGQTSSNTTAEIQSLNQSILALENHTADYPSAAVPSWLDTGSNAWMLTAATFVGLQSVPGVALYYAGLSKKKYAVNSALMVFYAFAAVLVVWMIAGYNAGFGHPALLSINGYGILGYPLPAWLGHYEASQTVFGPTGTPVDIPTSTYIFFQFVFAAITPVLLAGGVLERMNFKAWMIFVPFWSLLVYSPVAYWLFAGGWLNQLGAVDFSGGYVIHVDAGVGALAAALAIGPRLASERKLEAHSLPLVLAGAGLIWLGWDGFNGGDPGGATIDAAIAVLNTNIATAVSAITWMLMDMAFFKKPTLVGATSGAITGLVAITPAAGYVNGWEAMLIGIASGSIPWLSLYKFEPRLKVDDTLGVFSTHGIAGIVGGLLTGVFADPNVTQYVDPTLKGALYGNLYQLGIQAAAAAVVFVYDFAITFGLLKLIGLFIPLQAPPDTLAIGDYAMHGEVAYSELLATLPESQIKKEEIREEAQEEKEDPDEKKSK

*Sulfolobus solfataricus*

>gi|497674981|ref|WP_009989165.1| ammonium transporter [Sulfolobus solfataricus]

MKSWKFKALLIGQVILVMLLFSNILAHSQTTTNSSALQQLNQSVQSILNRTSSYPAAAVPSWLDTGSNAWMLTAATFVGLQSVPGVALYYAGLSKKKYAVNSALMIFYAFAAVLVIWIIAGYSFGFGYPALVSIHGYGIFGYPIPAWGGLFEASQTTYGPSGAHANIPTATFIFFQFVFAAITPILLAGGVLERMNFKAWMVFVPFWSLLVYSPVAYWLFAGGWLNQLGAVDFSGGYVIHVDAGVGALAAALAIGPRLASERKLEAHSLPLVLVGAGLIWLGWDGFNGGDPLGATVDAAIAVLNTNIATAVSAVVWMLMDMKFFGKPTLIGATSGAITGLVAITPAAGYVNGWEAALIGIFSGSIPWMALYWLEPKLRVDDTLGVFSTHGIAGIVGGLLTGVFANPAVTQFVAPGLTGALYGNWYQLGIQALAAVIVFVYDFAITFGLLKFIGLFIPLQAPPQELAIGDYAMHGEVAYSELLATIPESAKSKEQSVVLPKKEKEENSEE

**Bacteria**

*Acidimicrobium ferrooxidans*

>gi|506279599|ref|WP_015799374.1| ammonium transporter [Acidimicrobium ferrooxidans]

MIAALLPYASWLHPGDNSWQLTAATLVGLMSIPGLAVLYAGLAPKKWAVNIMTMAFVGFSAVLVVWVLWGFDMAFGSPIHLGSGLWGSFLGHPGLSTSAHAEQSQASIPLVDGLMPSFRFPQSALVYFQFVFAAITPLLFLGSVWGRMNLKAWLIFVPLWSTVVYSLDAFLLWGGGFWAQKGAVDFSGGYVIHLSAGISGFVAAAVIGPRLHRDREHERPHNLVYMAIGAGILWLGWNGFNGGDPYFAGADAATAVLNTNIATAAALLTWLFMDMSSKRLNRKPTFGGAINGMIVGLVAITPAAGYVNGLGALALGIIASFIVWWSFNVWSQKGLFRKVDDAFGVIHTHGVAGLAGGLLTGILADPHVIEYVGTGKTSSSSVTGLLYGHPGQLLLQLGAGLTVIAISAVGTFVILKLVGLVVPLRYPDEVLAVGDLAIHGEVVNPEDAGVEVLDEPTLIDPALRDHDPHGESEELRDLELE

*Acidithiobacillus caldus* Amt-1

>gi|254968360|gb|EET25921.1| ammonium transporter [Acidithiobacillus caldus ATCC 51756]

MARDRENFQPNNVLLMLVGAGILWLGWNGFNGGDPYAASRDAGAAVLNTNITTAMSVLTWTAMDIFYFKKPSVIGAVQGMITGLVGITPAAGVVDGFGAIAIGLATGIIPWFTMNILGKRLSFFRKVDDTLGVFHTHAVAGFLGGILTGVLATKAGCVAYGLSNPGGAIDGNWHQVWLQLIGAAFIIALNLVMTYVILKVISLVVPLRMDEETLLIGDDAVHGEEAYAFFGEGERQPVLGD

*Acidithiobacillus caldus* AmtB

>gi|254969310|gb|EET26822.1| Rh family protein/ammonium transporter [Acidithiobacillus caldus ATCC 51756]

MTHAAASLPGANVFFLLMGAILVLAMHAGFAFLELGTVRRRSQVNALVKILSDFGISTIVYFFIGFHIAYGISFFGNVASLTDHGHGFEMVRFFFLLTFAAAVPAIISGGIAERARFVPQLLATAFLVGLVYPLYEGIVWNGNFGIQAWFSQVFGAPFHDFAGSVVVHAMGGWMALVAVWLLGPRRGRYGKDGSIHPMPPSNIPFLALGSWILIIGWFGFNVMSAQRLEAVQGLVALNSLMALVGGMLTALALGRGDPGFAHNGALAGLVAICAGSDVVQPIGALVIGGIAGAIFVYLFTLVQHRLRLDDVLGVWPLHGVCGVWGGIAVGIFGLRVLGGAGGVSFGAQCAGTLLGVLIALLGSAIVYGAIRRFIGIRLDPEAEFMGPDLAIHHVGAYPEADIERA

*Acidithiobacillus ferrooxidans* Amt-1

>gi|501530998|ref|WP_012537472.1| MULTISPECIES: ammonium transporter [Bacteria]

MSVAWLNTGDNAWQLTAATVVGLQSVPGLVVLYAGIVKKKWAVNSAFMAFYAFAAVLIAWVLWAYNMGFGNQWFPFVGTPHPIISMQDELKQALIPASNTSAAFPMSTMVYFQFVFAAITLVIMAGAVLGRMSFRAWMIFVPLWLTFSYTVGAFSLWGGGFLSSLGVIDYSGGYVIHLSAGVAGFVAAAVIGPRLARDRENFQPNNVLLMLVGAGILWLGWNGFNGGDPYAASRDAGAAVLNTNIATAVSVIVWTIMDIFYFKKPSVIGAVQGMITGLVAITPAAGVVDGWGAIAIGISSGVIPWLSMNLLGKTALFRKVDDTLGVFHTHAVAGLLGGVMTGLLATKAGCAAFSLSTPGGAIEGNWHQVWLQLVGAAFIIVLNIVVTYILLKLISLVVPLRMSEEELLIGDDAVHGEEAYAFFGDGERRPVTGD

*Acidithiobacillus ferrooxidans* Amt-2

>gi|501530995|ref|WP_012537469.1| MULTISPECIES: ammonia channel protein [Bacteria]

MQKGAKHQFLPVASGIGAWIWLASPLAQADTTTPFNSGDTAWMLSSTALVLLMTVPGLALFYAGMVRKKNVLATAAQSFAITALVSVLWMFIGYSLTFTSGNAFMGGLSRLFLNGMGLDSANGLAPTIPESVYMTFQMTFAIITPALIVGAFAERMKFSALLWFTGLWSLLVYAPIAHMVWGPGGWLAADGVLDYAGGTVVHINAGIAGLVAALVMGKRIGYRHDAMHPNNLMYTLIGASLLWVGWFGFNAGSAVAASDRAGMAMSTTQIATAVATLSWMFAEWLARGKPTVLGMTSGAVAGLVAITPASGFVGPMGALWIGLAAGVICFWAAVYLKNRLGYDDSLDAFGVHAIGGIIGALLTGVFAVKAIGGTAGMLEGNTGQLLIQATGVGVTIVYDAVVSFIILKVIDWTLGLRVIGEQEREGLDITQHGEQVYE

*Acidithiobacillus ferrooxidans* AmtB

MIPTGVTPPGGNVFFLLMGAILVFAMHGGFAFLELGTVRRRSQVNALVKILSDFGISTIVYFFVGYHIAYGISFFADVKALTASNHGFQMVRFFFLLTFAAAVPAIISGGIAERARFYPQLLATAFLVGLVYPFYEGIVWNGHYGIQAWFTSVFGAPFHDFAGSVVVHAMGGWMALMAVWLLGSRKGRYGKDGAVHAMPPSNIPFLALGSWILIIGWFGFNVMSAQQIAAVQGLVALNSLMALVGGMLAALAVGKGDPGFVHNGALAGLVAICAGSDVVQPIGALAIGAIAGIIFVYLFTFVQHRLRLDDVLGVWPLHGVCGVWGGLAVGIFGHQFLGGAGGVSFWSQLLGTALGVLVALAGSGIVYGSIKYFIGIRLDPEAEYAGADLSLHHVSAYPEEDIERA

*Anabaena variabilis*

>gi|75702889|gb|ABA22565.1| ammonium transporter [Anabaena variabilis ATCC 29413]

MNRHIQPWQRLLVLAIGSMVFAVFAPTIVQAANTPTLESLSETTIKLQISIDTTWVLLSGFLVFFMQTGFAMLEAGLVRQRSVVNTLLENFIDAAVTVLAWWAVGFGIAFGTSAGGLFGIDTFFLNQLPGADGSYPLGAPGSTAAINTYTLFFFQFAFAATASTITTGSMAGRTDFIGDLIYSAIMGAISYPIIVHWAWNSNGWLGKLSYHDFAGGSIVHTVGGWTALVGAYLLGPRPDRPPWGKLPPAHNLALATLGTMILWFGWYGFNPGSTLGTANPGLIGLVTINTTLAAGAGALTALIFLYVRTGKWDLVYCLNGSLAGLVAITAPCAYVAPWASVLIGFTGGISVVLGVSLIESLHIDDPVGAFSVHGISGMMGTLSIGFLGQAELTLNQKAGLFLGGGFDLLGIQMLGIVAITVFTVAFAFLMYGGLKAVGYLRVNAEADSIGIDAYEHGASVWPDIYPVEEVSKLQDQTKISENKTLEEQ

*Anabaena* sp. PCC7120 1

>gi|764950517|ref|WP_044520794.1| ammonium transporter [Nostoc sp. PCC 7120]

MNRHIRPWQRLLVLAIGSMVFAVFAPTIVQAVDTPTLESLSETTIKLQISIDTTWVLLSGFLVFFMQTGFAMLEAGLVRQRSVVNTLLENFIDAAVTVLAWWAVGFGIAFGTSAGGLFGIDTFFLSQLPGADGSYPLGAPGSTAAINTYTLFFFQFAFAATASTITTGSMAGRTDFIGDLIYSAIMGAISYPIIVHWAWNSNGWLGKLSYHDFAGGSIVHTVGGWTALVGAYLLGPRPDRPPWGKLPPAHNLALATLGTMILWFGWYGFNPGSTLGTANPGLIGLVTINTTLAAGAGALAALIFLYVRTGKWDLVYCLNGSLAGLVAITAPCAYVAPWASVLIGLTGGIAVVLGVSLIESLHIDDPVGAFSVHGISGMMGTLSIGFLGQEELTLNQKAGLLLGGGFDLLGIQMLGIVAITVFTVAFAFLMYGGLKAMGHLRVNAEADRIGIDTYEHGASVWPDVYSVEELSKPQEHTKISENKTLEGE

*Anabaena* sp. PCC7120 2

>gi|499304391|ref|WP_010995166.1| ammonia channel protein [Nostoc sp. PCC 7120]

MFQKILIIGVSTLLLLTSPIMGNAWAQVPGTTPPADTGDTAFMLISAALVLLMTPGLAFFYGGFVRSRNILNTLMMSFVLMAIVGVTWVLWGYSLSFAPGLPFIGGLQWFGLNGVGLETTGYLQGSEPANVVSYAGTIPHQAYMIYQAMFAIITPALISGAIAERMSFRAYCLFVLLWSTFIYTPLAHMVWAKGGFLGLYGGLGALDFAGGTVVHISSGVSALVAAIVLGPRKTHPDRLSPPHNVTFILLGAGLLWFGWFGFNAGSALSVASSTSGSFITNPATTAFVATNTSAAAGALMWLILEGVLRGKPTAVGAATGAVAGLVGITPAAGFVTPLASILIGFITAFVCFYAVSFKHKLNVDDALDTYPVHGVGGTVGAILTAFFATAEVNSGGKNGVLKGNFGELGVELAAIVIAYVIAAVGTWIILKIIDATVGLRVKEEAEYQGLDISEHGEEAYNSEFSDRIVP

*Anabaena* sp. PCC7120 3

>gi|499304390|ref|WP_010995165.1| ammonium transporter [Nostoc sp. PCC 7120]

MYQQKSRTRNRRFSTRNYAKSRQSNSQIQIFNLVKKLSPSWQACIPLACLIVLGWSYVAVAQAPAAGPTTAELKVALDTLWVAIAAFLVFFMNAGFGMLETGFCRQKNAVNVLAKNLIVFALATVAFWAIGFGLMFGDGNDFIGFNGLFLSGVDNSPATGDAYKGVFSALSWAGVPLAAKFLFQLVFAGTAATIVSGAVAERIKFVDFLIFSLLLVGIAYPITGHWIWGAGWLAKAGFWDFAGSTVVHSVGGWAALMGAAFLGPRIGKYQDKQIVALPGHNMSIATLGCLILWLGWFGFNPGSVMAADPNAITHIALTTNMAGAVGGIAATATAWLYLGKPDLSMIINGILAGLVGITASCAYVSIPSSIIIGLIAGVIVVFSVTFFDKLGIDDPVGATSVHLVCGVWGTLAVGLWSVGPGVYSWYGEGLGPTKGLFAGGGLGQLITQFLGAAAVGGMTVLVSSIFWVVLKATLGIRVTREEELEGLDIGEHGMEAYSGFLKEASPGGFAEGKTSDGY

*Aquifex aeolicus*

>gi|15605698|ref|NP_213075.1| ammonium transporter [Aquifex aeolicus VF5]

MRALGFIGIILSIFSSFAYASEAKLDTGNTAWMLVASALVVFMTVPGLALFYGGLDKSKSILNTIAMSFSAFAVVTLTWIFVGYSVAYGDDIFGFIGNPFQYVLGKGISGINSDTGYPALLDLMFQLTFATITTALISGSFVGRMKFSAWILFAILWSVFVYPPVAHWVWGGGFLANDGALDFAGGTVVHINAGIAGLVGALILGRRKDTSLIPNNVPLVALGAGILWFGWFGFNAGSALGANESAAWAMINTTVATSTAALAWMFTEWLHVGKPTVVGISSGIVAGLVAITPAAGFVNLIGSIFIGAIASVCAYFMVALVKPKFGYDDALDVFGIHGVRGIVGAVLTGVFADPNVGGTPGLLYGNPKQVLIQIEGVIATILYSAILTAVILLVLKAVVGLRVSEEEELELDSSLHGEKAYNL

*Carboxydothermus hydrogenoformans*

>gi|77997093|gb|ABB15992.1| ammonium transporter [Carboxydothermus hydrogenoformans Z-2901]

MKKKFLSLLFGLILLPNLSLAGEPGYNPGDVTFVLLSAALVFLMTPGLALFYGGMVRRKNVLNTVAMSFIAIVVVSLQWILIGYTLSFGNDVKGIIGDFSYLGLKNVMGLTVSNLPGYVFVVFQLMFAIITAAIISGSVVERMSFAAWILFIALWTTLVYDPLAHMVWGGGMLFKLPTLDFAGGTVVHISSGISGLVAALILGKRKEGENAPLMPHNLPLTILGAGLLWFGWFGFNAGSALAANNLAALAFINTNTSAVMAALAWALAEWLHRRKVTVLGIATGAVAGLVAVTPAAGFVTPTSSVFIGLVGGVLVYIAVGILKAKFNYDDSLDAFGCHGVGGIWGAVATGIFATKAVNPAGADGVLYGNFGQLLNQLAGVGLAIALAVVGTVIILKLIGIFLPLRVKDEAESIGLDITVHGEDAYKDLIFTGKSVPFS

*Deinococcus radiodurans*

>gi|15805720|ref|NP_294416.1| ammonium transporter [Deinococcus radiodurans R1]

MKRAPYTAAATTAALLLLPLLSTAFAADAKPHLDTGDTAWMLMSAALVFLMTPGLAFFYGGLTRAQSVLNTMMMSMVSIGLVGVLWMLAGYSLAFGDGGAWLGDFAHLGLNGLAGQLTGTIPTYVFASFQAMFAIIAVALISGAVVERMRFGAFILFAALWTLIIYSPLAHWVWGANGWLFKDGALDFAGGTVIHISAGVSALVAAWLLGPRLGYPRNAHVPHNVPLVLLGAGLLWFGWMGFNAGSALGANQTAALAFITTLIAPAAAMLTWLGWESLSSKPTAVGAATGLVVGLVAITPACAFVSPWAAVLLGILGATASYWTVQAKHRLMADDALDVFACHGVAGIVGALLTGLLAWTTGSGKPIGAQFMTQLISVVASLLYAGVGSFILLKLVSLVIPLRVPAGQEIAGIDLSAHQEQGYSENELGIGAPVFLGGD

*Escherichia coli* AmtB

>gi|1786656|gb|AAC73554.1| ammonium transporter [Escherichia coli str. K-12 substr. MG1655]

MKIATIKTGLASLAMLPGLVMAAPAVADKADNAFMMICTALVLFMTIPGIALFYGGLIRGKNVLSMLTQVTVTFALVCILWVVYGYSLAFGEGNNFFGNINWLMLKNIELTAVMGSIYQYIHVAFQGSFACITVGLIVGALAERIRFSAVLIFVVVWLTLSYIPIAHMVWGGGLLASHGALDFAGGTVVHINAAIAGLVGAYLIGKRVGFGKEAFKPHNLPMVFTGTAILYIGWFGFNAGSAGTANEIAALAFVNTVVATAAAILGWIFGEWALRGKPSLLGACSGAIAGLVGVTPACGYIGVGGALIIGVVAGLAGLWGVTMLKRLLRVDDPCDVFGVHGVCGIVGCIMTGIFAASSLGGVGFAEGVTMGHQLLVQLESIAITIVWSGVVAFIGYKLADLTVGLRVPEEQEREGLDVNSHGENAYNA

>gi|37522630|ref|NP_926007.1| ammonium transporter [Gloeobacter violaceus PCC 7421]

MLMKCKWKWGLGTLAAALATAAPAFAQDAPKIDTGDTAWVLVSAALVLLMTPGLAFFYGGLVRGKNALNTLMMSFVALGVIALVWTLVGYSIAFGPGNGYFGSFAWLGLNGVGQDPNTAQAATVPHLAFMIFQMMFAVITPALISGAIVDRMKFKTYVVFIMIWSVVVYSPVAHWVWSLGNPDPTDAAKMLPGWLGAIGALDFAGGTVVHLTAGISALVAAIILGPRKGFPNQPMTPHNVPFVLLGAGLLWFGWFGFNAGSALAANGLASLAFVTTNVATAAALSTWLLLDTFIGGKPTAVGAATGAVVGLVAITPAAGFVSPLSSIAIGGIAAIISFAAIQLKKKLNYDDSLDVFACHGMGGLTGAILTGVFADKSLNSLGDNGLLLGNGAQLIEQLIGVGAVAVYAAVCTTVILLALKFTLGLRPSEEAEQEGLDTAEHGEEAYTGSIGGLGAMEEAMSSTSQGQGFTRPATQQQE

*Listeria innocua*

>gi|16414038|emb|CAC96782.1| lin1551 [Listeria innocua Clip11262]

MESVFMFFCTLLVWLMTPGIALFYGGMVRRKNVLSTAMYSFSSMAIISILWVIVGYSLAFAPGNGFIGSFDWTFLHNVGFAANDTYSDAIPHILFMMFQMTFAILTVAIISGAFAERMNFSAYLIFIILWSLLVYSPVAHWVWGDGGWLRELGALDFAGGNVVHISSGVTGLVLAIMIGRRKEADSASPHNLPLALIGGILVWFGWYGFNVGSALTIDNVAMTAFVNTNTAAAAGIIGWGLVEWLINKKPTMLGTISGAIAGLVSITPAAGFVTVPSSLIIGFLGGALCFWAVFWLKGKVKYDDALDAFGLHGIGGIWGGIATGLFATTKVNEAGADGLFYGNASLVVKQLIAIGSTVAYVAVVTALIVVVIKLFLPIRVNEEQEYKGLDLTLHGEKAYQE

*Listeria monocytogenes*

>gi|16410945|emb|CAC99594.1| lmo1516 [Listeria monocytogenes EGD-e]

MESVFMFFCTLLVWLMTPGIALFYGGMVRRKNVLSTAMYSFSSMAIISILWVIVGYSLAFAPGNGFIGSFDWTFLHNVGFAANATYSDAIPHVLFMMFQMTFAILTVAIISGAFAERMNFSAYLIFIILWSLLVYSPVAHWVWGDGGWLRNLGALDFAGGNVVHISSGVTGLVLAIMIGRRKEADSASPHNLPLALIGGILVWFGWYGFNVGSALTIDNVAMTAFVNTNTAAAAGIIGWGLVEWLTNKKPTMLGTISGAIAGLVSITPAAGFVTVPSSLIIGFFGGALCFWAVFWLKGKVKYDDALDAFGLHGIGGIWGGIATGLFATTKVNEAGADGLFYGNASLVVKQLIAIGSTVAYVAVVTALIVVIIKLFLPIRVNEEQEYKGLDLTLHGEKAYQE

*Methanosarcina acetivorans* 1

>gi|499333600|ref|WP_011024092.1| ammonium transporter [Methanosarcina acetivorans]

MLKNVQKLGKFLIIICMIFIALSSPAFAATAEENAVAIEGVQTALTFVWLLLASGLVFFMHAGFSLVETGLTRSKNTANILMKNFMTIVLGILIYWAVGWGVMYGADAAGLIGTDQFFLSGADNALWNGWFFQMVFAATGATIVSGAMAERTNFKAYLIYCIMMVAVIYPVYGHWVWSGADLALLTGADSPIVQAIGVPSHDFAGSGVVHSIGGYSALAGVLLVGARIGKFKDGKAVPIPGHNLTIAFLGTLILALGWIGFNGGSTLDGNDPYMNLVVVNTFLAAAAGAVVTMLITWMKTGKPDPSLTANGLLGGLVAITAPCGSVNNWAAIIIGAIAGIIIYAGVMFNENTLKVDDPVGAIAVHGYCGSWGLISVGLFSIGIGNGILADAAYAAEVPGLFYGGGISLLLIQLISVLCSIVWAFGISFIIFKILDAVVGLRVSEEEEIMGLDICEHGIRAYPEYLMREE

*Methanosarcina acetivorans* 2

>gi|499333322|ref|WP_011023814.1| ammonium transporter [Methanosarcina acetivorans]

MINTGSTGFMLLATSLVMLMTPGLAFFYGGLACKRNILGIMMQSFVSLGLTTILWFFVGYSLCFSGGEGAILGNLDKMFLNGIAPTDMFGDAGFPELVFVAYQMMFAIITPALITGAFANRITFKAYIIFLVVWQLFVYYPFVHMVWGGGLLAEMGVIDFAGGIVVHATAGFAALASVFYVGSRKYKDSKPNNIPLMAVGTALLWFGWYGFNAGSELNVDGITALAFLNSDIAASFAAITWMIIEWLRETKPKFVGLMTGAVAGLATITPAAGLVSMHVAALMGILGAIACYFAVHIKNRMQWDDALDVWGVHGIGGVTGTILLGIFASTAINPVGADGLFYGGTAFFIKELSVVLGASIYAFIFTYIMLMLINVITPVKVSDEEELAGLDISMHGECAYDAIH

*Methanosarcina acetivorans* 3

>gi|851004967|ref|WP_048065844.1| ammonia channel protein [Methanosarcina acetivorans]

MAIVAADTVWVLISSALVLLMLPGLALFYGGLVQRKNVLSSMMHSFVAMGVMALEWVIIGYSLAFGSGNGFIGSLEHVFLKGIDPYSVTGTIPTYAFIAFQGMFAIITPALISGAIVGRVKFKSYIAFIALWGLIVYAPVCHWVWGGGFLTGEALDFAGGTVVHITSGISSLAILTFLGKRRGYGIDSIKPHNVTLTLLGTGLLWFGWFGFNAGSSLAANEIAALAFITTLVAPAAAGFVWMTLEWIHLGRPTALGFATGVLAGLVAITPAAGFVTPMAAIPIGVVTSFICYQAVMLKGRLGYDDSLDAFGVHGVGGISGAILTGVFASIGSSGLLLGNSEQLMLQVEGVIATLVYASVCTLIIGFVLHKTIGLKVSESEENIGLDRTQHGEAAYNI

*Mycobacterium tuberculosis* Amt

>gi|1405956|emb|CAA98980.1| PROBABLE AMMONIUM-TRANSPORT INTEGRAL MEMBRANE PROTEIN AMT [Mycobacterium tuberculosis H37Rv]

MDQFPIMGVPDGGDTAWMLVSSALVLLMTPGLAFFYGGMVRSKSVLNMIMMSISAMGVVTVLWALYGYSIAFGDDVGNIAGNPSQYWGLKGLIGVNAVAADPSTQTAAVNIPLAGTLPATVFVAFQLMFAIITVALISGAVADRLKFGAWLLFAGLWATFVYFPVAHWVFAFDGFAAEHGGWIANKLHAIDFAGGTAVHINAGVAALMLAIVLGKRRGWPATLFRPHNLPFVMLGAALLWFGWYGFNAGSATTANGVAGATFVTTTIATAAAMLGWLLTERVRDGKATTLGAASGIVAGLVAITPSCSSVNVLGALAVGVSAGVLCALAVGLKFKLGFDDSLDVVGVHLVGGLVGTLLVGLLAAPEAPAINGVAGVSKGLFYGGGFAQLERQALGACSVLVYSGIITLILALILKFTIGLRLDAEQESTGIDEAEHAESGYDFAVASGSVLPPRVTVEDSRNGIQERIGQKVEAEPK

*Nautilia profundicola* Amt

>gi|506382310|ref|WP_015902029.1| ammonium transporter [Nautilia profundicola]

MDFAWVIDTFFALFAFTLIILMVPGFAMLEAGLVRTKNVTAVLTVNTMIYIVASLVFFLWGYTLAFGGWDGSSMSMYAAFLFQMAFVGKTVNIMSGGVSERTRIVPLAIFTLFMAGLIYPLVVNWTWGANMLSGSILDISSMHDLAGSTVIHSTGAWALLAAILIIGPRKGRYTKDGKVRVIPASNIPLVVLGALLLWIGWFGFNGGSVGSISSKENADTVALVVMNTNNAGLIGGLVVMILGYLKYRKFDITMILNGALGGLVAITAGADVVSPYAALIIGAIGGALVFYAVPFFDKLKIDDPVGALSVHLVNGIWGTIATGIWGSGVSLAAQIKGVVVVAVFAFVASYIIIFVINKITKFRTDEDNEVEGLDVSECGIESYPEFKRAI

*Neisseria meningitidis* MC58 AmtB

>gi|15676519|ref|NP_273659.1| ammonium transporter AmtB [Neisseria meningitidis MC58]

MKKHIWAASLLPASLSAEPLNWWKPYSAVNSGDTAWVMTAAALVLLMTLPGLALFYGGMVRKKNLLSTMMHSFSIATLVGILWVAVGYSLAFTPGNAFIGGLGRVFLSGMQIDATAQMLTVSPNAPTVPEPVFMFFQMTFAIISTAIITGAFAERMKYSAMMLFSGIWFLLVYVPGAHWVWGGGFMSKGGVLDYAGGTVVHINAGIAGLVAALVLGRRIGYGREAMPPHNMAMTLIGAAMLWFGWFGFNAGSALAADAAAGMAMAVTQVSAVFGAAGWLACEKIAGHKPSALGLASGAVSGLVGITPAAGFTGPSGAAAIGILTAAACFVSVTVVKHKLRYDDSLDAFGIHGFGGLVGGILTGIFFDNRIFGGDAAVWQQLWIQVKDGVVMAAYSGLMSWAILKVVGKICGGLRVGKDVEREGLDLNIHGERVE

*Neisseria meningitidis* AmtB

>gi|488143071|ref|WP_002214279.1| ammonia channel protein [Neisseria meningitidis]

MKKHIWAASLLPASLSAEPLNWWKPYSAVNSGDTAWVMTAAALVLLMTLPGLALFYGGMVRKKNLLSTMMHSFSIATLVGILWVAVGYSLAFTPGNAFIGGLGRVFLSGMQIDAAAQMLTVSPNAPTVPEPVFMFFQMTFAIISTAIITGAFAERMKYSAMMLFSGIWFLLVYVPGAHWVWGGGFMSKGGVLDYAGGTVVHINAGIAGLVAALVLGRRIGYGREAMPPHNMAMTLIGAAMLWFGWFGFNAGSALAADAAAGMAMAVTQVSAVFGAAGWLACEKIAGHKPSALGLASGAVSGLVGITPAAGFTGPSGAAAIGILTAAACFVSVTVVKHKLRYDDSLDAFGIHGFGGLVGGILTGIFFDNRIFGGDAAVWQQLWIQVKDGFVMAAYSGLMSWAILKVVGKICGGLRVGKDVEREGLDLNIHGERVE

*Nostoc punctiforme* 1

>gi|501379412|ref|WP_012410978.1| ammonium transporter [Nostoc punctiforme]

MNKHIRPWQRLLALAIGSMVFAVFAPTIVQATDPPTVQSLSETTTKLQISIDTIWVLITGFLVFFMQTGFAMLEAGLNRQRGVVNALLENFINAAVTILVWWGIGFGIAFGTSAGGLVGIDTFFLSQLPGADGSYILAAPGSTAAINTYTLFFFQFAFAATATTIATGSMAGRTDFIGDLIYSAIMGAIAYPIVVHWVWNSDGWLSKLSYHDFAGSSVVHTVGGWTALVGAYLLGSRPGRPAWGTLPPAHNLGLATLGTMILWFGWYGFNPGSTLSTGNTGLIGLVTVNTTLSAGAGALSAIIFQYTRKGKWDLVYSLNGSLAGLVAITAPCAYVAPWASVLIGLTSGILVTVGMDLIESVHIDDPVGAFAVHGINGMMGTLSVGFLGQAELTLNQKAGLFLGGGFELLGIQLLGVVAIAVFTVAFSFLMFGGLKAVGHLRVNSEADRIGIDAYEHGASVWSDVYAIEKKEINCAETTEIEALDSE

*Nostoc punctiforme* 2

>gi|753809830|ref|WP_041565463.1| ammonia channel protein [Nostoc punctiforme]

MLKKVVMIGAMTLLFLAGPLMGNAFAQTPVAVPAADTGDTAFMLISAALVLLMTPGLAFFYGGFVRSRNILNTLMMSFVLMAIVGVTWILWGYSLSFAPGLPFIGGLQWFGLNGVGLETQGYLPHLPYEDALKAADPKYADVVSYAGTIPHQAFTIYQAMFAIITPALISGALVERMSFRAYSLFVLLWSTFVYAPLAHMVWAKGGFLGLAGGLGALDFAGGTVVHISSGVSALVAAIVLGPRKTHPDRLSPPHNVPFILLGAGLLWFGWFGFNAGSALSVASGTSGNLTTNLATTAFVATNTAAAAAALMWLILEAVLRGKPTAVGAATGAVAGLVGITPAAGFVTPLSAILIGFITAFVCFYAVSFKHKLQIDDALDTYPVHGVGGTVGAILTAIFATTQVNGGGKDGVLRGNFGELGVELIAIAVAYAIAGVGTWIILKVIDATVGLRVKEEAELQGLDINEHGEEGYNSEFGDRPT

*Rhodopirellula baltica* AmtB

>gi|32442976|emb|CAD71497.1| ammonium transporter [Rhodopirellula baltica SH 1]

MAEITLHPPSSPSTFHWSLPGMLSRVLVVVCLLTCILGVPLVGLPVASVGAQDEVANTSAAETAPAEPSASAEEIAPAEENEAAEEPAAPTLESLQAGVDEALLAGHNGWMLVCCALVLFMTAPGLAMFYGGLVRRKNVLSVMMQCIFLMGMMTVLWALYGYSFAFGGSGGWFGNTDYLFMEGVQRYWDDDLGKAITPMEGAMTRMTHMLFQGMFFIITPALICGAFAERMKFSAMVVFSILWGTFIYCPLTHWVWDEGPLSYFAGDKALAGGALDFAGGTVVHISSGISALVAALLIGPRMGYPREPLQPHNLTYTALGAAMLWVGWFGFNAGSELASDDLTASAFAVTHFSAAAGAVAWVTLEWLVLGKPTVLGASSGAVAGLVCITPAAGFVQPMPALIMGAMAGGVCYWACSTLKHKLRYDDALDAFGVHGVGGTLGAILTGVFATRAAWDVSDGVPIGLIESGGDFTLVIGQVVAVLVTYVFAGVGSLILLKLIDICIGLRVPAESEQRGLDISDHGEEGYQFA

*Rhodopirellula baltica* 2

>gi|32447224|emb|CAD77050.1| putative ammonium transporter [Rhodopirellula baltica SH 1]

MDTVRRATNMVLNVDSEASLSLSQISGVEGGSFQDDWLILCAVIVLLMQAGFMCLESGLVREKNSINVAVKNLADLMISVASFWTIGFSVMFGATIFGLFGVGQLMPDFGVSDSLAAFFVFQAVFCGTATTIVSGAVAERMRFHGYLVSCVCVSALIYPVVGHWVWASGANGMSIGWLEGLGFRDFAGSTVVHSVGGWAALAGCIVLGPRLGRFRKDGRVRDIQPSNLTLAYLGAFILFFGWFGFNCGSTFKATGEIALIAINTLLGGCFGGLSSILVSSLRTGIPDPRSIANGLLGGLVGITASGSMIDPRAAALVGAIAGVVAVYSDEWLVRLRIDDAVGAISVHGFCGAWGTIAFALFIRTDELAPGMSRWEFLAVQVFGVVVTFGFVFGGVFVLLRGLGRFIQLRVDPETERLGLNVAEHNASSRLLDLAVSMDAAARMRKLEPSCKVRSEYGTAIGDLIDSYNQMIDHICEDQTALAEATKRAEALTAILDDSPNEIAIVHADTGQVLSLNRGARVNLQRDEDEFSELTFDCLFNTEFGELQKFRLSAFDDEAGFTQQLATAVRKDGTTYPASISVQHAEFLGYQVLVVLTVDMTSQVELKERLHAAEKMEAVGQLAAGIAHEINTPLQCMSGNVEFLQSVQQNLQDTLRLVAEGVSDEQALHEITEKLSSDRWSRLLGESDAAIEDTQKSIGRVVQIIGAMRVLSHPGPAGKTNTSINGILRDSAAISRGRWKYAAQMDFDLCDSEPTVACRANELSQVFCNLFVNAADSIEEKNEKVPEAGLGNIFVSSSIEQDGVLIKVRDSGVGMSESVRRRCFEQFYTTKPVGKGTGQGLSIAYQIIVGRHGGNIEITSEEGVGTEFQIWIPMHASDDACDGATKTNAGQTNDATSLAAV

*Salmonella enterica* AmtB

>gi|16501735|emb|CAD08923.1| probable ammonium transporter [Salmonella enterica subsp. enterica serovar Typhi str. CT18]

MKIAMIKTTLASLALLPGLTIAAPAVADKADSAFMMICTALVLFMTIPGIALFYGGLIRAKNVLSMLTQVTVTFALVCILWVVYGYSLAFGEGNHFFGNADGAMLKNIALTAVTGTIYQYIHVAFQGSFACITVGLIVGALAERIRFSAVLIFVVVWFTLSYIPIAHMVWGGGLLAAHGALDFAGGTVVHINAAIAGLVGAYLIGKRVGFGKEAFKPHNLPMVFTGTAILYIGWFGFNAGSAGSANEIAALAFVNTVVATAAAILGWIIGEWTLRGKPSLLGACSGAIAGLVGVTPACGYVGVGGALVIGVIAGLAGLWGVTMLKRLLRVDDPCDVFGVHGVCGIVGCILTGVFAASSLGGVGFVDGVTMGHQVMVQLESIAITVVWSGVVAFVGYKLADILVGLRVPEEQEREGLDVNSHGENAYNA

*Staphylococcus aureus* subsp. *aureus* Mu50 NrgA

>gi|446975014|ref|WP_001052270.1| ammonia permease [Staphylococcus aureus]

MNLNDTIFMFLCTLLVWLMTPGLSLFYGGLVQSKNALNTVMQSMAAIVLVTFVWITVGFTISFGNGNLWFGNWEYTFLNHVGFATQEDISPHIPFALFMLFQMMFCTIAISILSGSIAEKMKFIPYLLFVVIWTALVYSPVAHWVWGGGWINKLGVLDFAGGTVVHITSGVSGLVLAIMIGKGNKHSESTPHNLIITLIGGIFVWIGWYGFNVGSAFTFDNIAMLAFTNTVISASAGAIGWLILEYIFKKTTSLLGLLLGALAGLVVITPAAGYVTYLSATIMALIGGICCYIVINYIKVKLKYHDALDAFGIHGVGGIIGAVLTAVFQSKKANPDIENGFIYTGDIHIILVQILCVTAVVIFSIVMTFIIAKVIKLITPLSVTEQETNIGLDKIVHGEHAYFEGELNRFNKHIRY

*Staphylococcus aureus* subsp. *aureus* MW2 NrgA

>gi|21205138|dbj|BAB95832.1| probable ammonium transporter [Staphylococcus aureus subsp. aureus MW2]

MNLNDTIFMFLCTLLVWLMTPGLSLFYGGLVQSKNALNTVMQSMAAIVLVTFVWITVGFTISFGNGNLWFGNWEYTFLNHVGFATQEDISPHIPFALFMLFQMMFCTIAISILSGSIAEKMKFIPYLLFVVIWTALVYSPVAHWVWGGGWINKLGVLDFAGGTVVHITSGVSGLVLAIMIGKGNKHSESTPHNLIITLIGGIFVWIGWYGFNVGSAFTFDKIAMLAFTNTVISASAGSIGWLILEYIFKKTTSLLGLLLGALAGLVVITPAAGYVTYLSATIMALIGGICCYIVINYIKVKLKYHDALDAFGIHGVGGIIGAALTAVFQSKKANPDIENGFIYTGDIHIILVQILCVTAVVIFSIVMTFIIAKVIKLITPLSVTEQETNIGLDKIVHGEHAYFEGELNRFNKHIRY

*Staphylococcus epidermidis* AmtB

>gi|27468562|ref|NP_765199.1| probabale ammonium transporter [Staphylococcus epidermidis ATCC 12228]

MNMNDTIFLFLCTLLVWLMTPGLSLFYGGLVQSKNALNTVMQSMVAIVIVTFIWIMIGFSLSFDGGNQWIGGLKFLGLHHVGFETSKTLSPHIPLSLFMLFQMMFCTIAVSILSGSIAEKMRFIPYLIFVSLWVLLIYSPVAHWVWGGGWISKIGAIDYAGGTVVHITSGVSGLVLGIMIGIGKKKEKHTPHNLLITLIGGILVWLGWYGFNVGSAFTFDHIAMISFVNTVIGASAGAFGWLIFEYILKKTTSLLGLLSGALSGLVAITPAAGYVSYMSAMIIAIMGGIGCYIVINLIKVKLQYNDALDAFGIHGVGGILGAVFTGVFQSHQINSAVENGFIYTGDFKIVVIQLGAAIATVVFSAIVTFLIARFIKIFTPLATTQEEDKTGLDVIVHGEKAYFYGELNKFNRHIKF

*Streptococcus mutans*

>gi|24380036|ref|NP_721991.1| ammonium transporter [Streptococcus mutans UA159]

MDSGSIAFIIICSALVFLMTPGLAFFYGGLGRRKNVINTMMMAVVPLALASLLWVVVGYSLSFSGVGKVFGDFSHVFLNGVKEGASSRGLTIPDTLFSGFQMMFSIITVAILTGAVAGRMRFTPLVIFIIFWLLLVYYPFAHMVWDDGLLAKWGTLDFAGGDVVHITSGVSGLVLALVVGKRRDYDRLEYRPHNIPFVLLGAGLLWFGWFGFNAGSALAANGLAVHALLTTHISAAAAMFSWLLLEKYLNGKPSLVGGSTGLVAGLVAITPGAGFVPLWSSLLIGLMVSPLCYFAIAVLKSKFGYDDALDAFGCHGIGGIFGGIVTGLFTTPHLALDKSNIGLIYGNARLFLVILAAILFTIIWSALISFLIIKVISLFMPIRVSDREEATGLDDKEHGETAYPTFMGLDS

*Streptomyces avermitilis* AmtB1

>gi|499292827|ref|WP_010984085.1| ammonia channel protein [Streptomyces avermitilis]

MAPAITLAAEAPKLSAANTGFMLICSALVLIMTPGLAFFYGGMVRVKSTLNMLMMSFISMGIVTILWVLYGFSLAFGTDKGSLIGWTSDFVGFTGIGKVELWPGYTIPVYVFATFQLMFAIITPALISGALADRVKFTAWSLFVALWATVVYFPVAHWVWGTGGWAFDLGVIDFAGGTAVHINAGAAALGVILVIGKRVGFKKDPMRPHSLPLVMLGCGLLWFGWFGFNAGSWLGNDDGVGALMFINTQIATAAAMLAWLAYEKIRHGAFTTLGAASGAVAGLVAITPSGGAVSPLGAIAVGAIAGVLCAMAVGLKYKFGYDDSLDVVGVHLVGGVAGSLLIGFFASGGGQSDATGVFYGDHSFDQLWKQCAGVFAVLAYSLVVSAILAFLIDKTIGMRVSEDEEIAGIDQAEHAETAYDFSGAGGGAARILPAPLADTATKKVDA

*Streptomyces avermitilis* AmtB2

>gi|499295738|ref|WP_010986996.1| ammonia channel protein [Streptomyces avermitilis]

MTLAAARIDTGDTAWLLAATALVLLMTPGLALFYGGMVRTKSVLNMLMMSFVSIALVTVVWLAAGYSLAFGDDLAGGLIGGLDHAGLADLGPADVHGTVPTLLFATFQLTFAIITAALISGAIADRAKFAAWLVFVPLWALLVYVPVAHWVWGPGGWILDRLGALDFAGGLPVEITSGASGLALCLVLGPRLGFKKDAMRPHNLPMVMLGAGLLWFGWFGFNAGSALGANGLAAAAFLNTLAAGCTGLLGWLFVEQKRDGHPTTLGAASGAVAGLVAITPSCGAVSLLGALVVGLAAGVVCSYAVGWKFKLNYDDSLDVVGVHLVGGIIGTLLIGVFAAKEMTGGTEGLLYGGGLGQLGKQLVAVVAVGAYAFAVTYGLGKLIDKVMGLRAAEDDEHQGLDLTVHAETAYDHGILGHGAPSSASVVPTAQKVKSQA

*Streptomyces coelicolor*

>gi|4007742|emb|CAA22426.1| ammonium transporter [Streptomyces coelicolor A3(2)]

MAPAITLAAETELSAANTGFMLICSALVMLMTPALAFFYGGMVRVKSTLNMLMMSFISLGIVTVLWVLYGFSMAFGTDSGSVVGWNSDWVGLSDIGLTELWDGYTIPVFVFLVFQLMFAVLTPALISGALADRVKFTAWALFIALWATIVYFPVAHWVWGAGGWAFELGVIDFAGGTAVHINAGAAALGVILVIGKRVGFKKDPMRPHSLPLVMLGAGLLWFGWFGFNAGSWLGNDDGVGALMFVNTQVATGAAVLGWLAYEKIRHGAFTTLGAASGAVSGLVAITPAGGAVSPLGAIAVGLVAGVVCAMAVGLKYRFGYDDSLDVVGVHLVGGILGSLLIGFFASGKGQSDVEGLFYGGGLDQFWKQCAGVFGVLAYSLVVSAVLAFLLDKTIGMRVSEDVEVAGIDQAEHAETAYDFSGAGGGAARTTATPAASTAGSAPSKKVDA

*Synechocystis* sp. PCC 6803 Amt1

>gi|499176500|ref|WP_010874087.1| ammonium transporter [Synechocystis sp. PCC 6803]

MTSIDTLWLLLCAGLVFFMQAGFMCLESGLTRSKNSINVAIKNFADFGISVALFWSFGFSIMFGLSQGGWWGTGYSFVDVGGEPTLAVFFLFQAMFCGTATTIISGAAAERLKFSAYLLVAGLASGLIYPLFGDWAWNGLATVAGIETTGGWLENLGFRDFAGSTVVHSVGAWIGLATILVVGPRQGRFPKTGKTLKIQGSNMPFSVLGTLILWFGWLGFNGGSTFGLTPEVPGIMVNTVLAGVGGMLMAGLISLLQDKMIQVEPLMNGSLAGLVAITASANVVMTPIAMVIGATGSAIAYLVGKKMLHWGVDDAVDAVAVHGGAGVWGTLCVGLFGQLPLVDTGLNRWQQCGVQLLGIGVCTLWAFGLAWVFLTLLNRVFALRISPEDEEIGLNVSEHQATTETYELFQVMDRQAKTHDLSLRVPVNPFTEVGHIAGRYNQVMDAFEARHHRSVEDLAQIYYVTAAIAAAIENNSFKADQLGLEEVTNRADELGALARTIQQMAEMLQQRDQELIAVKQQLVLQQQKQHHGNGESVDLSP

*Synechocystis* sp. PCC 6803 Amt2

>gi|499173994|ref|WP_010871581.1| ammonium transporter [Synechocystis sp. PCC 6803]

MKPKNFPLARYVLGAMLAFLFVGVAQAQTETTSIAEVTYAINNLFLLAAAVLVLFMQAGFAMLEAGLSSHKNTVNVLFKNTFDVCVGVLLYFLFGYSLMYGENPVLGGFFGWGGFGITNNLDNVEGLSPQVDWLFQAAFAATAATIVSGAVMGRMYFKAYLIYSAVITGLVYPISGHWKWGGGWLDKLGFHDFAGSLLVHSVGGFAALAAVVVMGPRIGRFEGNKINSLGYQGITSSSLGVFILWVGWYGFNPGSQLAFVGALNTNTTMLIAVNTTLSAAAGGLAALAFDWITENKRKPNLLVTLNGILGGLVGITAGCDTVSNWSAIAIGVVAGILSVLGTKLLDRLRIDDGVGAWPVHGLCGIWGGIAVGIFSTNVEHKLSAQIVGSLVIPFWAFITMFFLFYVMDLWGILRVKPSQEKVGLDIVEHGQTEKGVEIAFED

*Synechocystis* sp. PCC 6803 Amt3

>gi|752786202|ref|WP_041426058.1| ammonium transporter [Synechocystis sp. PCC 6803]

MGERSPVNRTHSSRTEAEKSSLFRFVRRKINSPWLACVPLTALIVAIWNAAAIAQDTEIVNITVETVNENVATLQGTLNAIWILIAAILVIFMNAGFGMLETGLCRQKNAVNILTKNLIVFALATIAYWAIGFSLMFGSSGNPFVGFGGFFLSGDHTNYGLSPFPEGLPVAVFFLFQVAFSATAATIVSGAVAERIKFNEFLIFSVLLVGIAYPITGHWVWDAGGWLYTMGFMDFAGSTVVHSVGGWAALAGAFLLGPRLGKFVDGRPGAIPGHNMGFAMLGCLILWIGWFGFNPGSQLAADQACAYIAVTTNLAASAGGLTATFTSWLKDGKPDLTMVINGVLAGLVGITAGCAGVSYWGSVIIGGIAGILVVYSVAFFDKIKIDDPVGAISVHLVNGVWGTLAVGFFNMEKGLFYGGGINQLIIQIVGILAIGAFTAIFSFVVWAILKQTMGIRVSGEEEMIGLDIGEHGMEAYTGFVKETDSFGSAVSGATVPE

*Vibrio parahaemolyticus* 1

>gi|28899266|ref|NP_798871.1| ammonium transporter [Vibrio parahaemolyticus RIMD 2210633]

MELATTVTELRYALDTFFFLISGALVMWMAAGFAMLEAGLVRSKNTTEILTKNFCLYAIACTTYLVIGYNIMYVDNGEGGWLPSFGGLIGTQGEGADHSLESDFFFQVVFVATAMSVVSGAVAERMKLWSFLLFSAVLTAFIYPMEGYWTWGGGFLSEAGFSDFAGSGIVHMAGASAALAGVLLLGARKGKYGKNGEIYPIPGSNMPLATLGTFILWFGWFGFNGGSQLMVSDFENATAVGQIFLNTNAAAAAGSIAALFVCKTTWGKADLTMILNGALAGLVAITADPLSPSPLYAVAIGAVSGALVVFSIIGLDKLKIDDPVGAISVHGVCGFFGLMVVPLSNGEATFGAQLLGAAVIFAWVFGASLVVWAILKATVGIRVTEEEEMEGMDMHDCGVGAYPEFVTVK

*Vibrio parahaemolyticus* 2

>gi|28898297|ref|NP_797902.1| ammonium transporter Amt [Vibrio parahaemolyticus RIMD 2210633]

MVMTLTLMHRMSALQITEINIMSVTADQVHGVVQALTQSSDTLFLLLGAIMVFLMHAGFAFLEVGTVRQKNQVNALVKIIADFGISAIAYFFIGYWVAYGGTFFSDAQTLSQDNGYELVKFFFLLTFAAAIPAIVSGGIAERARFYPILIATFFTVGVVYPLFEGMIWNGNFGVQAWFESTFGYGFHDFAGSVVVHGVGGWIALVAVTFLGMRRGRIRAGKHTNFAPSNIPFLALGAWILCVGWFGFNVMSAQTLSGISGLVAMNSLMAMVGGIVASLLAGKNDPGFIHNGPLAGLVAVCAGSDLMHPLGALVTGAVAGVLFVYLFTYLQNRTKIDDVLGVWPLHGVCGAWGGIAAGVFGQSGFGGMGGVSFAVQVLGTFFGISIAVLGALVVYGAINALTGLRLSEEDEFNGADLAIHKISSTNED

*Vibrio vulnificus* CMCP6 1

>gi|27365021|ref|NP_760549.1| ammonium transporter [Vibrio vulnificus CMCP6]

MELTTTVSELRYALDTFFFLISGALVMWMAAGFAMLEAGLVRSKNTTEILTKNFCLYAIACTTYLVVGYNIMYVDNSEGGWLPSIGALIGSQAEGADHSLESDFFFQVVFVATAMSVVSGAVAERMKLWSFLLFSAILTAFIYPVEGYWTWGGGFLSAAGFSDFAGSGIVHMAGAAAALAGVLLLGARKGKYGKNGEVYPIPGSNMPLATLGTFILWFGWFGFNGGSQLMVSDFENATAVGQIFLNTNAAAAAGAITALLVCKTTWGKADLTMILNGALAGLVAITADPLSPSPLYAVAIGAVAGALVVFSIVGLDKLKIDDPVGAISVHGVCGFFGLMVVPISNGDASFLSQLFGAAVIFAWVFGASLAVWAVLKATIGIRVSEDEEMEGMDMHDCGVGAYPEFVTVK

*Vibrio vulnificus* CMCP6 2

>gi|27365893|ref|NP_761421.1| ammonium transporter [Vibrio vulnificus CMCP6]

MSDTASQVHGAVQTLTQSSDTLFLLLGAIMVFLMHAGFAFLEVGTVRHKNQVNALVKILADFGISTLAYFFIGYWVAYGAHFFADAETLAQGNGYELVKFFFLLTFAAAIPAIVSGGIAERARFYPVLIATFFTVGIVYPLFEGIIWNGNFGIQAWFEATFGVGFHDFAGSVVVHGVGGWIALVAVIFLGMRRGRVRAGKHTNFAPSNIPFLALGAWILCVGWFGFNVMSAQTLNGISGLVAMNSLMAMAGGIVAALIAGKNDPGFIHNGPLAGLVAVCAGSDLMHPLGALVTGSVAGVLFVYLFTYLQNKTKIDDVLGVWPLHGVCGAWGGIAAGIFGQSSLGGLGGVSFTVQLLGTLLGISVALIGAGIVYGAINRLSGLRLSQEDEFNGADLAIHKISSVNAE

*Vibrio vulnificus* YJ016 1

>gi|762206525|ref|WP_043877311.1| ammonium transporter [Vibrio vulnificus]

MELTTTVSELRYALDTFFFLISGALVMWMAAGFAMLEAGLVRSKNTTEILTKNFCLYAIACTTYLVVGYNIMYVDNSEGGWLPSIGALIGSQAEGADHSLESDFFFQVVFVATAMSVVSGAVAERMKLWSFLLFSAILTAFIYPVEGYWTWGGGFLSAAGFSDFAGSGIVHMAGASAALAGVLLLGARKGKYGKNGEVYPIPGSNMPLATLGTFILWFGWFGFNGGSQLMVSDFENATAVGQIFLNTNAAAAAGAITALFVCKTTWGKADLTMILNGALAGLVAITADPLSPSPLYSVAIGAVAGALVVFSIVGLDKLKIDDPVGAISVHGVCGFFGLMVVPISNGDASFLSQLFGAVVIFAWVFGASLAVWAVLKATIGIRVSEEEEMEGMDMHDCGVGAYPEFVTVK

*Vibrio vulnificus* YJ016 2

>gi|499463140|ref|WP_011150285.1| ammonium transporter [Vibrio vulnificus]

MSVTASQVHGAVQTLTQSSDTLFLLLGAIMVFLMHAGFAFLEVGTVRHKNQVNALVKILADFGISTLAYFFIGYWVAYGAHFFADAETLAQGNGYELVKFFFLLTFAAAIPAIVSGGIAERARFYPVLIATFFTVGIVYPLFEGIIWNGNFGVQAWFEATFGVGFHDFAGSVVVHGVGGWIALVAVIFLGMRRGRVRAGKHTNFAPSNIPFLALGAWILCVGWFGFNVMSAQTLNGISGLVAMNSLMAMAGGIVAALIAGKNDPGFIHNGPLAGLVAVCAGSDLMHPLGALVTGSVAGVLFVYLFTYLQNKTKIDDVLGVWPLHGVCGAWGGIAAGIFGQSSLGGLGGVSFTVQLLGTLLGISVALIGAGIVYGAINRFSGLRLSQEDEFNGADLAIHKISSVNAE

**B. Rh Sequences**

*Anopheles gambiae* Rh50

>gi|31323871|gb|AAP47144.1| Rh-like glycoprotein [Anopheles gambiae]

MHTPGSSTAGYALLLIVQVVFIIVFGFCTDYAKELLPVKNETARVHSPAESEGGNLRKYPHFQDIHVMIFAGFAFLMTFLKRYGFSASGLNLLVAALVVQWAIIMRGCYEMEDGIIPISLQNLIGADIAAAAVLISMGALLGRTTPIQLLIMGILEIAIFAGNEYLQLELVKAADVGGSITVHAFGAYFGLAVSFMLRPKKEQAKAGPLEGSSYSSDISAMIGTIFLWIFWPSFNSALVDGADQERAIINTYLSLAGATVTTFVLSALVSHEHKLDMVHVQNSTLAGGVAVGSICNLLIHPFGALIVGVMAGVISVLGYRFLTPAILSNLRIADTCGVHNLHGMPAVLSAIFSAIYASFASVETYGTSLATIFPAMQNPNATNATSEMEPLEYVIGGYGRSGAKQGAFQLMAIGLTMVIAIVGGLITGLILKSPSVRQLEEHELHKDDAFWETPSEESTNTTITTNESSN

*Asterochloris* sp. 4

*>Asterochloris* sp. (JGI 4349)

MFSASLTTIGSVCLVLLACFVDYAKDQSDAKVNQHYSWFLHVSASEGSVSIMIFVGFGFLMTFLRHYSYSAVGFNFFASCLMLGHAHVHTIELDLPLLIDAAFCAGAAMVTFGAVLGKVSPTQLTWLLTLEVCSILACLLMGSEEVPLYAANTYIASRLGALDIGGSMTIHAFGAFYGLAASLVLSKPGSGSSHAKNGATYTSDITAMIGTIFLWIFWPSFNGALASDPSLPASSAQFHCVMNTVLSLIGACLSAFALSAAYHGKLDMVHIQNSTLAGGVAIGSAANMDMAPAAALGVGMVAGTVSVLGFRWGDDLCNPFELIPACLPCRHISPYLETKVGLRDTCGVHNLHGLPGILGGVVAALILVVAPHRNALLLSHGRWGTAGFQLAALGASLLLATVGGVCAGRLVAAGAGRMEPEDAFEDARWWDEIEAESETALEEDRATGPRV

*Asterochloris* sp. 5

*>Asterochloris* sp. (JGI 4350)

MFSASLTTIGSVCLVLLACFVDYAKDQSDAKVNQHYSWFLHVSIMIFVGFGFLMTFLRHYSYSAVGFNFFASCLMLGHAHVHTIELDLPLLIDAAFCAGAAMVTFGAVLGKVSPTQLTWLLTLEVPLYAANTYIASRLGALDIGGSMTIHAFGAFYGLAASLVLSKPGSGSSHAKNGATYTSDITAMIGTIFLWIFWPSFNGALASDPSLPASSAQFHCVMNTVLSLIGACLSAFALSAAYHGKLDMVHIQNSTLAGGVAIGSAANMDMAPAAALGVGMVAGTVSVLGFRHISPYLETKVGLRDTCGVHNLHGLPGILGGVVAALILVVAPHRNALLLSHGRWGTAGFQLAALGASLLLATVGGVCAGRLVAAGAGRMEPEDAFEDARWWDEIEAESETALEEDRATGPRV

*Branchiostoma floridae* 4

>gi|260795927|ref|XP_002592956.1| hypothetical protein BRAFLDRAFT_202079, partial [Branchiostoma floridae]

YTSLSSVFQDVHVMMFIGFGFLMTFLKKYGFGSVGFNFLVAAFVLQWATLMSGFLHLHHGKIVVDITTLLTSDFAAAAVLISMGAVLGKVSHIQLIIMAFVEIIFFSINEWVGLTFFKAVDVGGSMFVHAFGAYFGLAVARVLYREDMVGHSKEGSNYHSDIFAMIGTVFLWLFWPSFNAALAPGDDQHRAVINTYFSLAACAVVTFAISSAVEKDGKVNMVHVQNATLAGGVAIGTSADMMVHPWGALLIGSIAAVLSVVGYSYITPFMADKLKIHDTCGVNNLHGMPGVLAGIIGAIVAAAGNGRSAGLQAGFQMAALIVSVLVALVTGALTGLLLRLPIWDQPEKDSMYEDEDYWEVSTESTSVLVCLF

*Branchiostoma floridae* RhR1

>gi|299823637|dbj|BAJ10270.1| Rh-related protein [Branchiostoma floridae]

MTSNYAPLLGRDSRPDRRGRLTAFLLAFQALFLGVFAGFVRYGPPAGPGIAGAGPTIDHYYPMFQDVHVMIFVGFGFLMTFLKMYGYGSVGFNMLVAAFVLQWAILMRGFLYMEDNTINLDISSMLSADFAAAAVLISYGAVLGKTTPVQLVVMAFLEIAFLTVNEWIGTVQFKAQDIGGSMFVHVFGAYFGLTVARMLYRPEVSDSTKEGANYHSDLFSMIGTVFLWMYWPSFNSALAVADGRYRAVINTYLSLAASAVVTFAISSAVDKDGKVNMVHVQNATLAGGVAVGTTADMMIQPWGALLIGTAAGTLSTMGFTYVQPFLARHKLHDTCGVNNLHGMPGLLAGVAGAVASRLKLCDLGERSARDQAEFQMAALFVTLGVAVLGGAVTGIILQLPVWDRLQAEELFDDRGYWEVPDDEYDDSEPEPRAQSHPRAPSVIQDAE

*Branchiostoma floridae* RhR2

>gi|299823645|dbj|BAJ10274.1| Rh-related protein [Branchiostoma floridae]

MAWRRGKLTAILLIFEVVFIVLFGILVVYDDQANATATQNSLTPAQGGADADNNVLRAYYPMFQDVHVMMFIGFGFLMTFLKKYGFGSVGFNFLVAAFVLQWATLMSGFLHLHHGKIVVDITTLLTSDFAAAAVLISMGAVLGKVSHVQLIIMAFVEIIFFSINEWVGLTFFKAVDVGGSMFVHAFGAYFGLAVARVLYREDMVGHSKEGSVYHSDIFAMIGTVFLWLFWPSFNAALAPGDDQHRAVINTYFSLAACAVVTFAISSAVEKDGKVNMVHVQNATLAGGVAIGTSADMMVHPWGALLIGSIAAVLSVVGYSYITPFMADKLKIHDTCGVHNLHGMPGVLAGIIGAIVAAVASPAQYGNSVYQIFPARAPIENSTELATIQAAFPAIEAGNGRSAGLQAGFQMAALIVSVLVALVTGALTGLLLRLPIWDHPEKDSMYEDEDYWEIPEEEAVDMNGSLEGVGHKKPDSRARTESDTNNEQDTKV

*Caenorhabditis elegans* RhR1

>gi|17563228|ref|NP_504436.1| RH (Rhesus) antigen Related [Caenorhabditis elegans]

MRSPLHQNQLTLILGLFQVVFLVIFALYGSYDASALPSETKNVEEAARMTNLYPLFQDTHVMIFIGFGFLMTFLKRYGFSAVSINMLLAVFTIQWGIIVRGMASAHHGFKFTISLEQLLTADFAAAVILISMGAMLGKLSPSQYVIMAFFETPVALIVEHICVHNLQINDVGGSIIVHAFGAYFGLACAKGFGKKEQRGHTNEGSTYHTDIFAMIGAIFLWIYWPSFNAAVAATDDARQRAVANTFLSLCACTMTTFLVSQAVDKHKRFDMVHIANSTLAGGVAIGTTANVVLEPYHAMIIGVIAGAVSVIGYKYITPFLSEKLGIHDTCGVNNLHGMPGLIAGFASIAFLFIYDETRYPAQYDKIYPGMARGEDRTRMFDEKTQALNQLMAIGLVFLASTVSGYLTGLLLKLKIWDQVRDDEYYADGDYFETPGDYDFTSRIVTSVKQIEVAEYNPLSQKEV

*Caenorhabditis elegans* RhR2

>gi|9739015|gb|AAF97865.1| Rh-like protein-2 [Caenorhabditis elegans]

MRSPLHQNQLTLILGLFQVVFLVIFALYGSYDASALPSETKNVEEAARMTNLYPLFQDTHVMIFIGFGFLMTFLKRYGFSAVSINMLLAVFTIQWGIIVRGMASAHHGFKFTISLEQLLTADFAAAVILISMGAMLGKLSPSQYVIMAFFETPVALIVEHICVHNLQINDVGGSIIVHAFGAYFGLACAKGFGKKEQRGHTNEGSTYHTDIFAMIGAIFLWIYWPSFNAAVASTDDARQRAVANTFLSLCACTMTTFLVSQAVDKHKRFDMVHIANSTLAGGVAIGTTANVVLEPYHAMIIGVIAGAVSVIGYKYITPFLSEKLGIHDTCGVNNLHGMPGLIAGFASIAFLFIYDETRYPAQYDKIYPGMARGEDRTRMFDEKTQALNQLMAIGLVFLASTVSGYLTGLLLKLKIWDQVRDDEYYADGDYFETPGDYDFTSRIVTSVKQIEVAEYNPLSQKEV

*Capitella teleta* 7

>gi|443734385|gb|ELU18387.1| hypothetical protein CAPTEDRAFT_137751 [Capitella teleta]

MWKRGKFFVFICSFQLLFLVLFAIFGEYDLTAKPNAQINESIGGQNEVHHFYPMFQDVHVMMFIGFGFLMTFLKKYGLSSVGENMMLAAFVLQWATIIGGFLHLHDNKFYVTMETLLVSDFASAAVLISMGAMLGKASPLQYIIMAFFEIVLFQVNEWIGLTHFKVADVGGSIFVHVFGAYFGIACSRMLYKKDDMEEAEEKEGSVYHSDHFAMIGTVFLWLFWPSFNSAVAADGDDQLRAIINTYFCLAACCVTTFAVSSLIEKDKFLMEHIQNATLAGGVAIGSMADMDVKLWGAILIGMAAGTLSVLGYKYVGPFLQRRLGIHDTCGVNNLHGMPGIMAALGSVIVTAIATEENYGNSLFGIWSERDPENSNRSAREQAGYQAAALGVTLLIAIVGGCFTGLILRLPVICDPLKAHENFDDTAFWVMPGGYPGEIKTSIEELPEKKNDADHVSKI

*Capitella teleta* 8

>gi|443691773|gb|ELT93531.1| hypothetical protein CAPTEDRAFT_104921 [Capitella teleta]

MALRNKKTAILLVICQIVFIILFGVFVDYAPDADARHPGNSHDQNGTDHENNSLKMYYPMFQDVHVMIFVGFGFLMTFLRRYGFGAVGLNFLLAAFVIQWSLLVQAFFEVGQHGGGRFEIDVTTMLTSDFACAAVLISFGSLLGKASPIQLITMALIEIVIFTINEHIGVGLFSAVDVGGSMYVHAFGAYYGLAVARVLYKNSHKENPKEESNYHSDLFAMIGTIFLWMYWPSFNSALAPADDQHRAVLNTYLALAACCVVTFAISSLTDAKGHWDMVHIQNATLAGGVAVGTSADMMLHPFGALMVGSVAGMISTLGYKYLTPFMANRLKIHDTCGVHNLHGMPGVMAGVIGAIMAGIASTQEYEYEDIHEVFSLVQPGIGRSAMAQGAYQALALAVTLVFAIVTGALTGLLLRLKIFDQPEGGMIHDDAAWWGTPEDFTVEVVNGNKSAKGLMTEMEDK

*Chlamydomonas reinhardtii* RHP1

>gi|159474702|ref|XP_001695464.1| Rh protein, CO2-responsive [Chlamydomonas reinhardtii]

MQALPPKIPASVSGHGTQSRRHSLDWSHIGLPSRETQLRAGFVPSAAVVIVIFVGLFFGLTQYTELGTNAQEEVDRFYKYLVDVNIMVWIGFGFLMTFMRRYGYGAVALNYFASALMFLEAILMIGATQQVFWNYHRTKIQIDIALLIDCAFCAASGMIAFGAIIGKATPTQLLWLLFWQVPLYALNQQLVIHTFKALDMGGTIVIHLFGAYYGLAASLMISRKQPLHGLDNPKNSGAYLNDIFSMIGTIFLFIYWPSFNGALASVSAGHMEEATDAKKAAQFLSIVNTLLSLLGAGLSVFATSALVGGRFNMVHIQNSTLAGGVAMGAACTLRLTPGGALAVGLGAGAISTLGFQYLMPFLDRTIGLGDTCGVHNLHGIPAIVGTLVAGLAALGQHPDYLEHDTGRQQLGYQVLAGVVTMGIAIAGGLLGGFVVSWFNPRGDDPLTVPELFDDGPWWEHQRVEPMPISTSIHLSNMSAHGKSHHNQSVSVGQLNPIREGREIAVSGVPATGQRSVGEIAVTMQAAPVMASSAPVMGMHAAAATPIDTPLFADGHAMENAARPVQPMVAGAGNV

*Chlamydomonas reinhardtii* RHP2

>gi|159474704|ref|XP_001695465.1| Rh protein [Chlamydomonas reinhardtii]

MSSVLKIPTAMASGAASRRHSLDWSHVGLPPREPQLRTGFAPSVIAIAAVIVGLFFGLTQYTELAENAQEQVERYYKYFIDVQVMIFIGFGFLMTFMRRYSYGAVSLNYFASALMFLEAILMIGATQQVFWNYHRTKIQIDMALLIDCAFCAGSGMIAFGAIIGKATPTQVLWLLFWQVPLYALNQQLVIHTFKALDMGGTIVIHLFGAYYGLAASLMISRKQPLHGLDNPKNSGAYLNDIFSMIGTIFLFIYWPSFNGALASVAYANQTSATYAEKSAQFLAIVNTLLSLLGAVLSVFATSALVGGRFNMVHIQNSTLAGGVAMGAACTLRLTPGGALAVGLGAGAISTLGFQYLMPFLDRTIGLGDTCGVHNLHGIPAIVGTLVAGLAALGQHSDYLEHDTGRQQLGYQVLAGVVTMGIAIAGGLLGGFVVSWFNPCRDETMALPELFDDGPWWVGQRVEPMPISTSIHLSTLNSRHQKDGGSTQRGNAANASVSMANTLVPNPAEPRRHGSACPAAPIAVSGVPAAVAHHSLTVSGQQGAAHGAAAASEIVASVPFLAFQQQQQQMAAAAAAAHQYPQPFMSTAVLMGGAAGGGGANNDRPLFSDDVIGMETALAPVQPMQQPAYSQASAGPNNV

Chlorella sp. NC64A, Rh

>Chlorella sp. NC64A, Rh (JGI 21763)

MDAEAGTPLLDQAHGHGTPWNLRGTFGASLAGLTALLLGLLAGFGRFAPDIDDTHVGQYYSYLTDVYVMIFLGFGFLMTFLKRYSYSAVSLNYVTSCLVILEAVLACGWAQQGWGAVAVDLPLLIDAAFAAGAAMISFGAVLGKATPAQLVWLLALEVPLYAANAQLVAGRWGALDVGGSITIHAFGAVYGVAAAAFLAPRGSGSAHPKSGASYVSDMTAMLGTIFLFIYWPSFNGALASAPGEKAQPQVYCIMNTVVALLGAVLAAFAASAAATGKLDMVHIQNATLAGGVAIGSSANLAMPPACALAVGITAGALSTAGYLVLSPFLEGKCGITDTCGVANLHGAPGIWGGLASALFSWLFAAGANKKLIVHGASQPAVQLAALGCTLAAAAAGGALAGFLVSKADPAKQSLEEGDLYEDAVFWHEVEGEEHKEE

*Ciona intestinalis* RhAg

>gi|32765784|gb|AAP87368.1| RhAG-like protein [Ciona intestinalis]

MRGKFSGFLLLPQILFIILFGVFVEYDTVAGPRNASSTAGIEEFDNLYPMFQDVHVMIFIGFGFLMTFLKRYGFGSVGFNFMLAAFVIQWTILMRGCLDLEDIKDGKINISLESFLNADFACGAVLISFGAVLGVLGPIQLIVMAVLEVVFYSVNEFVIAHKFQISDIGGSMVIHTFGAYFGLAVSLMHWKPEFKNSHPKEESVYHADIFAMIGTLFLWLFWPSFNGAPAAGSIIGERAVINTVFSLAAAGVCGFAMSSIVDKSGKISMVHVQNATLAGGVAAGAVANLKLGPLGAIIIGSASGLLSVAGYAYITPAIQKHMKIHDTCGVHNLHGMPGVLSGFAAVVFAKLTTATTYTDIELAAIYPGRVNNLDFQQQANLQLAGLAVTLGIAIFAMCCIAQIYCIKTFV

*Ciona intestinalis* RhBg

>gi|699241148|ref|XP_009858227.1| PREDICTED: rh type B glycoprotein isoform X1 [Ciona intestinalis]

MSNTRGKATALLLVAQAILLVLFGLFVDYDVAAGPRNNSLTTHTTLAHYYPIYQDVHVMMLIGFGFLMTFLKRHGFGSVGFNFLLTCYVIEWSTLVNGWFGMIGSNEGRILIDIKSLLEADFAVAAVLISFGAILGVASPVQLIVMATIEVVCYNVSIYVGIVKLQVTDVGGSMFIHAFGAYFGLAVARVLYKKSQTLSKNEGSEYHSDIFAMIGTLFLWLYWPSFNAGPASGTERHRAVINTVLSLSACTVVTFALSAVTDKKNKLDMVHIQNATLAGGVAIGASADLIVQPFGALLVGSTAATVSTLGFRYLQPVLQRNIKLHDTCGVHNLHGMPGILGAVISAIVATIATKDAYQDSYHELFKDASRTSSQNGGYQIAALVCVLCIALLSGTLTGFLLKLPVWDNLSAEELFEDEVFWDCPEEIEEKKSETEKTINKSDHENEDLLVPSA

*Ciona intestinalis* RhCg

>gi|699241144|ref|XP_009858225.1| PREDICTED: rh type C glycoprotein isoform X1 [Ciona intestinalis]

MGNTRGKATALLLVAQAIILVLFGLFVDYDVAAGPRNNSLTTHTTLAHYYPIYQDVHVMMLIGFGFLMTFLKRHGFGSVGFNFLLTCYVIEWSTLVNGWFGMIGSNEGRILIDIKSLLEADFAVATVLISFGAVLGVASPVQLIMMATIEVVCYNVSIYVGIVRFQVTDVGGSMFIHAFGAYFGLAVARVLYKKSQTLSKKEGSEYHSDIFAMIGTLFLWLYWPSFNAGPASGTERHRAVINTVLSLSACTVVTFALSAVTDKKNKLDMVHIQNATLAGGVAMGASADLIVQPFGALLIGSVAALVSTLGFKYLQPILQRNIKLHDTCGVHNLHGMPGILGVVASAIAAAIATQETYKDSYHELFKNTTRTSGQNGGYQIASLAVVLGIAIIGGTLTGFLLKLPVWDNLSAEELFEDEVFWDCVETVSEDEAEQNGSNDKQHEVVA

*Drosophila melanogaster* Rh50

>gi|161078314|ref|NP_001097800.1| ammonium transporter [Drosophila melanogaster]

MNNGTVGAPRNHSNYVIPGLYDLSVEDTNWVLTSSFIIFTMQTGFGMLESGCVSIKNEVNIMMKNVIDIVLGGFTYWLFGYGMSFGRGPLSNPFIAIGDFLLDPPVGDALMGQIFAAFLFQLSFATTATTIVSGAMAERCNFKAYCLFSFLNTAVYCIPAGWVWGEHGFLNKLGAVDIAGSGPVHLIGGASAFASAAMLGPRLGRYSEGYDPLPLGNPVNACMGLFVLWWGWLAFNSGSTYGVSGAKWQYAARAAVMTMMGSFGGGFTSSIYSFWRHGGGMDIMDLINGVLGSLVSITAGCFLYRAWEALVIGAIGSLFCVLAMPLFDRMGVDDPVGASAVHGVCGIWGVIAVGLFADNPIPLDTTNGRSGLFKGGGWYLLGIQTLSALCLACWGVCSTFLLLYVINKIIPIRMDPHEELLGADLTEHRIRHSQIGLSRAISALAPIKVDLKDIAGIQPIGINPGHERSIDQLRAAEDKLQQWQSYLEQVSGSRANVKMDAGQSHADINFKPRTAGNVFNRRIPARKSISGGLYRSNNSELPVIGSVMGQKPEKDPNFAWVD

>gi|506316960|ref|WP_015836735.1| ammonium transporter [Geobacter sp. M21]

MKKCICYLLVMVTVLLLAGVAFASGSVGTNLQELSDVQKYNRAIHIMAMLLVGFGFLMVFVKKYGRSAITATYLLTSVAIPLYFLKDSLFPPLAEESVIDKLILAEFAAASLLICAGAVLGRLKMNQYLLLGILFVPFYSLNEWLVLNGGLGLITGKVVDTGGSIVIHAFGAIFGLAVAATMTTQEEYATPIECDDTSDRYSLLGSMVLWVFWPSFCAALVAPADVPRTAVNVILALCGSTLATYFATVRLRGKISAADIANATLAGGVAIGSTCDLATPAAAFIIGIMAGVISTFGFAVIQGRLTDLLKKVDTCGVLYLHGMPGIFGGLAALFIVTGINSGAQLTAIALTAVLAATAGLVSGKVIALFGHRAEPYTDAEEFEGEEVEEELFPVSAVPELDTE

*Homo sapiens* RhAg

>gi|156627565|ref|NP_000315.2| ammonium transporter Rh type A [Homo sapiens]

MRFTFPLMAIVLEIAMIVLFGLFVEYETDQTVLEQLNITKPTDMGIFFELYPLFQDVHVMIFVGFGFLMTFLKKYGFSSVGINLLVAALGLQWGTIVQGILQSQGQKFNIGIKNMINADFSAATVLISFGAVLGKTSPTQMLIMTILEIVFFAHNEYLVSEIFKASDIGASMTIHAFGAYFGLAVAGILYRSGLRKGHENEESAYYSDLFAMIGTLFLWMFWPSFNSAIAEPGDKQCRAIVNTYFSLAACVLTAFAFSSLVEHRGKLNMVHIQNATLAGGVAVGTCADMAIHPFGSMIIGSIAGMVSVLGYKFLTPLFTTKLRIHDTCGVHNLHGLPGVVGGLAGIVAVAMGASNTSMAMQAAALGSSIGTAVVGGLMTGLILKLPLWGQPSDQNCYDDSVYWKVPKTR

*Homo sapiens* RhBg

>gi|224548945|ref|NP_065140.3| ammonium transporter Rh type B isoform a [Homo sapiens]

MAGSPSRAAGRRLQLPLLCLFLQGATAVLFAVFVRYNHKTDAALWHRSNHSNADNEFYFRYPSFQDVHAMVFVGFGFLMVFLQRYGFSSVGFTFLLAAFALQWSTLVQGFLHSFHGGHIHVGVESMINADFCAGAVLISFGAVLGKTGPTQLLLMALLEVVLFGINEFVLLHLLGVRDAGGSMTIHTFGAYFGLVLSRVLYRPQLEKSKHRQGSVYHSDLFAMIGTIFLWIFWPSFNAALTALGAGQHRTALNTYYSLAASTLGTFALSALVGEDGRLDMVHIQNAALAGGVVVGTSSEMMLTPFGALAAGFLAGTVSTLGYKFFTPILESKFKVQDTCGVHNLHGMPGVLGALLGVLVAGLATHEAYGDGLESVFPLIAEGQRSATSQAMHQLFGLFVTLMFASVGGGLGGLLLKLPFLDSPPDSQHYEDQVHWQVPGEHEDKAQRPLRVEEADTQA

*Homo sapiens* RhCg

>gi|7706683|ref|NP_057405.1| ammonium transporter Rh type C [Homo sapiens]

MAWNTNLRWRLPLTCLLLQVIMVILFGVFVRYDFEADAHWWSERTHKNLSDMENEFYYRYPSFQDVHVMVFVGFGFLMTFLQRYGFSAVGFNFLLAAFGIQWALLMQGWFHFLQDRYIVVGVENLINADFCVASVCVAFGAVLGKVSPIQLLIMTFFQVTLFAVNEFILLNLLKVKDAGGSMTIHTFGAYFGLTVTRILYRRNLEQSKERQNSVYQSDLFAMIGTLFLWMYWPSFNSAISYHGDSQHRAAINTYCSLAACVLTSVAISSALHKKGKLDMVHIQNATLAGGVAVGTAAEMMLMPYGALIIGFVCGIISTLGFVYLTPFLESRLHIQDTCGINNLHGIPGIIGGIVGAVTAASASLEVYGKEGLVHSFDFQGFNGDWTARTQGKFQIYGLLVTLAMALMGGIIVGLILRLPFWGQPSDENCFEDAVYWEMPEGNSTVYIPEDPTFKPSGPSVPSVPMVSPLPMASSVPLVP

*Homo sapiens* RhD

>gi|20336225|ref|NP_057208.2| blood group Rh(D) polypeptide isoform 1 [Homo sapiens]

MSSKYPRSVRRCLPLWALTLEAALILLFYFFTHYDASLEDQKGLVASYQVGQDLTVMAAIGLGFLTSSFRRHSWSSVAFNLFMLALGVQWAILLDGFLSQFPSGKVVITLFSIRLATMSALSVLISVDAVLGKVNLAQLVVMVLVEVTALGNLRMVISNIFNTDYHMNMMHIYVFAAYFGLSVAWCLPKPLPEGTEDKDQTATIPSLSAMLGALFLWMFWPSFNSALLRSPIERKNAVFNTYYAVAVSVVTAISGSSLAHPQGKISKTYVHSAVLAGGVAVGTSCHLIPSPWLAMVLGLVAGLISVGGAKYLPGCCNRVLGIPHSSIMGYNFSLLGLLGEIIYIVLLVLDTVGAGNGMIGFQVLLSIGELSLAIVIALTSGLLTGLLLNLKIWKAPHEAKYFDDQVFWKFPHLAVGF

*Lottia gigantea* 5

>gi|676492623|ref|XP_009065981.1| hypothetical protein LOTGIDRAFT_133634 [Lottia gigantea]

MRSPTRLKFPIVVLGIQIIFIVLFALTVEYDKSADAKFDIQHPRNVTDRNPPENEVSKYYPMFQDVHVMIFIGFGFLMTFLKRYGFSAVGLNLLIAALILQWATLVQGYLHSDGNVHISITSMITADFAAATVLITFGAVLGKVSPLQMVVVALIEVVLATLNEKVGVQIFQVSDIGGSIFVHAFGAFFGLALARVLYDEDVERSTKEGSVYHSDLFAMIGTIFLWLFWPSFNSALAVGDDQHRAVLNTYMALAACCIVTFAISALVDGEGKFDMVHVQNATLAGGVAVGTSADMMIQPVGALVVGAVAGVLSTCGYRFITPFLTSKLKLHDTCGVNNLHGMPAVLAAVIGSIAAAVANTDTYDDPEFAKIKLYVDVSPGEGRTAGQQGGYQMLALAVTLAIAIVGGVITGFIIKFIPMFVAPKKEQLFEDEPYWGVPDDHRTYPNIPIIITTEATDSHASVPSLYQHVTARLLESDEPVNSERHSNSSLEDEKESIENKTADIDV

*Lottia gigantea* 6

>gi|676434770|ref|XP_009047338.1| hypothetical protein LOTGIDRAFT_111301, partial [Lottia gigantea]

YFSVFQDVHVMMFIGFGFLMTFLKRYGFSAVGINMLVAAFCLQWATIVRGIIHGDVLSGGTFKVHVGEMLSADFAAATVLISFGAVLGKTSPLQLLVMALIEILLAQINEYVGVDIFHAVDIGESMYVHAFGAYFGLAVARVLYHEEVEHSSKEGSVYHSDLFSMIGTVFLWLYWPSFNGGAAEGDEQHRAVLNTYFSLAACCIVTFAISALTDSKGKFDMVHVQNATLAGGVAVGTCANMPMEPWGALVLGTVAAIISVLGYKYITPFLSSKVKLHDTCGVNNLHGMPGLLAGIAGAVMAAMATQEKYGESVAHGSYGLGRNASSQGGYQAIALVVTLGVAIVGGVITGFILKLPIWDNVKQRDSFFEDDEQWETPKEYELPTIARYEKNSKELDTNM

*Nematostella vectensis* 9

>gi|156375207|ref|XP_001629973.1| predicted protein [Nematostella vectensis]

MSRPTFTILTILLQVLFVVIFAIFGEYGDDARPNHKRPNPGAAGINAVNIYYPMFQDVHVMIFLGIGLLLAFLRKHAYSSISYCFFAAAILCEWSTIINGVFWHIIEGGNDKFKIDLFSAINADFAAAVILISYCVVLGKISILQLLVMGVIELAVYVLNSWICFAKLGISDIGASITIHMFAAYFGLGVTRVLHSRDSEGNGKECSSYHNDVFCLVGTIFLWLYWPSFNACLTTDDVMRHRTVTNTYYSMLGACVMVFALSPMFRRDGKFNLSHVQNATLAGGVAIGTASNMIVQPWGSMLIGSIGGAMCTLGYVYLSPFLKKHCKMHDVCGVHNLHALPAFLSGIASAIASSLAAADEYGDATPGQQAGFQVAAMFVTMGISLLSGILTGKEVYIL

*Nematostella vectensis* 10

>gi|156375209|ref|XP_001629974.1| predicted protein [Nematostella vectensis]

MSRPTFTILTILLQVLFVVIFAIFGEYGDDARPNHKRPNPGAAGINAVNIYYPMFQDVHVMIFLGIGLLLAFLRNHAYSSISYCFFAAAILCEWSTIINGVFWHIIEGGNDKFKIDLFSAINADFAAAVILISYCVVLGKISILQLLVMGVIELAVYVLNSWICFAKLGISDIGASITIHMFAAYFGLGVTRVLHSRDSEGNGKECSSYHNDVFCLVGTIFLWLYWPSFNACLTTDDVMRHRTVTNTYYSMLGACVMVFALSPMFRRDGKFNLSHVQNATLAGGVAIGTASNMIVQPWGSMLIGSIGGAMCTLGYVYLSPFLQKHCKMHDVCGVHNLHALPAFLSGIASAIASSLAAADEYGDATPSQQAGFQVAAMFVTIGISLLSGILTGFLIKLWIFEPMSTRQMFDDEDFWMVSKC

*Nematostella vectensis* 11

>gi|156352536|ref|XP_001622804.1| hypothetical protein NEMVEDRAFT_v1g139792 [Nematostella vectensis]

MADDTFGRGKFPCLLILFQGLLLILFVVFVDYSKDVLPDAQNTLKINTDFPVFQDIHVLLLVGFGLLMTFLKKYGFSGLGYNFLICAVVMEWATLMQGFFEMENNKIQLDIDSMIKGDLAAVTILVSFGAVLGKTSSLQLLLMSFIEVIFYTINRTICTTYLQAVDPGGAVLVHAFGAYFGLTVARVLYNKNSVAHEKEASRYTSDLFATLGTIFLWVYWPSFNSILVQGVLRHRAVVNTYYAMAASCVTVFAFSSLVTKASKLSMVHIQNATLAGGVAVGTIASMIIHPWGAILLGMVGGIISTIGYKYIQPWLIRTLHIHDTCGVNSLHGMPGILAAIASIVSSGLAGFGQYKTSLYTVFPARSPVINSTQYLELVQFVPDARAGLGRTATEQAGYQCAALVMTLAVSSMGGLITGFIVKQSFFDPMTEDRMYDDLHYWEVCFYHFSIF

*Saccoglossus kowalevskii* 2

>gi|585710448|ref|XP_006824549.1| PREDICTED: ammonium transporter Rh type B-like [Saccoglossus kowalevskii]

MAQTVEPKRRRGKLTLVLVIFQIIFLVLFGIFVEYGEDSDAKAEQNNLEHGNGGADAMDNRLGYYYPMFQDVHVMIFIGFGFLMTFLKRYGFGSVGYNFLLAAFVIQWATLMQGFLHLHHGKIQVGVETMLTSDFAAAAVLISFGALLGKVSHLQLIILGFVEIIFFSVNEYVGAGYFQTADVGGSMFVHVFGAYFGLAASRVLNKKSWEGSSKEGSVYHSDLFAMIGTVFLWMFWPSFNSALAPGDDQHRAVINTYYALASCCVTTFAVSAMVSKENKLDMVHIQNSTLAGGVAVGTCANMMIQPWGAMVIGVVAGTLSVVGYKWISPFLLSKVHLHDTCGVNNLHGMPGILAAIAGAIAAATAETSNYGYGLYEVFPARAPLVNSTALDTILDDFTVDAGSGRTAGMQAGFQLVALVITLAIAIISGMLTGLLLKIPILDQLTPDELYDDNNFWEVPEEDTHYVKVQVDDQMKGSMDELNGATNKTEKDTKM

*Strongylocentrotus purpuratus* Rh50

>gi|115733130|ref|XP_789738.2| PREDICTED: similar to Rh type C glycoprotein-like protein [Strongylocentrotus purpuratus]

MGFFRGKLTTFLILFEILFIVLFAVFVRYDDVASPGATGKEGYYQVDRYYAYFQDVHVMMFVGFGFLMTFLKRYGYSSVGFNFLLAAFVIQWATLFRGFVELGHSDDGKIHVSITSMLLADFTSATILISFGALLGKTSPLQLTIMAFFEVIFSLVNEFIAVEFLGTTDIGGSMYVHTFGAYFGLAVSMVVTRQAGRESPKEGAVYHSDLFAMIGTLFLWIFWPSFNSALGDDDQRHRAVLNTYFSLSACCVATFATSSLLSKKGKFDMVHVQNATLAGGVAVGTAANLMIQPWGAILIGVVAGVLSTFGYKVIQPFLCEKIGLHDTCGVNNLHGMPGILGGLAGVVACALASESVYGLILRCVPGLDALKDEKLMEDEYFWEEVEGEEGAQQTTVTNGKEADAQV

*Trichoplax adhaerens,* 4

>gi|196016158|ref|XP_002117933.1| expressed hypothetical protein [Trichoplax adhaerens]

MTVFEIVALILFAVFVQFDSVALPTSHAESFHGVSYAMFQDIHVMIFVGFGFLMTFLKKYSFSSVGFNLLLASFVIQWSTLTLGFCHSWSNFSHILIDTESLIAADICAAAILISFGAVLGKTSTLQLIVMAFFEVIFYSINETLCLQYLKISDMGGSIVIHAFGAYFGLAVAFVLYSRDAVGHANEGSSYQSDISAMIGTVFLWIFWPSFNAGLASGDQQQRAVFNTYFALAACTCTSFVVSSLLDREGRYRMVHIQNSTLAGGVAVGASANMLLPIWLSMLTGIIAGVVSVIGYDYISPFLAKKRIHDTCGVNNLHGMPALIASVLSAIIAATAHFSTAMQAAGIGVTLGISILSGLITGYIIKFSIFDDVDSQDFHLDSPHWEVTIKR

*Trichoplax adhaerens,* 5

>gi|196016156|ref|XP_002117932.1| hypothetical protein TRIADDRAFT_33274 [Trichoplax adhaerens]

MGILSRKLVIFMTLFELVAIILFAVFVRFDKVALPGSLAVSIPGVTYAMFQDIHVMIFIGFGFLMTFLKKYSFSSVGFNLLLASFVIQWSTLMLGFCHHWSNFSIITIKTEDLIGSDICAAAVLISFGAVLGKTSPLQLMVMALLEVIFYAVNETICFQYLKISDMGGSIVIHAFGAYFGLAVALVMNKKEAVDNPNEGSSYQSDLSAMIGTVFLWIFWPGFNAGLASGDQQQRAVFNTYFALAACTCTSFVISSLIDREGRYRMVHVQNSTLAGGVAVGASANMLLPIWLSMLTGIIAGIISVIGYDYITPFLAKKRIHDTCGVNNLHGMPALIAAILSIIMASLAHFNQYEQRQVRRDYNFNNSISDSYIVTKFG
